# Supplementary material for: Movement dynamics of divisome proteins and PBP2x:FtsW in cells of Streptococcus pneumoniae
Source: Proc Natl Acad Sci U S A. 2019 Feb 4;116(8):3211–20. doi: 10.1073/pnas.1816018116 (PMC6386697; doi:10.1073/pnas.1816018116)
Supplement: Supplementary File [file pnas.1816018116.sapp.pdf]

SUPPORTING INFORMATION (SI) APPENDIX

Movement Dynamics of Divisome Proteins and PBP2x:FtsW in Cells of *Streptococcus pneumoniae*

Amilcar J. Perez, Yann Cesbron, Sidney L. Shaw, Jesus Bazan Villicana, Ho-Ching T. Tsui, Michael J Boersma, Ziyun A. Ye, Yanina Tovpeko, Cees Dekker, Seamus Holden, and Malcolm E. Winkler*

Short title: Pneumococcal FtsZ Ring and PBP2x:FtsW Dynamics

SI APPENDIX, EXPERIMENTAL PROCEDURES

SI APPENDIX, TABLE S1: Bacterial strains and oligonucleotides used in this study

SI APPENDIX, FIGURES AND LEGENDS: Fig. S1-S28

SI APPENDIX, MOVIE LEGENDS

Corresponding author:
Malcolm E. Winkler
Department of Biology
Indiana University Bloomington
1001 E. 3rd St.
Bloomington, IN 47405 USA
Phone: 812-856-1318
E-mail: winklerm@indiana.edu

www.pnas.org/cgi/doi/10.1073/pnas.1816018116

SI APPENDIX, EXPERIMENTAL PROCEDURES

Bacterial strains and growth conditions. Bacterial strains used in this study (Table S1) are isogenic, unencapsulated (Δ*cps*) derivatives of *Streptococcus pneumoniae* (*Spn*) serotype 2 strain D39 (1). Streptomycin-sensitive (*rpsL1*) strain IU1945 and streptomycin-resistant (*rpsL1*) strain IU1824 were used as parents for various constructs (SI Appendix, Table S1) (1). Strains containing antibiotic resistance markers were constructed by transformation with linear DNA amplicons synthesized by overlapping fusion PCR or Gibson isothermal assembly (NEB C#E2611S) into competent pneumococcal cells as described previously (2). Bacteria were grown on plates containing trypticase soy agar II (modified; Becton-Dickson) and 5% (v/v) defribrinated sheep blood (TSAII-BA). Plates were incubated at 37° C in an atmosphere of 5% CO_2. For antibiotic selections, TSAII-BA plates contained 250 µg kanamycin mL^{-1}, 150 µg spectinomycin mL^{-1}, 0.3 µg erythromycin mL^{-1}, 250 µg streptomycin mL^{-1}, 2.5 µg chloramphenicol mL^{-1}, or 0.25 µg tetracycline mL^{-1}. For storage, strains were cultured statically in Becton-Dickinson brain-heart infusion (BHI) broth at 37° C in an atmosphere of 5% CO_2, and growth was monitored by OD_{620} as described before (3), unless otherwise noted. Bacteria were inoculated into BHI broth from frozen cultures or colonies, serially diluted into the same medium, and propagated for 12-16 h. For growth determinations and TIRF microscopy, bacteria were grown in C+Y media (4, 5), adjusted from pH = 7.9 to pH = 7.1 by addition of 500 µL of 1 M HCl to 43.9 mL to C+Y, pH 7.9. After incubation for 3-4 h in an atmosphere of 5% CO_2, the pH of C+Y decreased from 7.9 to 7.6 or 7.1 to 6.9. For growth experiments, overnight cultures that were still in exponential phase (OD_{620} = 0.1–0.4) in BHI broth in 5% CO_2 were washed

once with C+Y, pH 7.1 and resuspended in C+Y, pH 7.1, diluted back to OD_{620} = 0.001-0.012 in C+Y, pH 7.1 to start final cultures in 5% CO_2 that lacked antibiotics.

Western blotting. Western blotting was performed by a modification of the protocol in (6). Briefly, cells were grown in BHI broth or C+Y, pH 6.9 to early exponential phase (OD_{620} = 0.09-0.18). Cells were centrifuged, washed once with 4° C PBS, and centrifuged again, and pellets were placed on dry ice for rapid freezing and stored at -80° C for 1-24 h. Pellets were thawed for 10 min and suspended in 100 µL of deoxycholate-containing-SEDS lysis buffer (6), vigorously vortexed, and placed at 37° C for 15 min with shaking at 300 rpm. Protein concentration was determined (BioRad DC™) within the range of a standard curve (0.1-1.2 mg mL^{-1} BSA). Samples were diluted with 2X Laemmli sample buffer with *β*-mercaptoethanol and boiled for 10 min. The amounts or protein indicated (SI Appendix, Fig. S4) were loaded onto a 4-15% pre-cast gradient SDS-PAGE gel (Bio-Rad), subjected to electrophoreses, and transferred to a nitrocellulose membrane. Blocking was carried out for 20 min at room temperature in phosphate-buffered saline with tween 20 (PBST) containing membrane blocking agent (GE Healthcare). Immunoblotting was performed for 1 h using the following primary antibodies in PBST: rabbit anti-GFP (5:6000; ThermoFisher #A11122); rabbit anti-FtsZ (1:6000) (7); rabbit anti-bPBP2x (1:10000) (8), and rabbit anti-HaloTag (5:6000; Promega #G9281). Addition of secondary antibody and chemiluminescent signal detected were performed as described in (6).

2D-epifluorescence microscopy and demograph generation. Strains were grown in C+Y, pH 6.9 at 37° C in 5% CO_2 to OD_{620} = 0.08-0.18. 500 µL of culture were concentrated by centrifugation (21,000 × *g* for 5 min at 25° C), resuspended in 25 µL of

C+Y, pH 7.1 (normal atmosphere), and 1.5 µL was immediately imaged by 2D-epifluorescence microscopy (3). Strains expressing HaloTag (HT) fusions were labeled with HT-TMR ligand (Promega #G8252) as indicated in SI Appendix, Figure S2. Demographs showing protein fluorescence intensity as a function of cell length were generated by using Microbe J (version 5.11s) (9) with the following parameters: (area [μm^2] 0.53-max; length [µm] 0.5-3.2; width ([µm] 0.2-max; circularity [0-1] 0-max; curvature [0-max] 0-max; sinuosity [0-max] 0-max; angularity [rad] 0-0.38; solidity [0-max] 0.75-max; intensity [0-max] 0-6200; Z-score 2.0-max). Occasionally, cells with regular shapes and sizes that had been excluded from analyses due to close proximity to other cells were manually added back to analyses using the microbe tool "particle cutter." In addition, late-divisional cells that were automatically counted as two separate cells were examined for their degree of separation, since previous studies show that some late-division cells retain proteins at septa and are not fully divided (3). If a pair of pre-divisional cells was located next to each other and their medial axes were approximately aligned, they were counted as a single cell. If the medial axes were offset by an angle of approximately 10 degrees or greater, the cells were counted as two separate cells. Late-division cells that were not fully divided were joined in MicrobeJ and included in data sets as single, long cells. In generation of demographs, a small number (<5 per data set) of cells that showed comparatively high fluorescence intensities were removed to allow for a maximum range of protein localizations in heat maps. For strains IU13315 and IU14375, the maximum fluorescence intensity of demographs was normalized to half of the original maximum fluorescence intensity, allowing for better

visualization of FtsZ-sfGFP localization throughout the whole population, where there was some heterogeneity of FtsZ-sfGFP expression.

Growth and imaging of live cells by TIRFm. Live cell imaging by TIRF microscopy (TIRFm) was adapted from reference (10). Briefly, coverslips were acid-ethanol soaked (25% concentrated HCl, 25% H_2O, and 50% ethanol) for 12-24 h, washed with sterile H_2O by submersion 5X, followed by repeating the washing steps with fresh H_2O. Coverslips were dried by rubbing with lens paper on both slides. About 1 h prior to viewing cells by TIRFm, agarose pads (1.5% (wt/vol); Sigma BioReagent #A9414) containing C+Y, pH 7.1 were prepared on glass slides as described previously (10). Gene Frames (1.0 cm x 1.0 cm) (Thermo-Fisher # AB0576) were used to create agarose pads for this study. When needed, zinc plus 1/10 concentration of manganese was added at the indicated concentrations to C+Y medium prior to melting agarose. At OD_{620} = 0.07-0.2, 1.2 µL of culture growing exponentially in C+Y, pH 6.9 (in CO_2) was added to the C+Y, pH 7.1 agarose pads (normal atmosphere) as described before (10). Alternatively, cells were labeled for single molecule detections as described below and then added to agarose pads. After the added culture absorbed into the agarose pad (≤3 min), washed coverslips were placed on the pad. Sample slides were equilibrated to room temperature for 10 min, and then warmed to 37° C for at least 20 min, after which TIRFm was performed at 37° C. The microscope objective and stage were pre-heated to 37° C for 20 min prior to imaging.

TIRFm image acquisition. Most TIRFm imaging (≈60%) was performed using a DeltaVision OMX microscope (GE Healthcare) equipped for ring TIRF. Images were acquired with the Cascade II EM-CCD camera (Photometrics) and a 1.49NA Olympus

UApo N 100X oil objective (oil refractive index = 1.514). Some TIRFm imaging (≈40%) was performed on a new OMX-SR microscope, with an Olympus PlanApo N 60X/1.42 objective and an Olympus Apo N 60X/1.49 TIRF objective. Images were acquired using a PCO.edge 4.2 (CMOS) camera system (Kelheim Germany). Control comparisons showed that TIRFm and SM-TIRFm results were the same on the two microscopes. Brightfield microscopy images were often inverted when obtained with the new OMX-SR microscope resulting in cells with bright bodies that appear black or dark blue. The lasers used were: 488 nm (emission filter 500–550), and 561 nm (emission filter 609–654). Image acquisition was directed by OMX controller software (GE Healthcare). Images were taken at 1 s intervals for 45 ms exposure times for indicated times up to 540 s at the following transmission (T) settings: IU9985 (FtsZ-sfGFP native) 10% T; IU8845 (FtsZ-GFP) 10% T; IU10035 (GFP-FtsA) 10-50% T; IU14117 (EzrA-mNG) 50% T; IU13662 (FtsA'-sfGFP-FtsA') 100% T; IU10449 and IU10540 (EzrA-GFP) 10-50% T; IU9182 (GFP-MapZ) 100% T; IU9167 (DivIVA-GFP) 100% T; IU11005 (sfGFP-MltG) 100% T; IU11638 (GpsB-sfGFP) 100% T; IU9164 (GFP-StkP) 100% T; IU13910 (HT^{JF549}-bPBP2x) saturation and single-molecule labeling 100% T; IU14103 (HT^{JF549}-bPBP2x FtsZ-sfGFP) single-molecule labeling 100% T at 561 nm and 10% T at 488 nm; IU13315 (ectopic P_{Zn}-FtsZ-sfGFP induced with 0.1 mM $ZnCl_2$/0.01 mM $MnSO_4$) 50% T; IU14375 (ectopic P_{Zn}-FtsZ-sfGFP) induced with 0.1 mM $ZnCl_2$/0.01 mM $MnSO_4$) 50% T; IU13315 (ectopic P_{Zn}-FtsZ-sfGFP induced with 0.25 mM $ZnCl_2$/0.025 mM $MnSO_4$) 5% T; IU14288 (FtsZ-HT^{JF549}) saturation labeling 50% T; IU14288 (FtsZ-HT^{JF549}) single-molecule labeling 100% T ; IU14738 (iHT^{JF549}-MapZ) single-molecule labeling 100% T; IU14852 (ectopic P_{Zn}-FtsZ-(D214A)-sfGFP induced with 0.25 mM $ZnCl_2$/0.025 mM

$MnSO_4$) 5% T; IU14352 (FtsZ-HT^{JF549} EzrA-mNG) FtsZ single-molecule labeling 100% T at 561 nm and 50% T at 488nm); IU15096 (FtsW-HT^{JF549}) saturation and single-molecule labeling experiments 100% T; IU15599 (iHT^{JF549}-FtsZ) single-molecule labeling 100% T; IU15599 (iHT^{JF549}-FtsZ) saturation labeling 50% T; IU8918 (FtsW-GFP) 100% T; Images were acquired using the sequential mode.

Image processing for TIRFm. Alignment of channels was done using SoftWoRx software (GE Healthcare). Data were further processed and analyzed using FIJI (11). Rigid body registration using the StackReg plugin (12) was performed on images of cells expressing GFP fusions or HT fusions labeled with a high concentration (500 nM) of HT-JF549 ligand using the 488 nm or 561 nm channel, respectively. Summations were performed using FIJI, where brightness and contrast were adjusted to optimize signal associated with cells and minimize signal outside of cells. Kymographs were generated with the FIJI line tool at mature septal, nascent, and equatorial rings. The reslice function was then performed, and kymographs were generated containing the indicated number of images (at 1 frame/s) stacked vertically. The Angle function was drawn along the leading edge of movement paths until the leading edge disappeared or directional movement was no longer apparent and angle measurements were obtained. Only clear paths with directionality were analyzed to mitigate ambiguity. Movement paths were re-checked with videos to ensure the directionality of protein movements.

Periodicity analysis of FtsZ filaments/bundles in nascent rings. Appearance times were determined by calculating the time interval (s) between each start point of the leading edge of an FtsZ filament in a nascent or early equatorial ring and the start point of other leading edges of FtsZ filament moving in the same direction. The different

times between filament appearances included all combinations of FtsZ filaments that were tracked (i.e., four FtsZ-filaments traveling in the same direction have 6 possible combinations). Appearance times were graphed and binned in units of 9 s to generate a histogram. To simulate the mean and standard deviation of random events, random times were obtained from 1-180 s corresponding to the directional filament appearance number of events obtained for each experimental kymograph. For example, for each experimental kymograph displaying four filament appearance times, four random numbers were generated to produce six possible combinations from the randomized numbers. Randomization was performed 1,000 times per each experimental kymograph, resulting in a random mean and random standard deviation value for each appearance interval in the kymograph from 1-180 s.

Culture growth and sample preparation for microhole immobilization of *Spn* cells. FtsZ filament/bundle dynamics in cells vertically oriented in a microhole nano-device was determined as described previously (13) with the following modifications. Strain IU9985 was inoculated from a glycerol stock into 5 mL BHI and grown for 12-16 h at 37° C in 5 % CO_2 to early exponential phase (OD_{600} = 0.1-0.4). 2 mL of cells were centrifuged (16,500 × *g* for 5 min at 24° C). Pellets were washed once in 500 µL 1X PBS, and resuspended to OD_{600} = 0.02 in 2 mL C+Y, pH 7.8. Cells were grown to OD_{600} ≈ 0.2 at 37° C in 5 % CO_2 (pH 7.8 → pH 7.6), diluted to OD_{600} ≈ 0.1 in C+Y pH 7.6, and grown until they reached OD_{600} ≈ 0.2. Microhole patterned agarose gel pads were prepared using silicon micropillar arrays, as described by (13), using pads made from 6% (wt/vol) agarose gel in C+Y medium. Cells at OD_{600} ≈ 0.2 were centrifuged (1 mL, 16,500 × *g* for 5 min at 24° C), and resuspended in 10 µL C+Y, pH 7.6. 3-4 µL of cells

were loaded onto the agarose pad and a coverslip (plasma cleaned for 5 min) was mounted on top to seal the sample.

Imaging of vertically oriented cells in microholes. Cells immobilized in agarose microholes were imaged at 37.0° C on a Nikon N-SIM/N-STORM microscope, using a laser (488 nm) wide-field illumination, a 100x/1.49 TIRF Nikon objective, and an Andor iXon DU-897 camera (1 MHz conventional CCD mode), with 64 nm pixel size at the image plane. Time-lapses were acquired for 180 s, with 2 s exposure, and either 0.6 s (69 frames) or 0.34 s (77 frames) gaps between each frame.

Image processing and data analysis for cells in microholes. Images were denoised using the FIJI (11) plugin PureDenoise (14), with the following parameters: noise model set to automatic and individual, cycle-spins: 10 cycles, multiframe: 11 frames. Rigid body registration was further applied to the denoised images using the StackReg (12) FIJI plugin. Kymographs around the circumference of individual FtsZ-sfGFP rings (one pixel wide line) were extracted automatically using the custom MATLAB script ringDynamicsAnalysisWide.m (18). The straight line tool or the segmented line tool from FIJI was used to trace FtsZ-sfGFP filament/bundle tracks on the kymographs. A quality control step was used to assess the quality of the traced tracks. To perform that step the kymographs were filtered for clockwise and counter-clockwise motion using a FIJI macro adapted from the KymographClear macro tools (15). Tracks traced on the original kymographs were overlaid on the filtered kymographs, and those that did not match with the filtered tracks were discarded. The length in time and distance (around the circumference) of these tracks were further

measured to quantity the dynamics properties (speed, processivity and lifetime) of individual FtsZ-sfGFP treadmilling filaments/bundles.

Single-molecule (SM-) TIRFm. Cells expressing FtsZ-HT, HT-bPBP2x, iHT-MapZ, FtsW-HT, or iHT-FtsZ (where iHT refers to an i-tag sequence fused to the HaloTag (HT) domain; see SI Appendix, Table S1) were grown in 5 mL cultures of C+Y, pH 6.9 at 37° C in 5% CO_2 to early exponential phase (OD_{620} = 0.075-0.2). Limiting concentrations of 40 pM (FtsZ-HT or iHT-FtsZ) or 120 pM (all other HT- fusion proteins) HT-JF549 ligand was added to 500 μL of cultures, followed by brief vortex mixing. Cell mixtures were kept in the dark at 37° C for 15 min (normal atmosphere), after which they were collected by centrifugation (21,000 × *g*; 25° C; 5 min), and pellets were suspended in 400 μL of fresh C+Y, pH 7.1 by brief vortex mixing. The washing step was done one more time. Pellets were resuspended in 500 μL of C+Y, pH 7.1. Labeled cells (1.2 μL) were spotted on C+Y, pH 7.1 agarose pads, and TIRFm was performed as described above. For single FtsZ (Fig. 4 and SI Appendix, Fig. S12) or MapZ molecules (SI Appendix, Fig. S18), lifetimes of FtsZ or MapZ monomers were determined for signals that were present for 3 s (3 consecutive frames) or longer.

TIRFm of *ftsZ*(G107S)//P_{Zn}-*ftsZ-sfgfp* merodiploid strains (see SI Appendix, Fig. S15). Cells were inoculated from frozen glycerol stocks into BHI with added 0.25 mM $ZnCl_2$ and 0.025 mM $MnSO_4$ (+Zn), to mitigate selection of suppressor mutations, and cultures were grown for 12-16 h at 30° C in 5% CO_2 to early exponential phase (OD_{620} = 0.1-0.4). 2 mL of culture were centrifuged (21,000 × *g*; 25° C; 5 min), and pellets were washed once with C+Y, pH 7.1, suspended to OD_{620} ≈ 0.003 in 5 mL C+Y, pH 7.1 with added 0.1 mM $ZnCl_2$ and 0.01 mM $MnSO_4$ (+Zn) to induce limited expression of FtsZ-

sfGFP from the ectopic *bgaA* site. Cultures were grown at 37° C for 3.5 h in 5% CO_2, at which time samples were placed on agarose pads containing C+Y, pH 7.1 with added 0.1 mM $ZnCl_2$ and 0.01 mM $MnSO_4$ (+Zn) and observed by 2D-epifluorescence microscopy and TIRFm as described above.

TIRFm of HT-bPBP2x in an *ftsZ*(G107S) mutant (see SI Appendix, Fig. S22). Strain IU14508 was inoculated from frozen glycerol stocks into BHI with added 0.25 mM $ZnCl_2$ and 0.025 mM $MnSO_4$ (+Zn), and cultures were grown for 12-16 h at 30° C in 5% CO_2 to early exponential phase (OD_{620} = 0.1-0.4). 2 mL of culture were centrifuged (21,000 × *g*; 25° C; 5 min), and pellets were washed once with C+Y, pH 7.1, suspended to OD_{620} ≈ 0.005 in 5 mL C+Y, pH 7.1. At ≈6.5 h into growth at 30° C, cultures were switched to growth at 37° C and growth curve analysis was performed to ensure strains showed a temperature-sensitive phenotype (lower growth yield and cell lysis). For TIRFm, 500 µL of culture of IU13910 and IU14508 growing exponentially at 30° C were removed and labeled with 120 pM HT-JF549 ligand for 20 min at 30° C. The labeled strains were washed once with 1 mL fresh C+Y, pH 7.1, centrifuged (21,000 × *g*; 25° C; 5 min), and resuspended in 200 µL fresh C+Y, pH 7.1, and 1.2 µL was spotted onto agarose pads. Prepared TIRFm samples were allowed to equilibrate to 37° C for at least 30 min prior to imaging. TIRFm was performed and velocities of directionally moving single molecules of bPBP2x were determined as described above.

TIRFm of P_{Zn}-*ftsZ*(D214A) or P_{Zn}-*ftsZ*(D214A)-*sfgfp* merodiploid strains. Cells were inoculated from frozen glycerol stocks into BHI, and cultures were grown for 12-16 h at 37° C in 5% CO_2 to early exponential phase (OD_{620} ≈ 0.1-0.4). 2 mL of culture were centrifuged (21,000×*g*; 25° C; 5 min), and pellets were washed once with C+Y, pH 7.1,

suspended in 5 mL C+Y to OD_{620} ≈ 0.003, pH 7.1 with added 0.25 mM $ZnCl_2$ and 0.025 mM $MnSO_4$ (+Zn) to induce expression of FtsZ(D214A) or FtsZ(D214A)-sfGFP from the ectopic *bgaA* site. Cultures were grown at 37° C for 3.5 h in 5% CO_2, at which time samples were added to agarose pads containing C+Y, pH 7.1 with added 0.25 mM $ZnCl_2$ and 0.025 mM $MnSO_4$ (+Zn) and observed by 2D- epifluorescence microscopy or TIRFm as described above. For single molecule labeling of FtsW-HT and HT-bPBP2x, 500 µL of culture were labeled with 120 pM HT-JF549 ligand in C+Y pH 7.1 with added 0.25 mM Zn and 0.025 mM $MnSO_4$ (+Zn), centrifuged (21,000 × *g*; 25° C; 5 min), and washed once with C+Y pH 7.1, resuspended in 300 µL, and spotted on agarose pads containing C+Y, pH 7.1 with added 0.25 mM Zn and 0.025 mM $MnSO_4$ (+Zn) for TIRFm.

3D-SIM immunofluorescence microscopy (IFM). IFM was performed as described previously (16) on strains growing exponentially in BHI broth at 37° C in 5% CO_2. The following antibody combinations and conditions were used: strain IU9207 (primary antibodies: rabbit anti-HA and mouse anti-FLAG at 37° C for 2 h; secondary antibodies: 488 anti-rabbit and 568 anti-mouse at 24° C for 1 h); and strain IU9090 (primary antibodies; rabbit anti-FLAG and mouse anti-Myc at 24° C for 1 h; secondary antibodies; 488 anti-rabbit and 568 anti-mouse at 24° C for 1 h). Control experiments were performed as described for strain IU10304 in (17). 3D-SIM was performed using the OMX 3D-SIM super resolution system located in the Indiana University Bloomington Light Microscopy Center (LMIC). Exposure times and %T (transmission) settings for DAPI, Alexa-488, and Alexa 568 images were 10-100 ms and 50%, 50 ms and 1-10%, and 50 ms and 10-50%, respectively.

Co-immunoprecipitation (co-IP) of FtsW-GFP with bPBP2x-FLAG. Co-IP was adapted from (6) with modifications. Cultures were grown exponentially in 400 mL of BHI with 5% CO_2 to OD_{620} = 0.25-0.40. Cells were collected by centrifugation (8,000×*g* for 10 min at 4° C). Cell pellets were washed once with 30 mL of 1X PBS (4° C) and resuspended in 19.2 mL 1X PBS (24° C). 800 µL of 10% paraformaldehyde solution (EMS) were added for crosslinking to a final concentration of 0.4% (vol/vol). Mixtures were incubated for 30 min at 24° C. Cross-linking reactions were quenched by the addition of 4 mL 1.0 M glycine followed by incubation at 24° C for 10 min. Cells were collected by centrifugation (16,500× *g* for 5 min at 4° C). Pellets were washed once with 20 mL cold 1X PBS (4° C) and resuspended in 2 mL of cold lysis buffer (50 mM Tris-HCl pH 7.4, 150 mM NaCl, 1 mM EDTA, 1% (vol/vol) Triton X100) with 1 tablet of protease inhibitor (ThermoFisher Scientific, 78429) freshly added per 10 mL of lysis buffer. The suspension was transferred into 2 lysing matrix B tubes (MP Biomedicals) with 1 mL in each tube. Tubes were shaken in FastPrep homogenizer 9X (3X; 5 min on ice; 3X; 5 min on ice; and 3X) with 6.0 M/s for 40 s each at 4° C. Cell debris and lysing matrix from tubes were removed by centrifugation at 16,000 × *g* for 5 min at 4° C. The protein concentration of each sample was determined by Bio-Rad DC™ protein assay (Bio-Rad). 1 mL of lysate with equal amounts of total protein (5 mg/mL) was added to tubes with 50 µL of anti-FLAG magnetic beads (Sigma, M8823). The same amount of protein was loaded onto the beads for strains expressing bPBP2x-FLAG and the corresponding control strains non-tagged wild-type bPBP2x in each experiment. The tubes were rotated for 2 h at 4° C. The beads were washed 3 times with 1 mL of lysis buffer (4° C) with 10 min incubation at 4° C each time. FLAG-tagged protein was eluted

from the beads by incubation with 100 µL of FLAG elution solution (150 ng 3X FLAG peptide/µL) (Sigma, F4799) for 30 min at 4° C. 100 µL of the elution and the original lysate that was added to the magnetic beads (input) were mixed with 100 µL 2× Laemmli sample buffer (Bio-Rad) containing 5% (vol/vol) β-mercaptoethanol (Sigma). 50 µg of input protein samples and 20 µL of each elution sample were separated by SDS-PAGE on 4-15% precast protein gels (Bio-Rad) in Tris-glycine buffer. Gels were Western blotted using rabbit anti-FLAG (1:2000) or rabbit anti-GFP (1:1000) as primary antibody. Secondary antibody used was ECL anti-rabbit IgG, horseradish peroxidase linked whole antibody (1:10,000). The experiment was performed twice with similar results.

Labeling of FtsZ(GTPase) mutant cells with FDAAs. Labeling of cells was adapted from (6) with modifications. For Fig. 8.B.I, cultures were grown in BHI supplemented with 0.25 mM $ZnCl_2$ and 0.025 mM $MnSO_4$ at 30° C to OD_{620} = 0.1-0.4), washed once with C+Y pH 7.1, and resuspended in C+Y pH 7.1 (no additional Zn/Mn) to $OD_{620} \approx 0.003$ at 37° C. At OD_{620} = 0.1-0.2, 500 µL of culture were labeled with FDAA (2.5 min at 125 µM final TADA in C+Y pH 7.1 at 37° C). Following labeling of cells, cells were placed on ice for 10-30 s, and collected by centrifugation (16,500 × *g* for 2.5 min at 4° C), washed twice with centrifugation with 1 mL cold 1xPBS (16,500 × *g* for 2.5 min at 4° C), and fixed with 4% paraformaldehyde as described before (6). After fixation, cells were washed in cold 1xPBS 2 additional times. Cells were resuspended in 50 µL GTE buffer and imaged. Central microscopic fields of a comparable numbers of cells were analyzed to minimize uneven imaging and autofluorescence. For Fig 8.B.II, cultures were grown in BHI (not supplemented with Zn/Mn) at 37° C in 5% CO_2 to OD_{620} = 0.1-

0.4), washed once with C+Y pH 7.1, and resuspended in C+Y pH 7.1 with added 0.25 mM $ZnCl_2$ and 0.025 mM $MnSO_4$ (+Zn) to OD_{620} ≈ 0.003 at 37° C in 5% CO_2. At OD_{620} = 0.1-0.2, 500 µL of culture were labeled with FDAA (2.5 min at 125 µM final TADA in C+Y, pH 7.1 at 37°C). Following labeling, cells were washed, fixed, and imaged as described above. Mean FDAA fluorescent intensity (AU) per cell was determined by using MicrobeJ (9) to pick cells based on cell shape in phase-contrast images, as described for 2D-epifluorescence microscopy and demograph generation, above. Following cell selection, the statistics function was used to determine the mean intensity of FDAA fluorescence intensity (AU) per cell. Individual averages were then used to determine the values shown in Figure 8B.

TIRFm of methicillin treated cells. Cells were spotted onto agarose pads containing methicillin on slides that were assembled as usual with the following changes. After 45 min to allow agarose solidification, the top slide was gently removed from the apparatus and 9 µL of freshly made 1 µg/mL methicillin (in C+Y pH 7.1) was spotted on agarose (mass of agarose pad was ≈ 30 mg) to a final concentration of ≈ 0.3 µg mL^{-1} methicillin. The apparatus containing the slide and the agarose pad with methicillin, was then put in a pre-warmed 37° C moist chamber with 5% CO_2 to allow the equilibration of the antibiotic into the agarose for 10 min. Following equilibration, the agarose pad was allowed adjust to room temperature for 5 min to assist in drying of excess liquid, after which the slide with the agarose pad containing methicillin was prepared for TIRFm by cutting one third of the center of agarose pad out using a clean razor blade, followed by addition of 1.2 µL cells (pre-labeled cells for IU13910), appropriate drying, and coverslip placement on top of the agarose pad. Prior to imaging,

the cells were allowed to grow on the agarose pad containing methicillin for 45 min at 37° C, after which TIRFm was performed for 30 min.

References cited in SI Appendix, Experimental Procedures

1. Lanie JA*, et al.* (2007) Genome sequence of Avery's virulent serotype 2 strain D39 of *Streptococcus pneumoniae* and comparison with that of unencapsulated laboratory strain R6. *J Bacteriol* 189(1):38-51.
2. Tsui HC*, et al.* (2010) Identification and characterization of noncoding small RNAs in *Streptococcus pneumoniae* serotype 2 strain D39. *J Bacteriol* 192(1):264-279.
3. Land AD*, et al.* (2013) Requirement of essential Pbp2x and GpsB for septal ring closure in *Streptococcus pneumoniae* D39. *Mol Microbiol* 90(5):939-955.
4. van Raaphorst R, Kjos M, & Veening JW (2017) Chromosome segregation drives division site selection in *Streptococcus pneumoniae*. *Proc Natl Acad of Sci USA* 114(29):E5959-E5968.
5. Martin B, Garcia P, Castanie MP, & Claverys JP (1995) The *recA* gene of *Streptococcus pneumoniae* is part of a competence-induced operon and controls lysogenic induction. *Mol Microbiol* 15(2):367-379.
6. Rued BE*, et al.* (2017) Suppression and synthetic-lethal genetic relationships of Δ*gpsB* mutations indicate that GpsB mediates protein phosphorylation and penicillin binding protein interactions in *Streptococcus pneumoniae* D39. *Mol Microbiol* 103(6):931-957.
7. Lara B*, et al.* (2005) Cell division in cocci: localization and properties of the *Streptococcus pneumoniae* FtsA protein. *Mol Microbiol* 55(3):699-711.
8. Maurer P*, et al.* (2008) Penicillin-binding protein 2x of *Streptococcus pneumoniae*: three new mutational pathways for remodelling an essential enzyme into a resistance determinant. *J Mol Biol* 376(5):1403-1416.
9. Ducret A, Quardokus EM, & Brun YV (2016) MicrobeJ, a tool for high throughput bacterial cell detection and quantitative analysis. *Nat microbiol* 1(7):16077.
10. de Jong IG, Beilharz K, Kuipers OP, & Veening JW (2011) Live Cell Imaging of *Bacillus subtilis* and *Streptococcus pneumoniae* using Automated Time-lapse Microscopy. *J Vis Exp: JoVE* (53) pii: 3145.
11. Schindelin J*, et al.* (2012) Fiji: an open-source platform for biological-image analysis. *Nat Methods* 9(7):676-682.
12. Thevenaz P, Ruttimann UE, & Unser M (1998) A pyramid approach to subpixel registration based on intensity. *IEEE Trans Image Process* 7(1):27-41.
13. Bisson-Filho AW*, et al.* (2017) Treadmilling by FtsZ filaments drives peptidoglycan synthesis and bacterial cell division. *Science* 355(6326):739-743.
14. Luisier F, Vonesch C, Blu T, & Unser M (2010) Fast interscale wavelet denoising of Poisson-corrupted images. *Sign Process* 90(2):415-427.
15. Mangeol P, Prevo B, & Peterman EJ (2016) KymographClear and KymographDirect: two tools for the automated quantitative analysis of molecular and cellular dynamics using kymographs. *Mol Bio Cell* 27(12):1948-1957.
16. Tsui HC*, et al.* (2014) Pbp2x localizes separately from Pbp2b and other peptidoglycan synthesis proteins during later stages of cell division of *Streptococcus*

pneumoniae D39. *Mol Microbiol* 94(1):21-40.

17. Mura A, *et al.* (2016) Roles of the essential protein FtsA in cell growth and division in *Streptococcus pneumoniae. J Bacteriol 199*(3), e00608-16.
18. Ring-fitting-bisson2017: Microhole ring fitting (circular kymograph analysis) code associated with Bisson-Filho *et al*. Science 2017 (2018) (HoldenLab) Available at: https://github.com/HoldenLab/ring-fitting-bisson2017 [Accessed August 14, 2018].

SI APPENDIX, TABLE S1. Bacterial strains and oligonucleotides used in this study

***S. pneumoniae* (*Spn*) strains**			
Strain number	Genotype (description)[a b c d l]	Antibi-otic resist-ance	Reference or source
IU1824	D39 Δ*cps rpsL1*	Str^R	(1)
IU1945	D39 Δ*cps*	None	(1)
IU5456	D39 Δ*cps ezrA*-L$_0$-FLAG^3-P$_c$-*erm*	Erm^R	(2)
IU5544	D39 Δ*cps gpsB*-L$_0$-FLAG^3-P$_c$-*erm*	Erm^R	(11)
IU5648	D39 Δ*cps rpsL1 divIVA*$^+$-P$_c$-[*kan-rpsL*$^+$] (IU1824 X *divIVA*$^+$-P$_c$-[*kan-rpsL*$^+$] amplicon from PCR fusion)	Kan^R	This Study
IU6506	D39 Δ*cps pbp2x*-L$_0$-FLAG^3-P$_c$-*erm* (IU1945 X *pbp2x*-L$_0$-FLAG^3-P$_c$-*erm* amplicon from PCR fusion)	Erm^R	This Study
IU6541	D39 Δ*cps pbp2x*-FLAG-P$_c$-*erm* (IU1945 X *pbp2x*-FLAG-P$_c$-*erm* amplicon from PCR fusion)	Erm^R	This Study
IU6810	D39 Δ*cps ezrA*-HA-P$_c$-*kan*	Kan^R	(2)
IU7054	D39 Δ*cps ftsZ*$^+$//*bgaA'*::*kan-t1t2*-P$_{ftsA}$-*ftsZ*$^+$ (IU1945 X *bgaA*::*kan*-t1t2-P$_{ftsA}$-*ftsZ*$^+$ amplicon from PCR fusion)	Kan^R	This Study
IU7506	D39 Δ*cps rpsL1* Δ*pbp2x*<>*aad9*//*CEP*:: P$_{fcsK}$-*pbp2x*$^+$	Spc^R Str^R	(18)
IU7614	D39 Δ*cps rpsL1 ftsZ*$^+$-P$_c$-[*kan-rpsL*$^+$]	Kan^R	(3)
IU7616	D39 Δ*cps rpsL1 ftsA*$^+$-P$_c$-[*kan-rpsL*$^+$] (IU1824 X *ftsA*$^+$-P$_c$-[*kan-rpsL*$^+$] amplicon from PCR fusion)	Kan^R	This Study
IU7667	D39 Δ*cps rpsL1 ftsZ*-Myc	Str^R	(4)
IU7673	D39 Δ*cps rpsL1* P$_c$-[*kan-rpsL*$^+$]-*stkP*$^+$	Kan^R	(2)
IU7814	D39 Δ*cps* Δ*ftsZ*::*aad9*//*bgaA'*::*kan*-t1t2-P$_{ftsA}$-*ftsZ*$^+$ (IU7054 X Δ*ftsZ*::*aad9* amplicon from PCR fusion)	Kan^R Spc^R	This Study
IU8122	D39 Δ*cps ftsZ*$^+$//*bgaA'*::*tet*-P$_{Zn}$-*ftsZ*$^+$ (IU1945 X *bgaA'*::*tet*-P$_{Zn}$-*ftsZ*$^+$ amplicon from PCR fusion)	Tet^R	This Study
IU8124[f]	D39 Δ*cps* Δ*ftsZ*::*aad9*//*bgaA'*::*tet*-P$_{Zn}$-*ftsZ*$^+$ (IU7814 X amplicon, *bgaA'*::*tet*-P$_{Zn}$-*ftsZ*$^+$ from IU8122)	Spc^R Tet^R	This Study
IU8845	D39 Δ*cps rpsL1 ftsZ*-L$_2$-*sfgfp* (IU7614 X *ftsZ*-L$_2$-*gfp* amplicon from PCR fusion)	Str^R	This Study
IU8900	D39 Δ*cps rpsL1 ftsW*$^+$-P$_c$-[*kan-rpsL*$^+$] (IU1824 X *ftsW*$^+$-P$_c$-[*kan-rpsL*$^+$] amplicon from PCR fusion)	Kan^R	This Study
IU8980	D39 Δ*cps rpsL1* P$_c$-[*kan-rpsL*$^+$]-*mltG*$^+$	Kan^R	(3)
IU8918	D39 Δ*cps rpsL1 ftsW*-L$_2$-*gfp* (IU1824 X *ftsW*-L$_2$-*gfp* amplicon from PCR fusion)	Str^R	This Study
IU8921	D39 Δ*cps rpsL1* P$_c$-[*kan-rpsL*$^+$]-*pbp2x*$^+$ (IU1824 X P$_c$-[*kan-rpsL*$^+$]-*pbp2x*$^+$ from PCR fusion)	Kan^R	This Study

IU9020	D39 Δ*cps rpsL1 gfp*-L_1-*pbp2x* (IU8921 X *gfp*-L_1-*pbp2x* amplicon from PCR fusion)	Str^R	This Study
IU9086	D39 Δ*cps rpsL1* Δ*mapZ*::P_c-[*kan-rpsL*$^+$] (IU1824 X Δ*mapZ*::P_c-[*kan-rpsL*$^+$] amplicon from PCR fusion)	Kan^R	This Study
IU9023	D39 Δ*cps rpsL1* P_c-[*kan-rpsL*$^+$]-*pbp2b*$^+$ (IU1824 X P_c-[*kan-rpsL*$^+$]-*pbp2b*$^+$ from PCR fusion)	Kan^R	This Study
IU9094	D39 Δ*cps rpsL1* P_c-[*kan-rpsL*$^+$]-*mapZ*$^+$ (IU1824 X P_c-[*kan-rpsL*$^+$]-*mapZ*$^+$ amplicon from PCR fusion)	Kan^R	This Study
IU9077	D39 Δ*cps rpsL1 ezrA*$^+$-P_c-[*kan-rpsL*$^+$] (IU1824 X *ezrA*$^+$-P_c-[*kan-rpsL*$^+$] amplicon from PCR fusion)	Kan^R	This Study
IU9090	D39 Δ*cps rpsL1 ftsZ*-Myc *mapZ*-L_0-FLAG^3-P_c-*erm* (IU7667 X *mapZ*-L_0-FLAG^3-P_c-*erm* amplicon from PCR fusion)	Erm^R Str^R	This Study
IU9097	D39 Δ*cps rpsL1* Δ*mapZ*::P_c-[*kan-rpsL*$^+$] *ftsZ*-L_2-*gfp* (IU8845 X Δ*mapZ*::P_c-[*kan-rpsL*$^+$] amplicon from IU9086)	Kan^R	This Study
IU9164	D39 Δ*cps rpsL1 gfp*-L_1-*stkP* (IU7673 X *gfp*-L_1-*stkP* amplicon from PCR fusion)	Str^R	This Study
IU9167	D39 Δ*cps rpsL1 divIVA*-L_2-*gfp* (IU5648 X *divIVA*-L_1-*gfp* amplicon from PCR fusion)	Str^R	This Study
IU9175	D39 Δ*cps rpsL1* Δ*mapZ*	Str^R	(17)
IU9182	D39 Δ*cps rpsL1 gfp*-L_1-*mapZ* (IU9094 X *gfp*-L_1-*mapZ* amplicon from PCR fusion)	Str^R	This Study
IU9207	D39 Δ*cps ezrA*-HA-P_c-*kan mapZ*-L_0-FLAG^3-P_c-*erm* (IU6810 X *mapZ*-L_0-FLAG^3-P_c-*erm* amplicon from IU9090)	Kan^R Erm^R	This Study
IU9683	D39 Δ*cps hlpA-sfgfp-Cm* (IU1945 transformed with *hlpA-sfgfp-Cm* amplicon from JWV500 from Veening Lab)	Cm^R	This Study
IU9767	D39 Δ*cps rpsL1* P_c-[*kan-rpsL*$^+$]-*ftsA*$^+$	Kan^R	(4)
IU9881	D39 Δ*cps rpsL1* Δ*mapZ ftsZ*-L_2-*gfp* (IU9097 X Δ*mapZ* amplicon from IU9175)	Str^R	This Study
IU9965	D39 Δ*cps rpsL1 sfgfp*-L_1-*pbp2b* (IU9023 X *sfgfp*-L_1-*pbp2b* amplicon from PCR fusion)	Str^R	This Study
IU9985	D39 Δ*cps rpsL1 ftsZ*-L_2-*sfgfp* (IU7614 X *ftsZ*-L_2-*sfgfp* amplicon from PCR fusion)	Str^R	This Study
IU10035	D39 Δ*cps rpsL1 gfp*-L_1-*ftsA* (IU9767 X *gfp*-L_1-*ftsA* from PCR fusion)	Str^R	This Study
IU10063	D39 Δ*cps pbp2x*$^+$//*bgaA::tet*-P_{Zn}-*pbp2x*$^+$ (IU1945 X *bgaA::tet*-P_{Zn}-*pbp2x*$^+$ from PCR fusion)	Tet^R	This Study
IU10228	D39 Δ*cps rpsL1 gfp*-L_1-*mltG*	Str^R	(3)
IU10254	D39 Δ*cps rpsL1 ezrA*-L_0-*sfgfp* (IU9077 X *ezrA*-L_0-*sfgfp* amplicon from PCR fusion)	Str^R	This Study
IU10294	D39 Δ*cps rpsL1 Δpbp1a* Δ*mltG*::P_c-*erm* Δ*spd_1874*::P_c-[*kan-rpsL*$^+$] Δ*spd_0104*::P_c-*cat*	Kan^R Erm^R Str^R	(3)

IU10449	D39 Δ*cps rpsL1 ezrA*-L_0-*gfp* (IU9077 X *ezrA*-L_0-*gfp* amplicon from PCR fusion)	Str^R	This Study
IU10526	D39 Δ*cps rpsL1 ezrA*-L_0-*gfp* Δ*mapZ*::P_c-[*kan-rpsL*$^+$] (IU10449 was transformed with Δ*mapZ*::P_c-[*kan-rpsL*$^+$] amplicon from IU9086)	Kan^R	This Study
IU10540	D39 Δ*cps rpsL1 ezrA*-L_0-*gfp* Δ*mapZ* (IU10526 was transformed with Δ*mapZ* amplicon from IU9175)	Str^R	This Study
IU10612[g]	D39 Δ*cps rpsL1 ftsZ*(G107S)//*bgaA::tet*-P_{Zn}-*ftsZ*$^+$ (IU8124 X *ftsZ*(G107S) amplicon from PCR fusion)	Str^R Tet^R	This Study
IU11005	D39 Δ*cps rpsL1 sfgfp*-L_1-*mltG* (IU8980 X *sfgfp*-L_1-*mltG* amplicon from PCR fusion)	Str^R	This Study
IU11119	D39 Δ*cps ezrA*-L_0-*sfgfp*-P_c-*cat* (IU1945 X *ezrA*-L_0-*sfgfp*-P_c-*cat* amplicon from PCR fusion)	Cm^R	This Study
IU11157	D39 Δ*cps rpsL1 isfgfp*-L_1-*pbp2x* (IU8921 X *isfgfp*-L_1-*pbp2x* amplicon from PCR fusion)	Str^R	This Study
IU11594	D39 Δ*cps gpsB*-L_0-*sfgfp*-P_c-*cat* (IU1945 X *gpsB*-L_0-*sfgfp*-P_c-*cat* amplicon from PCR fusion)	Cm^R	This Study
IU11638	D39 Δ*cps rpsL1 gpsB*-L_0-*sfgfp*-P_c-*cat* (IU1824 X *gpsB*-L_0-*sfgfp*-P_c-*cat* amplicon from IU11594)	Cm^R Str^R	This Study
IU12286	D39 Δ*cps rpsL1 ftsZ*$^+$//*bgaA*'::*tet*-P_{Zn}-*ftsZ*$^+$ (IU1824 X *bgaA*'::*tet*-P_{Zn}-*ftsZ*$^+$ amplicon from IU8122)	Str^R Tet^R	This Study
IU12406[f]	D39 Δ*cps rpsL1* Δ*ftsZ*::*aad9*//*bgaA*'::*tet*-P_{Zn}-*ftsZ*$^+$ (IU12286 X Δ*ftsZ*::*aad9* amplicon from IU7814)	Spc^R Str^R Tet^R	This Study
IU13315	D39 Δ*cps rpsL1 ftsZ*$^+$//*bgaA::tet*-P_{Zn}-*ftsZ*-L_2-*sfgfp* (IU1824 X *bgaA::tet*-P_{Zn}-*ftsZ*-L_2-*sfgfp* amplicon from Gibson Fusion)	Str^R Tet^R	This Study
IU13396	D39 Δ*cps rpsL1* Δ*murA1*::P_c-[*kan-rpsL*$^+$] (IU1824 X Δ*murA1*::P_c-[*kan-rpsL*$^+$] amplicon from K767)	Kan^R	This Study
IU13406	D39 Δ*cps rpsL1 ftsZ*-L_5-*cfp-erm* (IU1824 X *ftsZ*-L_5-*cfp-erm* amplicon from MK458[e])	Erm^R Str^R	This Study
IU13408	D39 Δ*cps rpsL1* Δ*mapZ ftsZ*-L_5-*cfp-erm* (IU9175 X *ftsZ*-L_5-*cfp-erm* amplicon from MK458[e])	Erm^R Str^R	This Study
IU13536	D39 Δ*cps rpsL1* Δ*murA1* (IU13396 X Δ*murA1* amplicon from PCR fusion)	Str^R	This Study
IU13662	D39 Δ*cps rpsL1 ftsA'*-L_8-*sfgfp*-L_8-*ftsA'* (IU7616 X *ftsA'*-L_8-*sfgfp*-L_8-*ftsA'* from PCR fusion)	Str^R	This Study
IU13910	D39 Δ*cps rpsL1 ht*-L_6-*pbp2x* (IU8921 X *ht*-L_6-*pbp2x* amplicon from PCR fusion)	Str^R	This Study
IU13969	D39 Δ*cps rpsL1* P_c-[*kan-rpsL*$^+$]-*pbp2x*$^+$//*bgaA::tet*-P_{Zn}-*pbp2x*$^+$ (IU8921 X *bgaA::tet*-P_{Zn}-*pbp2x*$^+$ from IU10063)	Kan^R Tet^R	This Study
IU14032	D39 Δ*cps rpsL1* P_c-[*kan-rpsL*$^+$]-*pbp2x*$^+$ *ftsZ*-L_2-*sfgfp* (IU9985 X P_c-[*kan-rpsL*$^+$]-*pbp2x*$^+$ from	Kan^R	This Study

	IU8921)		
IU14059[m]	D39 Δ*cps rpsL1* Δ*pbp2x*<>*aad9*//*bgaA*::*tet*-P_{Zn}-*pbp2x*$^+$ (IU13969 X Δ*pbp2x*<>*aad9* from IU7506)	Spc^R Tet^R	This Study
IU14103	D39 Δ*cps rpsL1 ht*-L_6-*pbp2x ftsZ*-L_2-*sfgfp* (IU14032 X *ht*-L_6-*pbp2x* from IU13910)	Str^R	This Study
IU14117	D39 Δ*cps rpsL1 ezrA*-L_0-*mneongreen*-P_c-*cat* (IU1824 X *ezrA*-L_0-*mneongreen*-P_c-*cat* from PCR fusion)	Cm^R Str^R	This Study
IU14288	D39 Δ*cps rpsL1 ftsZ*-L_5-*ht*-*erm* (IU1824 X *ftsZ*-L_5-*ht*-*erm* from PCR fusion)	Erm^R Str^R	This Study
IU14290	D39 Δ*cps rpsL1 pbp1a*-L_0-*ht*-P_c-*erm* (IU1824 X *pbp1a*-L_0-*ht*-P_c-*erm* from PCR fusion)	Erm^R Str^R	This Study
IU14330	D39 Δ*cps rpsL1 pbp2x*$^+$//*bgaA*::*tet*-P_{Zn}-*pbp2x*$^+$ (IU1824 X *bgaA*::*tet*-P_{Zn}-*pbp2x*$^+$ from IU10063)	Str^R Tet^R	This Study
IU14352	D39 Δ*cps rpsL1 ftsZ*-L_5-*ht*-*erm ezrA*-L_0-*mneongreen*-P_c-*cat* (IU14288 X *ezrA*-L_0-*mneongreen*-P_c-*cat* from IU14117)	Cm^R Erm^R Str^R	This Study
IU14355[f]	D39 Δ*cps rpsL1* Δ*ftsZ*::*aad9*//*bgaA*::*tet*-P_{Zn}-*ftsZ*-L_2-*sfgfp* (IU13315 X Δ*ftsZ*::*aad9* amplicon from IU7814)	Spc^R Str^R Tet^R	This Study
IU14375[g]	D39 Δ*cps rpsL1 ftsZ*(G107S)//*bgaA*::*tet*-P_{Zn}-*ftsZ*-L_2-*sfgfp* (IU14355 X *ftsZ*(G107S) amplicon from IU10612)	Str^R Tet^R	This Study
IU14377[g]	D39 Δ*cps rpsL1 ftsZ*(G107S)//*bgaA*::*tet*-P_{Zn}-*ftsZ*$^+$ (IU12406 X *ftsZ*(G107S) amplicon from IU10612)	Str^R Tet^R	This Study
IU14404	D39 Δ*cps rpsL1 ezrA*-L_0-*ht*-P_c-*erm* (IU1824 X *ezrA*-L_0-*ht*-P_c-*erm* from PCR fusion)	Erm^R Str^R	This Study
IU14408[g]	D39 Δ*cps rpsL1* P_c-[*kan-rpsL*$^+$]-*pbp2x*$^+$ *ftsZ*(G107S)//*bgaA*::*tet*-P_{Zn}-*ftsZ*$^+$ (IU14377 X P_c-[*kan-rpsL*$^+$]-*pbp2x*$^+$ from IU8921)	Kan^R Tet^R	This Study
IU14508[g]	D39 Δ*cps rpsL1 ht*-L_6-*pbp2x* *ftsZ*(G107S)//*bgaA*::*tet*-P_{Zn}-*ftsZ*$^+$ (IU14408 X *ht*-L_6-*pbp2x* from IU13910)	Str^R Tet^R	This Study
IU14662	D39 Δ*cps rpsL1 ht*-L_6-*mapZ* (IU9094 X *ht*-L_6-*mapZ* amplicon from PCR fusion)	Str^R	This Study
IU14714	D39 Δ*cps rpsL1 sfgfp*-L_1-*pbp2b* P_c-[*kan-rpsL*$^+$]-*pbp2x*$^+$ (IU9965 X P_c-[*kan-rpsL*$^+$]-*pbp2x*$^+$ from IU8921)	Kan^R	This study
IU14738[l]	D39 Δ*cps rpsL1 iht*-L_6-*mapZ* (IU9094 X *iht*-L_6-*mapZ* amplicon from PCR fusion)	Str^R	This Study
IU14850	D39 Δ*cps rpsL1 ftsZ*$^+$//*bgaA*'::*tet*-P_{Zn}-*ftsZ*(D214A) (IU1824 X *bgaA*'::*tet*-P_{Zn}-*ftsZ*(D214A) amplicon from PCR fusion)	Str^R Tet^R	This study
IU14852	D39 Δ*cps rpsL1 ftsZ*$^+$//*bgaA*'::*tet*-P_{Zn}-*ftsZ*(D214A)-L_2-*sfgfp* (IU1824 X *bgaA*'::*tet*-P_{Zn}-*ftsZ*(D214A)-L_2-	Str^R Tet^R	This study

	sfgfp amplicon from PCR fusion)		
IU14927	D39 Δ*cps rpsL1 iht*-L_6-*pbp2x* (IU8921 X *iht*-L_6-*pbp2x* amplicon from PCR fusion)	Str^R	This Study
IU14931	D39 Δ*cps rpsL1 ftsW*-L_2-*gfp* P_c-[*kan-rpsL*$^+$]-*pbp2x*$^+$ (IU8918 X P_c-[*kan-rpsL*$^+$]-*pbp2x*$^+$ from IU8921)	Kan^R	This study
IU14964	D39 Δ*cps rpsL1 ftsW*-L_2-*gfp pbp2x*-FLAG-P_c-*erm* (IU8918 X *pbp2x*-FLAG-P_c-*erm* amplicon from IU6541)	Erm^R Str^R	This Study
IU15032	D39 Δ*cps rpsL1* P_c-[*kan-rpsL*$^+$]-*pbp2x*$^+$ *ftsZ*$^+$//*bgaA*'::*tet*-P_{Zn}-*ftsZ*$^+$ (IU12286 X P_c-[*kan-rpsL*$^+$]-*pbp2x*$^+$ amplicon from IU8921)	Kan^R Tet^R	This study
IU15033	D39 Δ*cps rpsL1* P_c-[*kan-rpsL*$^+$]-*pbp2x*$^+$ Δ*murA1* (IU13536 X P_c-[*kan-rpsL*$^+$]-*pbp2x*$^+$ amplicon from IU8921)	Kan^R	This study
IU15035	D39 Δ*cps rpsL1* P_c-[*kan-rpsL*$^+$]-*pbp2x*$^+$ *ftsZ*$^+$//*bgaA*'::*tet*-P_{Zn}-*ftsZ*(D214A) (IU14850 X P_c-[*kan-rpsL*$^+$]-*pbp2x*$^+$ amplicon from IU8921)	Kan^R Tet^R	This study
IU15038	D39 Δ*cps rpsL1 ht*-L_6-*pbp2x ftsZ*$^+$//*bgaA*'::*tet*-P_{Zn}-*ftsZ*$^+$ (IU15032 X *ht*-L_6-*pbp2x* from IU13910)	Str^R Tet^R	This study
IU15039	D39 Δ*cps rpsL1 ht*-L_6-*pbp2x* Δ*murA1* (IU15033 X *ht*-L_6-*pbp2x* from IU13910)	Str^R	This study
IU15041	D39 Δ*cps rpsL1 ht*-L_6-*pbp2x ftsZ*$^+$//*bgaA*'::*tet*-P_{Zn}-*ftsZ*(D214A) (IU15035 X *ht*-L_6-*pbp2x* from IU13910)	Str^R Tet^R	This study
IU15066	D39 Δ*cps rpsL1 iht*-L_6-*pbp2x ftsW*-L_2-*gfp* (IU14931 X *iht*-L_6-*pbp2x* amplicon from IU14927)	Str^R	This Study
IU15068	D39 Δ*cps rpsL1 iht*-L_6-*pbp2x sfgfp*-L_1-*pbp2b* (IU14714 X *iht*-L_6-*pbp2x* amplicon from IU14927)	Str^R	This Study
IU15096	D39 Δ*cps rpsL1 ftsW*-L_0-*ht*-P_c-*erm* (IU1824 X *ftsW*-L_0-*ht*-P_c-*erm* from PCR fusion)	Erm^R Str^R	This Study
IU15141	D39 Δ*cps rpsL1 ftsZ*-L_2-*sfgfp* Δ*murA1* (IU15179 X *ftsZ*-L_2-*sfgfp* from IU9985)	Str^R	This study
IU15153	D39 Δ*cps rpsL1 ftsZ*$^+$-P_c-[*kan-rpsL*$^+$] *ftsZ*$^+$//*bgaA*'::*tet*-P_{Zn}-*ftsZ*(D214A) (IU14850 X *ftsZ*$^+$-P_c-[*kan-rpsL*$^+$] amplicon from IU7614)	Kan^R Tet^R	This study
IU15171	D39 Δ*cps rpsL1 ftsW*-L_0-*ht*-P_c-*erm isfgfp*-L_1-*pbp2x* (IU11157 X *ftsW*-L_0-*ht*-P_c-*erm* amplicon from IU15096)	Erm^R Str^R	This Study
IU15173	D39 Δ*cps rpsL1 ftsW*-L_0-*ht*-P_c-*erm* Δ*murA1* (IU13536 X *ftsW*-L_0-*ht*-P_c-*erm* amplicon from IU15096)	Erm^R Str^R	This Study
IU15175	D39 Δ*cps rpsL1 ftsW*-L_0-*ht*-P_c-*erm* *ftsZ*$^+$//*bgaA*'::*tet*-P_{Zn}-*ftsZ*(D214A) (IU14850 X *ftsW*-L_0-*ht*-P_c-*erm* amplicon from IU15096)	Erm^R Str^R Tet^R	This Study
IU15179	D39 Δ*cps rpsL1 ftsZ*$^+$-P_c-[*kan-rpsL*$^+$] Δ*murA1* (IU13536 X *ftsZ*$^+$-P_c-[*kan-rpsL*$^+$] from IU7614)	Kan^R	This study

IU15181	D39 Δ*cps rpsL1 ftsZ*-L$_2$-*sfgfp ftsZ*$^+$//*bgaA*'::*tet*-P$_{Zn}$-*ftsZ*(D214A) (IU15153 X *ftsZ*-L$_2$-*sfgfp* amplicon from IU9985)	Str^R Tet^R	This study
IU15231[m]	D39 Δ*cps rpsL1* Δ*pbp2x*::P$_c$-[*kan-rpsL*$^+$]//*bgaA*'::*tet*-P$_{Zn}$-*pbp2x*$^+$ (IU14330 X Δ*pbp2x*::P$_c$-[*kan-rpsL*$^+$] amplicon from PCR fusion)	Kan^R Tet^R	This study
IU15246[m]	D39 Δ*cps rpsL1 pbp2x*(S337A)//*bgaA*'::*tet*-P$_{Zn}$-*pbp2x*$^+$ (IU15231 X *pbp2x*(S337A) amplicon from PCR fusion)	Str^R Tet^R	This study
IU15599[l]	D39 Δ*cps rpsL1 iht*-L$_6$-*ftsZ* (IU7616 X *iht*-L$_6$-*ftsZ* amplicon from PCR fusion)	Str^R	This Study
IU15666	D39 Δ*cps rpsL1* Δ*mapZ*::P$_c$-[*kan-rpsL*$^+$] *ftsZ*-L$_2$-*sfgfp* (IU9985 X Δ*mapZ*::P$_c$-[*kan-rpsL*$^+$] amplicon from IU9086)	Kan^R	This Study
IU15674	D39 Δ*cps rpsL1* Δ*mapZ ftsZ*-L$_2$-*sfgfp* (IU15666 X Δ*mapZ* amplicon from IU9175)	Str^R	This Study
K767	D39 Δ*cps* Δ*murA1(spd_0967)*::P$_c$-[*kan-rpsL*$^+$] (IU1945 X Δ*murA1(spd_0967)*::P$_c$-[*kan-rpsL*$^+$] from PCR fusion)	Kan^R	This study

**Primers used for strain construction**			
Primer	Sequence (5' to 3')	Template[h i]	Amplicon Product
**For construction of IU5557 (*bgaA'*::*kan*-t1t2-P$_{fcsK}$-*ftsZ*$^+$)**			
TT657	CGCCCCAAGTTCATCACCAATGACATCAAC	IU4888	*bgaA'*-P$_c$-*kan*-t1t2-P$_{fcsk}$
TT201	CAGCTGTATCAAATGAAAATGTCATTTTTCTTCTCTCTTCGTCCTTGATTAACTT		
TT202	ATCAAGGACGAAGAGAGAAGAAAAATGACATTTTCATTTGATACAGCTGCTG	D39	*ftsZ*$^+$
TT203	ACTGGTTTATGAGAAAGTAAGTTCTTTTATTAACGATTTTTGAAAAATGGAGGTGTATC		
TT396	CCTCCATTTTTCAAAAATCGTTAGAAGAACTTACTTTCTCATAAACCAGTTGCTG	D39	*bgaA'*
CS121	GCTTTCTTGAGGCAATTCACTTGGTGC		
**For construction of IU5648 (*divIVA*$^+$-P$_c$-[*kan-rpsL*$^+$])**			
SC219	TAACCGTCCAGTTATTATTAAGTAAGTGAGGAATAGAATGCCAATTACATCATTAG	D39	5' flanking containing *divIVA*$^+$
TT239	CATTATCCATTAAAAATCAAACGGATCCTACTACTTCTGGTTCTTCATACATTGGGC		
Kan rpsL forward	TAGGATCCGTTTGATTTTTAATGGATAATG	P$_c$-[*kan-rpsL*$^+$] cassette	P$_c$-[*kan-rpsL*$^+$]
Kan rpsL	GGGCCCCTTTCCTTATGCTTTTG		

reverse			
TT240	CAAAAGCATAAGGAAAGGGGCCCCTCC AGTGCATCCGACAGGTCC	D39	3’ flanking *downstream divIVA*$^{+}$
TT238	TTCAGCAAGGGCTGACTCAGATGACCAT GA		
**For construction of IU6506 (*pbp2x*-L_0-FLAG^{3}-P_c-*erm*)**			
TT345	GTGACCCAGACGCAAATGATTCGTGCCT TT	D39	5’ flanking fragment
TT347	CGGAGCCAGCGGAACCGTCTCCTAAAG TTAATGTAATTTTTTTAATGTCCTTG		
TT348	CATTAAAAAAATTACATTAACTTTAGGAG ACGGTTCCGCTGGCTCCGC	IU5544	L_0-FLAG^{3}-P_c-*erm*
TT349	CTGATGGAAATAAACATATTATTATTTCC TCCCGTTAAATAATAGATAACTATTAAAA AT		
TT350	ATAGTTATCTATTATTTAACGGGAGGAAA TAATAATATGTTTATTTCCATCAGTGCTG GA	D39	3’ flanking fragment
TT346	AGAAGTCAACCTTCCACTCGCTCCAAGG AT		
**For construction of IU6541 (*pbp2x*-FLAG-P_c-*erm*)**			
TT345	GTGACCCAGACGCAAATGATTCGTGCCT TT	D39	5’ flanking containing *pbp2x*-FLAG
SV003	TTTATCATCATCATCTTTATAATCGTCTC CTAAAGTTAATGTAATTTTTTTAATGTCCT T		
SV004	TAAAAAAATTACATTAACTTTAGGAGACG ATTATAAAGATGATGATGATAAATAACCG GG	IU6506	3’ flanking *downstream* FLAG-P_c-*erm*
TT346	AGAAGTCAACCTTCCACTCGCTCCAAGG AT		
**For construction of IU7054 (*bgaA’*::*kan*-t1t2-P_{ftsA}-*ftsZ*$^{+}$)**			
P146	TGGCCATTCATCGCTGGTCGTGCTGAAA T	IU6397	*bgaA*’::*kan*-t1t2-P_{ftsA}
TT393	CAGCTGTATCAAATGAAAATGTCATTACA TCGCTTCCTCTCTATCTTCCAAGT		
TT394	GGAAGATAGAGAGGAAGCGATGTAATG ACATTTTCATTTGATACAGCTGCTG	IU5557	3’ flanking containing *ftsZ*$^{+}$-*bgaA*’
CS121	GCTTTCTTGAGGCAATTCACTTGGTGC		
**For construction of IU7616 (*ftsA*$^{+}$-P_c-[*kan-rpsL*$^{+}$])**			
TT457	ATTGTGGATGGTTTCCAAGGGATTCGTG AC	D39	5’ flanking containing *ftsA*$^{+}$
TT581	CATTATCCATTAAAAATCAAACGGATCCT ATTATTCGTCAAACATGCTTCCGATC		
Kan rpsL	TAGGATCCGTTTGATTTTTAATGGATAAT G	P_c-[*kan-rpsL*$^{+}$]	P_c-[*kan-rpsL*$^{+}$]

forward		cassette	
Kan rpsL reverse	GGGCCCCTTTCCTTATGCTTTTG		
TT582	CAAAAGCATAAGGAAAGGGGCCCAATAAACCGAAATTAGCAGATCGTTTCC	D39	3’ flanking repeating 3’ 60 bp *ftsA*$^+$
TT462	CAGATGTTCACTCCTTGACCTGCTGCCTGG		
**For construction of IU7814 (Δ*ftsZ*::*aad9*)**			
AL366	GGCATGATGGGGGTTCGCCTTGAAATGCG	D39	5’ upstream of *ftsZ* (no *ftsZ* nt)
TT204	CGTATGTATTCAAATATATCCTCCTCACAATTTATTTTTCCTCTTTATTCGTCAAACATG		
TT205	TTGACGAATAAAGAGGAAAAATAAATTGTGAGGAGGATATATTTGAATACATACGAACA	IU4888	Middle-*aad9* +extra 9 bp of 3’ *mreD*
TT206	CTCGACTGGAGAAACGACTGAATGTCGTTCTTATAATTTTTTTAATCTGTTATTTAAA		
TT207	ACAGATTAAAAAAATTATAAGAACGACATTCAGTCGTTTCTCCAGTCGAGCG	D39	87 bp 3’ *ftsZ*+stop + downstream region
TT166	TCATTGGGAGAGCCGGTTCCTGTGAAGAAT		
**For construction of IU8122 (*bgaA*’::*tet*-P_{Zn}-*ftsZ*$^+$)**			
TT657	CGCCCCAAGTTCATCACCAATGACATCAAC	IU3966	*bgaA*’::*tet*-P_{Zn}-
AJP32	ACATCGCTTCCTCTCTATCTTCCTTGTTATAATAGATTTATGAACACCTTGTTCATTATC		
AJP33	AACAAGGTGTTCATAAATCTATTATAACAAGGAAGATAGAGAGGAAGCGATGTAATGA	IU7054	*ftsZ*$^+$-*bgaA’*
CS121	GCTTTCTTGAGGCAATTCACTTGGTGC		
**For construction of IU8845 (*ftsZ*-L_2-*gfp*)**			
TT165	AGTGGTGCCGATATGGTCTTCATCACTGCT	D39	3’ *ftsZ*
TT695	CATCTGCAGGAACTCGATGTCTAGTTTACGATTTTTGAAAAATGGAGGTGTATCC		
TT693	AAACTAGACATCGAGTTCCTGCAGATGATTTCTAAAGGTGAAGAATTGTTTACAGG	pUC57-*gfp* (*Sp*)	L_2-*gfp*
TT694	TTACTTAACGATTTTTGAAAAATGTTATTTATACAATTCATCCATACCATGTGTAATACC		
TT696	CATGGTATGGATGAATTGTATAAATAACATTTTTCAAAAATCGTTAAGTAAATGAATGTA	D39	3’ downstream of *ftsZ*

TT166	TCATTGGGAGAGCCGGTTCCTGTGAAGAAT		
**For construction of IU8900 (*ftsW*$^+$-P$_c$-[*kan-rpsL*$^+$])**			
TT482	GTTGGATTTCGGTTGCAGGAGTAACTATTCAGCCA	D39	5' flanking containing *ftsW*$^+$
TT700	CATTATCCATTAAAAATCAAACGGATCCTACTACTTCAACAGAAGGTTCATTGGTTGAT		
Kan rpsL forward	TAGGATCCGTTTGATTTTTAATGGATAATG	P$_c$-[*kan-rpsL*$^+$] cass-ette	P$_c$-[*kan-rpsL*$^+$]
Kan rpsL reverse	GGGCCCCTTTCCTTATGCTTTTG		
TT701	CAAAAGCATAAGGAAAGGGGCCCGATAAAGAAAGGATAGTTTATGTCTCTTCAAAAATT	D39	3' flanking downstream of *ftsW*$^+$
TT483	TTCCATCACGGTCACCACCTATCCACATAC		
**For construction of IU8918 (*ftsW*-L$_2$-*gfp*)**			
TT482	GTTGGATTTCGGTTGCAGGAGTAACTATTCAGCCA	D39	3' *ftsW*-L$_2$
TT710	AATCATCTGCAGGAACTCGATGTCTAGTTTCTTCAACAGAAGGTTCATTGGTTGATTTT		
TT693	AAACTAGACATCGAGTTCCTGCAGATGATTTCTAAAGGTGAAGAATTGTTTACAGG	IU8845	L$_2$-*gfp*
TT711	GACATAAACTATCCTTTCTTTATCCTATTTATACAATTCATCCATACCATGTGTAATACC		
TT712	ACATGGTATGGATGAATTGTATAAATAGGATAAAGAAAGGATAGTTTATGTCTCTTCAAA	D39	3' downstream of *ftsW*
TT483	TTCCATCACGGTCACCACCTATCCACATAC		
**For construction of IU8921 (P$_c$-[*kan-rpsL*$^+$]-*pbp2x*$^+$)**			
TT713	CGCCACTGGACATGCGGATGAATCAGGATG	D39	5' fragment upstream of *pbp2x*
TT714	ATCCATTAAAAATCAAACGGATCCTAATCTTACTCCGCTATTCTAATATTTTCATTGTTT		
TT715	TGAAAATATTAGAATAGCGGAGTAAGATTAGGATCCGTTTGATTTTTAATGGATAATG	P$_c$-[*kan-rpsL*$^+$] cassette	P$_c$-[*kan-rpsL*$^+$]
Kan rpsL reverse	GGGCCCCTTTCCTTATGCTTTTG		

TT716	CAAAAGCATAAGGAAAGGGGCCCGAAAATATTAGAATAGCGGAGTAAGATATGAAGTG	D39	3’ fragment including *pbp2x*$^+$
TT717	GTCGGTCGTTGCGTTGTTGCCAGAATTTCC		
**For construction of IU9020 (*gfp*-L$_1$-*pbp2x*)**			
TT713	CGCCACTGGACATGCGGATGAATCAGGATG	D39	5‘ fragment upstream of *pbp2x*
TT719	AACAATTCTTCACCTTTAGAAATCATATCTTACTCCGCTATTCTAATATTTTCATTGTTT		
TT720	TGAAAATATTAGAATAGCGGAGTAAGATATGATTTCTAAAGGTGAAGAATTGTTTACAGG	IU8845	*gfp*-L$_1$
TT721	TGTCCACTTCATTCCGGATCCCTCGAGTTTATACAATTCATCCATACCATGTGTAATACC		
TT722	GATGAATTGTATAAACTCGAGGGATCCGGAATGAAGTGGACAAAAAGAGTAATCCGTTAT	D39	3’ fragment including *pbp2x*
TT717	GTCGGTCGTTGCGTTGTTGCCAGAATTTCC		
**For construction of IU9023 (P$_c$-[*kan-rpsL*$^+$]-*pbp2b*$^+$)**			
TT452	GGAGGGTTGGCTGTGGGTGGCTACAAGAAC	D39	5‘ fragment upstream of *pbp2b*
TT723	CATTATCCATTAAAAATCAAACGGATCCTACCACAATTTGAGTAGAAAATTATGGAAAAG		
Kan rpsL forward	TAGGATCCGTTTGATTTTTAATGGATAATG	P$_c$-[*kan-rpsL*$^+$] cassette	P$_c$-[*kan-rpsL*$^+$]
Kan rpsL reverse	GGGCCCCTTTCCTTATGCTTTTG		
TT724	CAAAAGCATAAGGAAAGGGGCCCTACAATTAAGAGTAAGATTTTAAGTTAGAAATGAGAC	D39	3’ fragment including *pbp2b*$^+$
TT452	GGAGGGTTGGCTGTGGGTGGCTACAAGAAC		
**For construction of IU9077 (*ezrA*$^+$-P$_c$-[*kan-rpsL*$^+$])**			
TT192	ATCGTGTTCCAGCCTTGGTTACGACGCTTT	D39	3’ *ezrA*
TT741	CATTATCCATTAAAAATCAAACGGATCCTATAGAAAAAGATTTGATTCTGCTCCTCACAC		

Kan rpsL forward	TAGGATCCGTTTGATTTTTAATGGATAATG	P_c-[*kan-rpsL*$^+$] cassette	P_c-[*kan-rpsL*$^+$]
Kan rpsL reverse	GGGCCCCTTTCCTTATGCTTTTG		
TT742	ACGTCCAAAAGCATAAGGAAAGGGGCCCTAGTTGTGGGGAGATTTACTTCATTTTCTCCT	D39	3' flanking fragment downstream of *ezrA*$^+$
TT330	GAGGAGTTCGGACTCGACTCTCTCCTTCAAGAA		
**For construction of IU9086 (Δ*mapZ*::P_c-[*kan-rpsL*$^+$])**			
P1523	GAGGTCTCTATTCTCAAAGATGTGGCAACTGTC	D39	Upstream of *mapZ* and 5' 57 bp of *mapZ*
P1524	CATTATCCATTAAAAATCAAACGGATCCTAATCAAATTGCGGTTCTTGAGCTTCT		
Kan rpsL forward	TAGGATCCGTTTGATTTTTAATGGATAATG	P_c-[*kan-rpsL*$^+$] cassette	P_c-[*kan-rpsL*$^+$]
Kan rpsL reverse	GGGCCCCTTTCCTTATGCTTTTG		
P1525	TCCAAAAGCATAAGGAAAGGGGCCCTGTAAGACAGGCTACTTTGTCGGAAATGGC	D39	3' 60 bp and stop codon of *mapZ* and 3' downstream *mapZ*
P1526	AATTGCATATCACCGTACTCAATACCATTGTG		
**For construction of IU9090 (*mapZ*-L_0-FLAG^3-P_c-*erm*)**			
AJP81	GCTGGAAGGTAGTCGTGAACATACGCTTGC	D39	*3' mapZ*
AJP82	CGGAGCCAGCGGAACCGTAGTCCAAGTCATCCGCATGACCAGCGC		
AJP83	TGGTCATGCGGATGACTTGGACTACGGTTCCGCTGGCTCCGC	IU5456	L_0-FLAG^3-P_c-*erm*
AJP84	AAGAAAGAATTTTGTAACGACTGCTTATTTCCTCCCGTTAAATAATAGATAACTATTAAA		
AJP85	TTATCTATTATTTAACGGGAGGAAATAAGCAGTCGTTACAAAATTCTTTCTTTTCAA	D39	3' downstream of *mapZ*
P1526	AATTGCATATCACCGTACTCAATACCATTGTG		
**For construction of IU9094 (P_c-[*kan-rpsL*$^+$]-*mapZ*$^+$)**			
P1523	GAGGTCTCTATTCTCAAAGATGTGGCAACTGTC	D39	upstream of *mapZ*
AJP90	CCATTAAAAATCAAACGGATCCTACTATTTTACCTCTTGCCGTTTGACAC		

Kan rpsL forward	TAGGATCCGTTTGATTTTTAATGGATAATG	P_c-[*kan-rpsL*$^+$] cassette	P_c-[*kan-rpsL*$^+$]
Kan rpsL reverse	GGGCCCCTTTCCTTATGCTTTTG		
AJP91	TCCAAAAGCATAAGGAAAGGGGCCCCAGCGTGTCAAACGGCAAGAG	D39	*mapZ*$^+$-*gnd'*
P1526	AATTGCATATCACCGTACTCAATACCATTGTG		
**For construction of IU9164 (*gfp*-L_1-*stkP*)**			
TT546	AGAGAGTCATCCCGAGTTCGAGCAGGTAAA	D39	5' fragment including upstream of *stkP*
TT761	AACACCTGTAAACAATTCTTCACCTTTAGAAATCATTCTGCATCCTCCTCGTTCATAGA		
TT756	ATGATTTCTAAAGGTGAAGAATTGTTTACAGG	IU9020	*gfp*-L_1
TT757	TCCGGATCCCTCGAGTTTATACAATTCATCC		
TT762	GGATGAATTGTATAAACTCGAGGGATCCGGAATGATCCAAATCGGCAAGATTTTTG	D39	Downstream including *stkP*
TT547	CGGTGCTTGTGGTTGGTAAGTTTCCTCTGT		
**For construction of IU9167 (*divIVA*-L_2-*gfp*)**			
SC219	TAACCGTCCAGTTATTATTAAGTAAGTGAGGAATAGAATGCCAATTACATCATTAG	D39	5' fragment including *divIVA*$^+$
TT734	TGCAGGAACTCGATGTCTAGTTTCTTCTGGTTCTTCATACATTGGGCC		
TT693	AAACTAGACATCGAGTTCCTGCAGATGATTTCTAAAGGTGAAGAATTGTTTACAGG	IU8845	L_2-*gfp* cassette
TT735	TTATTTATACAATTCATCCATACCATGTGTAATACC		
TT736	GGTATTACACATGGTATGGATGAATTGTATAAATAATCATCCAGTTGGCCCAATGTATG	D39	3' fragment downstream of *divIVA*$^+$
TT238	TTCAGCAAGGGCTGACTCAGATGACCATGA		
**For construction of IU9175 (Δ*mapZ*)**			
P1523	GAGGTCTCTATTCTCAAAGATGTGGCAACTGTC	D39	Upstream of *mapZ* + 5' 57 bp of *mapZ*
AJP92	CCGACAAAGTAGCCTGTCTTACAATCAAATTGCGGTTCTTGAGCTTCT		
AJP93	GCTCAAGAACCGCAATTTGATTGTAAGACAGGCTACTTTGTCGGAAATGGC	D39	3' 60 bp and stop codon of *mapZ* + downstream of

P1526	AATTGCATATCACCGTACTCAATACCATT GTG		*mapZ*
**For construction of IU9182 (*gfp*-L_1-*mapZ*)**			
P1523	GAGGTCTCTATTCTCAAAGATGTGGCAA CTGTC	D39	5' fragment including upstream of *mapZ*
AJP94	AAACAATTCTTCACCTTTAGAAATCATGA GTATCCCTTTCTATTTTACCTCTTGCC		
AJP95	CAAGAGGTAAAATAGAAAGGGATACTCA TGATTTCTAAAGGTGAAGAATTGTTTACA GG	IU9020	*gfp*-L_1
AJP96	TTATGACGATTTCGTCTTTTTTTACTTCC GGATCCCTCGAGTTTATACAATTCATCCA TA		
AJP97	ATGAATTGTATAAACTCGAGGGATCCGG AAGTAAAAAAAGACGAAATCGTCATAAA AAAG	D39	3' fragment including *mapZ*$^{+}$-*gnd'*
P1526	AATTGCATATCACCGTACTCAATACCATT GTG		
**For construction of IU9965 (*sfgfp*-L_1-*pbp2b*)**			
TT452	GGAGGGTTGGCTGTGGGTGGCTACAAG AAC	IU9622	5' fragment upstream from *pbp2b* and first 15 bp of *pbp2b*
TT786	GTGAACAGCTCTTCTCCTTTCATACAAAT CAGTCTCATTTCTAACTTAAAATCTTACT CT		
TT787	ATTTTAAGTTAGAAATGAGACTGATTTGT ATGAAAGGAGAAGAGCTGTTCACAGG	IU9683	*sfgfp*-L_1 middle fragment
TT784	CATTCCGGATCCCTCGAGCTTATAAAGC TCATCCATGCCGTGA		
TT788	CGGCATGGATGAGCTTTATAAGCTCGAG GGATCCGGAATGAGA	IU9622	3' fragment containing L_1-*pbp2b* starting at 16 bp of *pbp2b*
TT725	CCCTTACTACCTTCCTCAATTGTATCCAC GC		
**For construction of IU9985 (*ftsZ*-L_2-*sfgfp*)**			
TT165	AGTGGTGCCGATATGGTCTTCATCACTG CT	IU8845	5' fragment containing *ftsZ*-L_2
TT695	CATCTGCAGGAACTCGATGTCTAGTTTA CGATTTTTGAAAAATGGAGGTGTATCC		
TT789	AACTAGACATCGAGTTCCTGCAGATGAA AGGAGAAGAGCTGTTCACAGG	IU9683	middle fragment L_2-*sfgfp*
TT790	TTTACTTAACGATTTTTGAAAAATGTTACT TATAAAGCTCATCCATGCCGTGA		
TT791	CGGCATGGATGAGCTTTATAAGTAACAT TTTTCAAAAATCGTTAAGTAAATGAATG	IU8845	3' fragment downstream of *ftsZ*$^{+}$
TT166	TCATTGGGAGAGCCGGTTCCTGTGAAGA AT		

**For construction of IU10035 (*gfp*-L_1-*ftsA*)**			
TT750	GGTCATAGGGGGCAATATCTTGACTAAGAAG	D39	5’ fragment containing upstream of *ftsA*
TT754	CCTGTAAACAATTCTTCACCTTTAGAAATCATTACATCGCTTCCTCTCTATCTTCCAAGT		
TT756	ATGATTTCTAAAGGTGAAGAATTGTTTACAGG	IU9020	*gfp*-L_1
TT757	TCCGGATCCCTCGAGTTTATACAATTCATCC		
TT755	GAATTGTATAAACTCGAGGGATCCGGAATGGCTAGAGAAGGCTTTTTACAGGTCTAG	D39	3’ fragment including L_1 and *ftsA*$^+$
TT753	GCCTTCCGCTAATTTGCGAGAGGTTTTCAA		
**For construction of IU10063 (*bgaA’*::*tet*-P_{Zn}-*pbp2x*$^+$)**			
P146	TGGCCATTCATCGCTGGTCGTGCTGAAAT	IU8122	5’ fragment *bgaA’*::*tet*-P_{Zn}
BR66	AACGGATTACTCTTTTTGTCCACTTCATTACATCGCTTCCTCTCTATCTTCCTTGTTATAATA		
BR65	ACAAGGAAGATAGAGAGGAAGCGATGTAATGAAGTGGACAAAAAGAGTAATCCGTTATG	D39	middle fragment *pbp2x*$^+$
BR68	GGTTTATGAGAAAGTAAGTTCTTTTAGTCTCCTAAAGTTAATGTAATTTTTTTAATGTCC		
BR67	AAAAAATTACATTAACTTTAGGAGACTAAAAGAACTTACTTTCTCATAAACCAGTTGCTG	IU8122	3’ *bgaA* fragment
CS121	GCTTTCTTGAGGCAATTCACTTGGTGC		
**For construction of IU10612 (*ftsZ*(G107S))**			
TT457	ATTGTGGATGGTTTCCAAGGGATTCGTGAC	D39	5’ fragment containing GGA to AGT mutation
AJP12	CTCCAGTTCCAGAGCCTCCACTCATACCAGCAGTGATGAA		
AJP13	TTCATCACTGCTGGTATGAGTGGAGGCTCTGGAACTGGAG	D39	3’ fragment containing GGA to AGT mutation
TT166	TCATTGGGAGAGCCGGTTCCTGTGAAGAAT		
**For construction of IU11005 (*sfgfp*-L_1-*mltG*)**			
P1348	TCTTCTTGCAGCCTTGAAAGAGGTGGCAGT	D39	5’ fragment containing region upstream of *mltG*
TT899	CCTGTGAACAGCTCTTCTCCTTTCATAAGTTTTTCCTCCTTGTTGATAATCCATAA		

TT900	ATGAAAGGAGAAGAGCTGTTCACAGGTG TTGTGCCGAT	IU9965	*sfgfp*-L_1
TT901	TCCGGATCCCTCGAGCTTATAAAGCTCA TCCATGCC		
TT902	GGATGAGCTTTATAAGCTCGAGGGATCC GGAAGTGAAAAGTCAAG	IU10228	3’ fragment containing L_1-*mltG* (no ATG start)
TT412	CGACCAAGGAAGCAATGGTCAACAACTC AT		
**For construction of IU10254 (*ezrA*-L_0-*sfgfp*)**			
TT192	ATCGTGTTCCAGCCTTGGTTACGACGCT TT	D39	5’ fragment containing 3’ *ezrA*-L_0
AJP120	CCTGTGAACAGCTCTTCTCCTTTGCCAG AACCAGCAGCGGAG		
AJP121	CCGCTGCTGGTTCTGGCAAAGGAGAAG AGCTGTTCACAGGTGT	IU9985	L_0-*sfgfp*
AJP122	TCCTCACACAATAAAATCTTTTTCTTTTAT TACTTATAAAGCTCATCCATGCCGTG		
AJP123	CGGCATGGATGAGCTTTATAAGTAATAA AAGAAAAAGATTTTATTGTGTGAGGAGC	D39	3’ fragment containing downstream of *ezrA*
TT330	GAGGAGTTCGGACTCGACTCTCTCCTTC AAGAA		
**For construction of IU10449 (*ezrA*-L_0-*gfp*)**			
TT192	ATCGTGTTCCAGCCTTGGTTACGACGCT TT	IU5456	5’ fragment containing 3’ *ezrA*-L_0
AJP136	AAACAATTCTTCACCTTTAGAAATCATGC CAGAACCAGCAGCGGAG		
AJP137	CCGCTGCTGGTTCTGGCATGATTTCTAA AGGTGAAGAATTGTTTACAGG	IU8845	L_0-*gfp*
TT759	ACACAATAAAATCTTTTTCTTTTATTATTT ATACAATTCATCCATACCATGTGTAATAC C		
TT760	ACATGGTATGGATGAATTGTATAAATAAT AAAAGAAAAAGATTTTATTGTGTGAGGA GCA	D39	3’ fragment containing downstream of *ezrA*
TT330	GAGGAGTTCGGACTCGACTCTCTCCTTC AAGAA		
**For construction of IU11119 (*ezrA*-L_0-*sfgfp*-P_c-*cat*)**			
TT192	ATCGTGTTCCAGCCTTGGTTACGACGCT TT	IU10254	3’ *ezrA*-L_0-*sfgfp*
AJP198	ATCCATTAAAAATCAAACGGATCCTATTA CTTATAAAGCTCATCCATGCCGTGAGT		
Kan rpsL forward	TAGGATCCGTTTGATTTTTAATGGATAAT G	IU10294	P_c-*cat*

Kan rpsL reverse	GGGCCCCTTTCCTTATGCTTTTG		
AJP199	CAAAAGCATAAGGAAAGGGGCCCTAAAAGAAAAAGATTTTATTGTGTGAGGAGCAG	D39	downstream of *ezrA*
TT330	GAGGAGTTCGGACTCGACTCTCTCCTTCAAGAA		
**For construction of IU11157 (*isfgfp*-L_1-*pbp2x*)**			
TT801	GCTAGTCTGACAGCCTATGAAGTGGTTAATCATTATGAC	D39	5' fragment upstream *pbp2x* of containing i-tag
AJP188	AAAAAATTTCCAAACCTTTTTTATCCATATCTTACTCCGCTATTCTAATATTTTCATTGT		
AJP168	GATAAAAAAGGTTTGGAAATTTTTTTGGCTTCTAAAGGAGAAGAGCTGTTCACAGGTGTT	IU9965	Middle fragment containing *isfgfp*-L_1
AJP187	TCCGGATCCCTCGAGCTTATAAAGCTCATCCATGCCGTGA		
AJP189	AGCTCGAGGGATCCGGAATGAAGTGGACAAAAAGAGTAATCCGTTAT	D39	3' fragment containing L_{1-}*pbp2x*
TT717	GTCGGTCGTTGCGTTGTTGCCAGAATTTCC		
**For construction of IU11594 (*gpsB*-L_0-*sfgfp*-P_c-*cat*)**			
TT196	GCCAAGCCCTGAGACAAATAGTAGTCGTTGGT	IU11516	*gpsB*-L_0
JC23	ACCTGTGAACAGCTCTTCTCCTTTGCCAGAACCAGCAGCGGAG		
JC24	CTCCGCTGCTGGTTCTGGCAAAGGAGAAGAGCTGTTCACAGGT	IU11119	L_0-*sfgfp*-P_c-*cat*
JC25	ATCCAAAAATTGCACATCTCAAATAACTACGGGCCCCTTTCCTTATGCTTT		
JC26	AAAGCATAAGGAAAGGGGCCCGTAGTTATTTGAGATGTGCAATTTTTGGAT	D39	Downstream of *gpsB*$^+$
TT197	TTTGATACGATCTGCTGCCCGAAGCCAAAGGT		
**For construction of IU13315 (*bgaA'*::*tet*-P_{Zn}-*ftsZ*-L_2-*sfgfp*)**			
TT657	CGCCCCAAGTTCATCACCAATGACATCAAC	IU8122	5' *bgaA'*::*tet*-P_{Zn}-
TT988	CAGCTGTATCAAATGAAAATGTCATTACATCGCTTCCTCTCTATCTTCCTTGT		
TT989	GGAAGATAGAGAGGAAGCGATGTAATGACATTTTCATTTGATACAGCTGCTG	IU9985	*ftsZ*-L_2-*sfgfp* middle fragment
TT921	CTGGTTTATGAGAAAGTAAGTTCTTTTACTTATAAAGCTCATCCATGCCGTGAG		
TT922	CGGCATGGATGAGCTTTATAAGTAAAAGAACTTACTTTCTCATAAACCAGTTGCT	D39	3' downstream *bgaA'*

CS121	GCTTTCTTGAGGCAATTCACTTGGTGC		
**For construction of IU13536 (Δ*murA1*)**			
P1554	GATTTTGTGGTACGACGGGCATGTATAGCG	D39	5’ fragment containing 60 nt 5’ of *murA1*
JQ344	TAAATTACGTAATTTTTCGATAATATCAGAACCACTAATAGTGATTTCACCTTGCAGTGG		
JQ345	CCACTGCAAGGTGAAATCACTATTAGTGGTTCTGATATTATCGAAAAATTACGTAATTTA	D39	3’ fragment containing 60 nt 3’ of *murA1*
P1555	TGAACCTGAAATCCCCCTGTAACCAGAACT		
**For construction of IU13662 (*ftsA’*-L_8-*sfgfp*-L_8-*ftsA’*)**			
TT628	GGTGACAGGTGGCGATTACGGATTTTTGAC	D39	5’ fragment including 801 bp of *ftsA* interrupted by L_8-
YT121	TCCTTGGCCTGAGCCCGGTCCCTGGCCAGATCCCTCGAGTGCAAGAGGCGGATAGGCTTC		
YT122	TGGCCAGGGACCGGGCTCAGGCCAAGGAAGCGGCATGAAAGGAGAAGAGCTGTTCACAGG	IU9985	L_8-*sfgfp*-L_8
YT123	TGACCAGATCCAGGACCTTGTCCGCTACCCTCAAGCTTATAAAGCTCATCCATGCCGTGA		
YT124	GGACAAGGTCCTGGATCTGGTCAAGGCAGTGGGAGCAAAGAAACCTTCCAAGTAGAGGTT	D39	L_8-*ftsA*’ (last 570 bp + TAA stop) and downstream of *ftsA*$^+$
TT612	CCTCCTGCACCCAAACCACGAGTCAATTTA		
**For construction of IU13910 (*ht*-L_6-*pbp2x*)**			
TT801	GCTAGTCTGACAGCCTATGAAGTGGTTAATCATTATGAC	D39	5’ upstream of *pbp2x*
YT105	GGAAAACCAGTACCAATTTCAGCCATATCTTACTCCGCTATTCTAATATTTTCATTGTTT		
YT101	ATGGCTGAAATTGGTACTGGTTTTCCATTT	gBlock synthetic DNA[j]	Middle *ht*-L_6
YT104	ACCAGAACCTTGACCAGATCCTGGTCCTTG		
YT106	CAAGGACCAGGATCTGGTCAAGGTTCTGGTAAGTGGACAAAAAGAGTAATCCGTTATGCG	D39	3’ including *pbp2x*
TT717	GTCGGTCGTTGCGTTGTTGCCAGAATTTCC		

**For construction of IU14117 (*ezrA*-L$_0$-*mneongreen*-P$_c$-*cat*)**			
TT192	ATCGTGTTCCAGCCTTGGTTACGACGCTTT	IU10254	5’ containing *ezrA*-L$_0$
AJP370	TATTATCCTCCTCTCCTTTCGAAACGCCAGAACCAGCAGCGGAGC		
AJP371	CCGCTGCTGGTTCTGGCGTTTCGAAAGGAGAGGAGGATAATATG	YB7656[k]	L$_0$-*mneongreen*
AJP372	TCCATTAAAAATCAAACGGATCCTACTTATAGAGTTCATCCATACCCATCACGT		
Kan rpsL forward	TAGGATCCGTTTGATTTTTAATGGATAATG	IU11119	P$_c$-*cat* and downstream of *ezrA*
TT330	GAGGAGTTCGGACTCGACTCTCTCCTTCAAGAA		
**For construction of IU14288 (*ftsZ*-L$_5$-*ht*-*erm*)**			
TT165	AGTGGTGCCGATATGGTCTTCATCACTGCT	IU13408	5’ fragment containing *ftsZ*-L$_5$
AJP385	GGAAAACCAGTACCAATTTCAGCTCCAGCTTTAGCTGCAGCTTCTCCAC		
AJP386	GAGAAGCTGCAGCTAAAGCTGGAGCTGAAATTGGTACTGGTTTTCCATTT	IU13910	middle containing L$_5$-*ht*
YT103	ACCAGAAATTTCCAATGTAGACAACCAACG		
AJP387	GGTTGTCTACATTGGAAATTTCTGGTTAACCCGGAGGAATTTTCATATGAACAA	IU13408	3’ fragment containing *erm* and downstream of *ftsZ*
TT166	TCATTGGGAGAGCCGGTTCCTGTGAAGAAT		
**For construction of IU14290 (*pbp1a*-L$_0$-*ht*-P$_c$-*erm*)**			
TT225	AGCCGTGGAAACTCTAAACAAGGTCGGACT	IU5544	5’ fragment containing *pbp1a*-L$_0$
AJP388	GGAAAACCAGTACCAATTTCAGCGCCAGAACCAGCAGCGGAGC		
AJP389	GCTCCGCTGCTGGTTCTGGCGCTGAAATTGGTACTGGTTTTCCATTT	IU13910	middle containing L$_0$-*ht*
YT103	ACCAGAAATTTCCAATGTAGACAACCAACG		
AJP390	GGTTGTCTACATTGGAAATTTCTGGTTAACCGGGCCCAAAATTTGTTT	IU5544	3’ fragment containing P$_c$-*erm* and downstream of *pbp1a*
P235	AGGCAAGCCTGCAACCATGGTCTTGAAA		
**For construction of IU14404 (*ezrA*-L$_0$-*ht*-P$_c$-*erm*)**			
TT192	ATCGTGTTCCAGCCTTGGTTACGACGCTTT	D39	5’ containing *ezrA*-L$_0$
TT193	CGGAGCCAGCGGAACCAAAACGAATCGTTTCACGTGTTTTC		

JQ179	GGTTCCGCTGGCTCCGCTGCTGGTTCTGGC	IU14290	middle containing L_0-*ht*-P_c-*erm*
JQ184	TTATTTCCTCCCGTTAAATAATAGATAACTAT		
TT195	ATAGTTATCTATTATTTAACGGGAGGAAATAAAAGAAAAAGATTTTATTGTGTGAGGAGC	D39	3' containing downstream *ezrA*
TT330	GAGGAGTTCGGACTCGACTCTCTCCTTCAAGAA		
**For construction of IU14662 (*ht*-L_6-*mapZ*)**			
P1523	GAGGTCTCTATTCTCAAAGATGTGGCAACTGTC	D39	5' fragment including upstream of *mapZ*
AJP402	AAATGGAAAACCAGTACCAATTTCAGCCATGAGTATCCCTTTCTATTTTACCTCTTGCCG		
YT101	ATGGCTGAAATTGGTACTGGTTTTCCATTT	IU13910	*ht*-L_5
YT104	ACCAGAACCTTGACCAGATCCTGGTCCTTG		
AJP403	AGGACCAGGATCTGGTCAAGGTTCTGGTAGTAAAAAAAGACGAAATCGTCATAAAAAAGA	D39	3' fragment including *mapZ-gnd'*
P1526	AATTGCATATCACCGTACTCAATACCATTGTG		
**For construction of IU14738 (*iht*-L_6-*mapZ*)**			
P1523	GAGGTCTCTATTCTCAAAGATGTGGCAACTGTC	D39	5' fragment including upstream of *mapZ* including i-tag
AJP404	AGAAGCCAAAAAAATTTCCAAACCTTTTTTATCCATGAGTATCCCTTTCTATTTTACCTC		
AJP405	GATAAAAAAGGTTTGGAAATTTTTTTGGCTTCTGCTGAAATTGGTACTGGTTTTCCATTT	IU14662	3' fragment including i-tag-*ht*-L_6-*mapZ-gnd'*
P1526	AATTGCATATCACCGTACTCAATACCATTGTG		
**For construction of IU14850 (*bgaA'*::*tet*-P_{Zn}-*ftsZ*(D214A))**			
TT657	CGCCCCAAGTTCATCACCAATGACATCAAC	IU8122	5' *bgaA'*::*tet*-P_{Zn}-*ftsZ*(D214A) GAT→GCT
AJP406	TGCCATTACCGTTTTCACAGCGGCAAAGTCAAGGTTAATC		
AJP407	GATTAACCTTGACTTTGCCGCTGTGAAAACGGTAATGGCA	IU8122	3' *ftsZ*(D214A) at *bgaA* GAT→GCT
CS121	GCTTTCTTGAGGCAATTCACTTGGTGC		

**For construction of IU14852 (*bgaA'*::*tet*-P_{Zn}-*ftsZ*(D214A)-L_2-*sfgfp*)**			
TT657	CGCCCCAAGTTCATCACCAATGACATCAAC	IU8122	5' *bgaA'*::*tet*-P_{Zn}-*ftsZ*(D214A) GAT→GCT
AJP406	TGCCATTACCGTTTTCACAGCGGCAAAGTCAAGGTTAATC		
AJP407	GATTAACCTTGACTTTGCCGCTGTGAAAACGGTAATGGCA	IU13315	3' *ftsZ*(D214A)-L_2-*sfgfp* at *bgaA* GAT→GCT
CS121	GCTTTCTTGAGGCAATTCACTTGGTGC		
**For construction of IU14927 (*iht*-L_6-*pbp2x*)**			
TT713	CGCCACTGGACATGCGGATGAATCAGGATG	IU13910	5' fragment containing *i-tag* and upstream of *pbp2x*
AJP188	AAAAAATTTCCAAACCTTTTTATCCATATCTTACTCCGCTATTCTAATATTTTCATTGT		
AJP405	GATAAAAAAGGTTTGGAAATTTTTTGGCTTCTGCTGAAATTGGTACTGGTTTTCCATTT	IU13910	3' fragment containing *i-tag*-*ht*-L_6-*pbp2x*
TT717	GTCGGTCGTTGCGTTGTTGCCAGAATTTCC		
**For construction of IU15096 (*ftsW*-L_0-*ht*-P_c-*erm*)**			
TT482	GTTGGATTTCGGTTGCAGGAGTAACTATTCAGCCA	D39	5' containing *ftsW*-L_0
JQ182	GCCAGAACCAGCAGCGGAGCCAGCGGAACCCTTCAACAGAAGGTTCATTGGTTGATTTTC		
JQ179	GGTTCCGCTGGCTCCGCTGCTGGTTCTGGC	IU14290	middle containing L_0-*ht*-P_c-*erm*
JQ184	TTATTTCCTCCCGTTAAATAATAGATAACTAT		
JQ183	ATAGTTATCTATTATTTAACGGGAGGAAATAAGATAAAGAAAGGATAGTTTATGTCTCTT	D39	3' containing downstream of *ftsW*
TT483	TTCCATCACGGTCACCACCTATCCACATAC		
**For construction of IU15231 (Δ*pbp2x*::P_c-[*kan-rpsL*$^+$])**			
TT713	CGCCACTGGACATGCGGATGAATCAGGATG	D39	5' fragment containing 54 5' nt of *pbp2x*
TT1157	CATTATCCATTAAAAATCAAACGGATCCTACGGCGATTTCCGATTTTTGGTCGC		
Kan rpsL forward	TAGGATCCGTTTGATTTTTAATGGATAATG	P_c-[*kan-rpsL*$^+$] cassette	P_c-[*kan-rpsL*$^+$]
Kan rpsL reverse	GGGCCCCTTTCCTTATGCTTTTG		

TT1158	CAAAAGCATAAGGAAAGGGGCCCGTTC GTGCTAACACAGCTATCAAGGAC	D39	3’ flanking fragment containing 57 3’ nt of *pbp2x*
TT346	AGAAGTCAACCTTCCACTCGCTCCAAGG AT		
**For construction of IU15246 (*pbp2x*(S337A))**			
TT713	CGCCACTGGACATGCGGATGAATCAGG ATG	D39	5’ fragment containing point mutation (TCC→GCC)
TT1159	CATCACTTTCATAGTGGCACCTGGCTCA TAGTTACTTTGG		
TT1160	CCAAAGTAACTATGAGCCAGGTGCCACT ATGAAAGTGATG	D39	3’ fragment containing point mutation (TCC→GCC)
TT346	AGAAGTCAACCTTCCACTCGCTCCAAGG AT		
**For construction of IU15599 (*iht*-L$_6$-*ftsZ*)**			
TT457	ATTGTGGATGGTTTCCAAGGGATTCGTG AC	D39	5’ containing upstream of *ftsZ*$^+$ and *i-tag*
AJP450	AGCCAAAAAAATTTCCAAACCTTTTTTAT CCATAATTTATTTTTCCTCTTTATTCGTCA A		
AJP405	GATAAAAAAGGTTTGGAAATTTTTTTGGC TTCTGCTGAAATTGGTACTGGTTTTCCAT TT	IU14738	middle containing *i-tag-ht*-L$_6$
YT104	ACCAGAACCTTGACCAGATCCTGGTCCT TG		
AJP451	CAAGGACCAGGATCTGGTCAAGGTTCTG GTACATTTTCATTTGATACAGCTGCTGCT CAA	D39	3’ fragment containing L$_6$-*ftsZ*
TT462	CAGATGTTCACTCCTTGACCTGCTGCCT GG		
**For construction of K767 (Δ*murA1*::P$_c$-[*kan-rpsL*$^+$])**			
P1554	GATTTTGTGGTACGACGGGCATGTATAG CG	D39	5’ fragment with 60 bp of 5’ *murA1*
P1556	CATTATCCATTAAAAATCAAACGGATCCT AACCACTAATAGTGATTTCACCTTGCAGT GG		
Kan rpsL forward	TAGGATCCGTTTGATTTTTAATGGATAAT G	P$_c$-[*kan-rpsL*$^+$] cassette	P$_c$-[*kan-rpsL*$^+$]
Kan rpsL reverse	GGGCCCCTTTCCTTATGCTTTTG		
P1557	AAACGTCCAAAAGCATAAGGAAAGGGG CCCTCTGATATTATCGAAAAATTACGTAA TTTA	D39	3’ fragment with 60 bp of 3’ *murA1*

P1555	TGAACCTGAAATCCCCCTGTAACCAGAA CT		

[a]Strains were constructed as described SI Appendix, Experimental Procedures.

[b]Amino-acid linkers are annotated as: L_0 (GSAGSAAGSG) (5, 6); L_1 (LEGSG) in *gfp*-L_1-*mapZ*; L_2 (KLDIEFLQ) in *ftsZ*-L_2-*gfp* (7); L_5 (SGSGGEAAAKAG) in *ftsZ*-L_5-*cfp* (8); L_6 (LEGSGQGPGSGQGSG) encoded by codons optimized for *Spn*; L_8 (LEGSGQGPGSGQGSG) encoded by codons not optimized for *Spn* (9).

[c]Antibiotic resistance markers: Erm^R, erythromycin; Kan^R, kanamycin; Spc^R, spectinomycin; Str^R, streptomycin; Cm^R, chloramphenicol; Tet^R, tetracycline.

[d]All strains containing zinc-inducible promoter (P_{czcD} annotated here as P_{Zn}) contain the 24 bp upstream of *ftsA*$^+$ prior to the start codon (4).

[e]MK458 genomic DNA containing *ftsZ*-L_5-*cfp* was provided by the Veening laboratory (8). *ftsZ*-L_5-*cfp* was amplified using primers TT165-TT166 and sequenced. JWV500 amplicon was also from the Veening laboratory (10).

[f]For IU8124, IU12406, and IU14355 parent strains were transformed with the indicated amplicon and were plated on TSAII-BA plates containing the appropriate antibiotic and added 0.5 mM $ZnCl_2$ and 0.05 mM $MnSO_4$ (+Zn). Growth of resulting transformants was confirmed to be zinc dependent.

[g]Construction of temperature-sensitive mutant, *ftsZ*(G107S). Merodiploid strains IU10612 (*rpsL*$^+$ *ftsZ*(G107S)//P_{Zn}-*ftsZ*$^+$), IU14375 (*rpsL1 ftsZ*(G107S)//P_{Zn}-*ftsZ-sfgfp*) and IU14377 (*rpsL1 ftsZ*(G107S)//P_{Zn}-*ftsZ*$^+$) were constructed in parent Δ*ftsZ* depletion strains, IU8124, IU14355, and IU12406, respectively. The parent strains were grown from glycerol stocks in BHI broth with added 0.5 mM $ZnCl_2$ and 0.05 mM $MnSO_4$ (+Zn)

at 37° C in 5% CO_2. An amplicon containing the *ftsZ*(G107S) allele was transformed into each parent strain and plated on TSAII-BA plates lacking antibiotic or Zn/Mn at 32° C. Transformants (4 of 4) showed growth on patch plates at 32° C, but not at 42° C, indicative of the *ftsZ*(G107S) temperature-sensitive allele. Replacement of Δ*ftsZ*::*aad9* by *ftsZ*(G107S) was confirmed by spectinomycin sensitivity at 32° C, by PCR analysis of mutants grown in BHI broth at 32° C, and by sequencing of *ftsZ* in strain IU10612. For construction of IU14408 and IU14508, zinc + manganese were added during all steps of strain construction to mitigate reversion of *ftsZ*(G107S) to *ftsZ*$^+$. Temperature sensitivity of strain IU14508 was confirmed by patching at 42° C on plates lacking Zn/Mn, resulting in no colonies, whereas growth occurred in liquid cultures lacking Zn/Mn at 32° C and 37° (e.g., SI Appendix, Fig. S22A).

[h]Genomic DNA (gDNA) of the following *Spn* strains was used as templates for PCR reactions: D39 from IU1690 (3); IU4888 (D39 Δ*cps* Δ*gpsB*<>*aad9*//*bgaA*'::P_{fcsK}-*gpsB*$^+$) (11); IU6397 (D39 *rpsL1* Δ*phoU2* *bgaA*'::*kan*-t1t2-P_{ftsA}-*phoU2*$^+$) (12); superfolder-GFP (*sfgfp*) (10); and IU3966 (D39 *bgaA*'::*tet*-P_{Zn}-GFP-L-*divIVA*), templated from pJWV25 (13), pUC57-*gfp*(*Sp*) (14).

[i]P_c-*erm* and P_c-[*kan*-*rpsL*$^+$] cassettes are described in (15).

[j]*halotag(ht)*-L_6 codon optimized for *Spn* has the following sequence:

ATGGCTGAAATTGGTACTGGTTTTCCATTTGATCCACATTATGTTGAAGTTTTGGGT
GAACGTATGCATTATGTTGATGTTGGTCCACGTGATGGTACTCCAGTTTTGTTTTTG
CATGGTAATCCAACAAGTTCTTATGTTTGGCGAAATATTATTCCACATGTTGCTCCA
ACACATCGTTGTATTGCTCCAGATTTGATTGGTATGGGTAAATCTGATAAACCAGAT
TTGGGTTATTTTTTTGATGATCATGTTCGTTTTATGGATGCTTTTATTGAAGCTTTGG

GTTTGGAAGAAGTTGTTTTGGTTATTCATGATTGGGGTTCTGCTTTGGGTTTTCATTGGGCTAAACGTAATCCAGAACGTGTTAAAGGTATTGCTTTTATGGAATTTATTCGTCCAATTCCAACATGGGATGAATGGCCAGAATTTGCTCGTGAAACATTTCAAGCTTTTCGTACAACAGATGTTGGTCGTAAATTGATTATTGATCAAAATGTTTTTATTGAAGGTACATTGCCAATGGGTGTTGTTCGTCCATTGACAGAAGTTGAAATGGATCATTATCGTGAACCATTTTTGAATCCAGTTGATCGTGAACCATTGTGGCGTTTTCCAAATGAATTGCCAATCGCTGGTGAACCAGCTAATATTGTTGCTTTGGTTGAAGAATATATGGATTGGTTGCATCAATCTCCAGTTCCAAAATTGTTGTTTTGGGGTACACCAGGTGTTTTGATTCCACCAGCTGAAGCTGCTCGTTTGGCTAAATCTTTGCCAAATTGTAAAGCTGTTGATATTGGTCCAGGTTTGAATTTGTTGCAAGAAGATAATCCAGATTTGATTGGTTCTGAAATTGCTCGTTGGTTGTCTACATTGGAAATTTCTGGTTTGGAAGGATCAGGACAAGGACCAGGATCTGGTCAAGGTTCTGGT

[k]YB7656 is *B. subtilis* PY79 *ftsZ-mneongreen* and was provided by the Brun laboratory (9).

[l]*i* in *isfgfp* or *iht* refers to an *i-tag* sequence. The i-tag used consists of the first 11 amino acids of WchA plus an additional serine, MDKKGLEIFLA(S), encoded by bases ATGGATAAAAAAGGTTTGGAAATTTTTTTGGCT(TCT). These amino acids enhance expression of N-terminal fusion proteins in Gram-positive bacteria (16). Note, we have added an additional serine at the C-terminus of the i-tag to assist with flexibility linking to *sfgfp* or *ht* (isfGFP or iHT, respectively). There is no additional methionine after the i-tag.

[m]IU14059, IU15231, IU15246 parent strains were transformed with the indicated amplicon and were plated on TSAII-BA plates containing the appropriate antibiotic and

added 0.4 mM $ZnCl_2$ and 0.04 mM $MnSO_4$ (+Zn). Growth of resulting transformants was confirmed to be zinc dependent.

References cited in SI Appendix, Table S1

1. Lanie JA*, et al.* (2007) Genome sequence of Avery's virulent serotype 2 strain D39 of *Streptococcus pneumoniae* and comparison with that of unencapsulated laboratory strain R6. *J Bacteriol* 189(1):38-51.
2. Rued BE*, et al.* (2017) Suppression and synthetic-lethal genetic relationships of Δ*gpsB* mutations indicate that GpsB mediates protein phosphorylation and penicillin-binding protein interactions in *Streptococcus pneumoniae* D39. *Mol Microbiol* 103(6):931-957.
3. Tsui HT*, et al.* (2016) Suppression of a Deletion Mutation in the Gene Encoding Essential PBP2b Reveals a New Lytic Transglycosylase Involved in Peripheral Peptidoglycan Synthesis in *Streptococcus pneumoniae* D39. *Mol Microbiol 100*(6), 1039-1065.
4. Mura A*, et al.* (2016) Roles of the essential protein FtsA in cell growth and division in *Streptococcus pneumoniae*. *J Bacteriol 199*(3), e00608-16.
5. Waldo GS, Standish BM, Berendzen J, & Terwilliger TC (1999) Rapid protein-folding assay using green fluorescent protein. *Nat Biotech* 17(7):691-695.
6. Wayne KJ*, et al.* (2010) Localization and cellular amounts of the WalRKJ (VicRKX) two-component regulatory system proteins in serotype 2 *Streptococcus pneumoniae*. *J Bacteriol* 192(17):4388-4394.
7. Fleurie A*, et al.* (2014) Interplay of the serine/threonine-kinase StkP and the paralogs DivIVA and GpsB in pneumococcal cell elongation and division. *PLoS Genet* 10(4):e1004275.
8. van Raaphorst R, Kjos M, & Veening JW (2017) Chromosome segregation drives division site selection in *Streptococcus pneumoniae*. *Proc Natl Acad Sci USA* 114(29):E5959-E5968.
9. Bisson-Filho AW*, et al.* (2017) Treadmilling by FtsZ filaments drives peptidoglycan synthesis and bacterial cell division. *Science* 355(6326):739-743.
10. Kjos M*, et al.* (2015) Bright fluorescent *Streptococcus pneumoniae* for live-cell imaging of host-pathogen interactions. *J Bacteriol* 197(5):807-818.
11. Land AD*, et al.* (2013) Requirement of essential Pbp2x and GpsB for septal ring closure in *Streptococcus pneumoniae* D39. *Mol Microbiol* 90(5):939-955.
12. Zheng JJ, Sinha D, Wayne KJ, & Winkler ME (2016) Physiological Roles of the Dual Phosphate Transporter Systems in Low and High Phosphate Conditions and in Capsule Maintenance of *Streptococcus pneumoniae* D39. *Front Cell Infect Microbiol* 6:63.
13. Eberhardt A, Wu LJ, Errington J, Vollmer W, & Veening JW (2009) Cellular localization of choline-utilization proteins in *Streptococcus pneumoniae* using novel fluorescent reporter systems. *Mol Microbiol* 74(2):395-408.
14. Martin B*, et al.* (2010) Expression and maintenance of ComD-ComE, the two-component signal-transduction system that controls competence of *Streptococcus pneumoniae*. *Mol Microbiol* 75(6):1513-1528.
15. Tsui HC, Keen SK, Sham LT, Wayne KJ, & Winkler ME (2011) Dynamic distribution of the SecA and SecY translocase subunits and septal localization of the HtrA surface chaperone/protease during *Streptococcus pneumoniae* D39 cell division. *mBio* 2(5): pii: e00202-11.

16. Catalao MJ, Figueiredo J, Henriques MX, Gomes JP, & Filipe SR (2014) Optimization of fluorescent tools for cell biology studies in Gram-positive bacteria. *PloS One* 9(12):e113796.
17. Boersma MJ*, et al.* (2015) Minimal Peptidoglycan (PG) Turnover in Wild-Type and PG Hydrolase and Cell Division Mutants of Streptococcus pneumoniae D39 Growing Planktonically and in Host-Relevant Biofilms. *J Bacteriol* 197(21):3472-3485.
18. Tsui, HC, *et al.* (2014). Pbp2x localizes separately from Pbp2b and other peptidoglycan synthesis proteins during later stages of cell division of *Streptococcus pneumoniae* D39. *Mol Microbiol 94*(1), 21-40.

SI APPENDIX, FIGURES AND LEGENDS

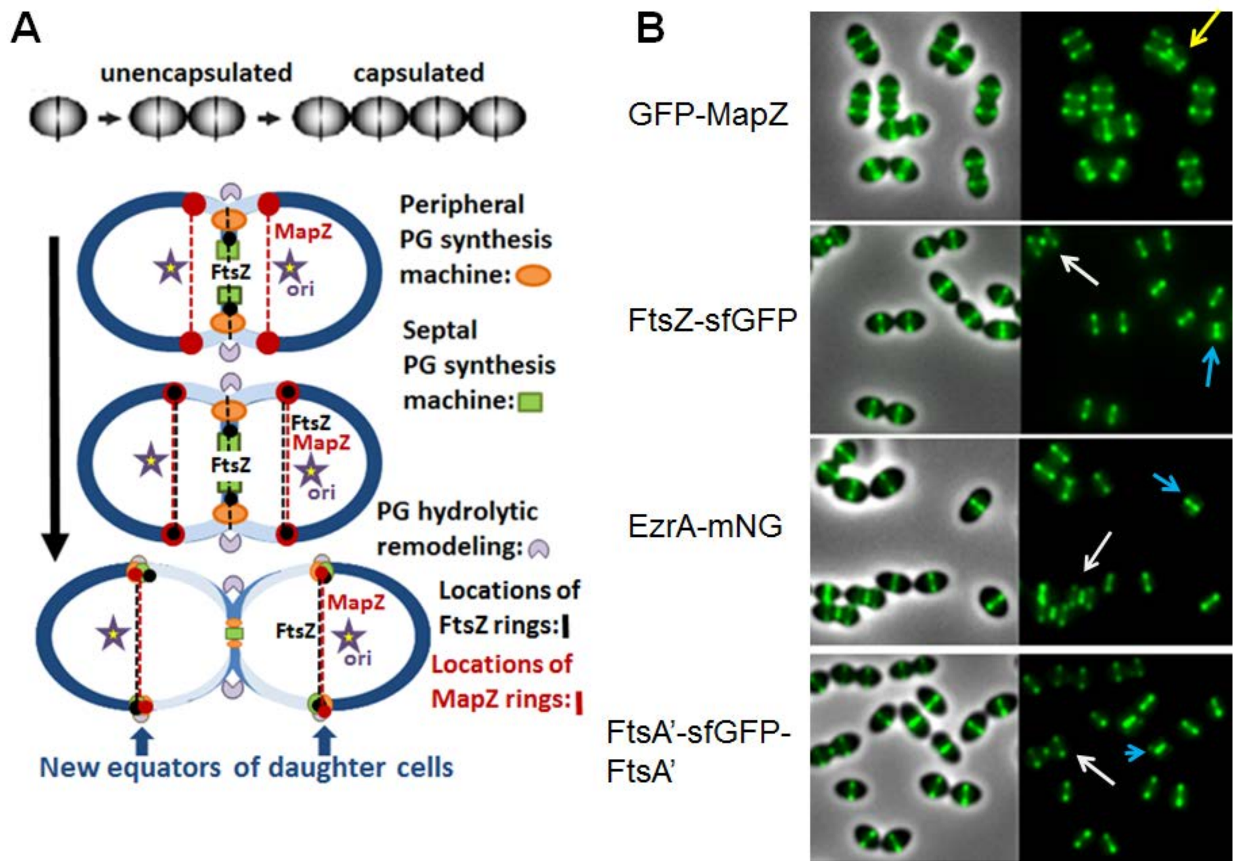

Fig S1. Locations of FtsZ, MapZ, *ori* (origin of chromosomal replication), and septal and peripheral PG synthesis during stages of the *Spn* division cycle. (A) Top, division of *Spn* cells perpendicular to the long axis of cells, resulting in short chains of cells, especially in encapsulated strains. Bottom, new equators of daughter cells become the septa in the next round of division, where a limited number of division stages are shown. Positions of the septal PG synthesis machine containing bPBP2x and peripheral (side-wall like) PG synthesis machine containing bPBP2b are indicated. Newly synthesized peripheral or septal PG is shown as light or medium blue, respectively, while old PG is shown as dark blue. See text for descriptions. (B) 2D-epifluorescence microscopy of MapZ, FtsZ, EzrA, and FtsA from Fig. S2C marked for

features depicted in (A), including splitting of MapZ rings in early divisional cells (yellow arrow); the presence of three rings of FtsZ, EzrA, and FtsA in middle-to-late divisional cells (white arrows); and detection by conventional wide-field microscopy of a nascent FtsZ ring adjacent to a mature septal ring in an early divisional cell (blue arrows).

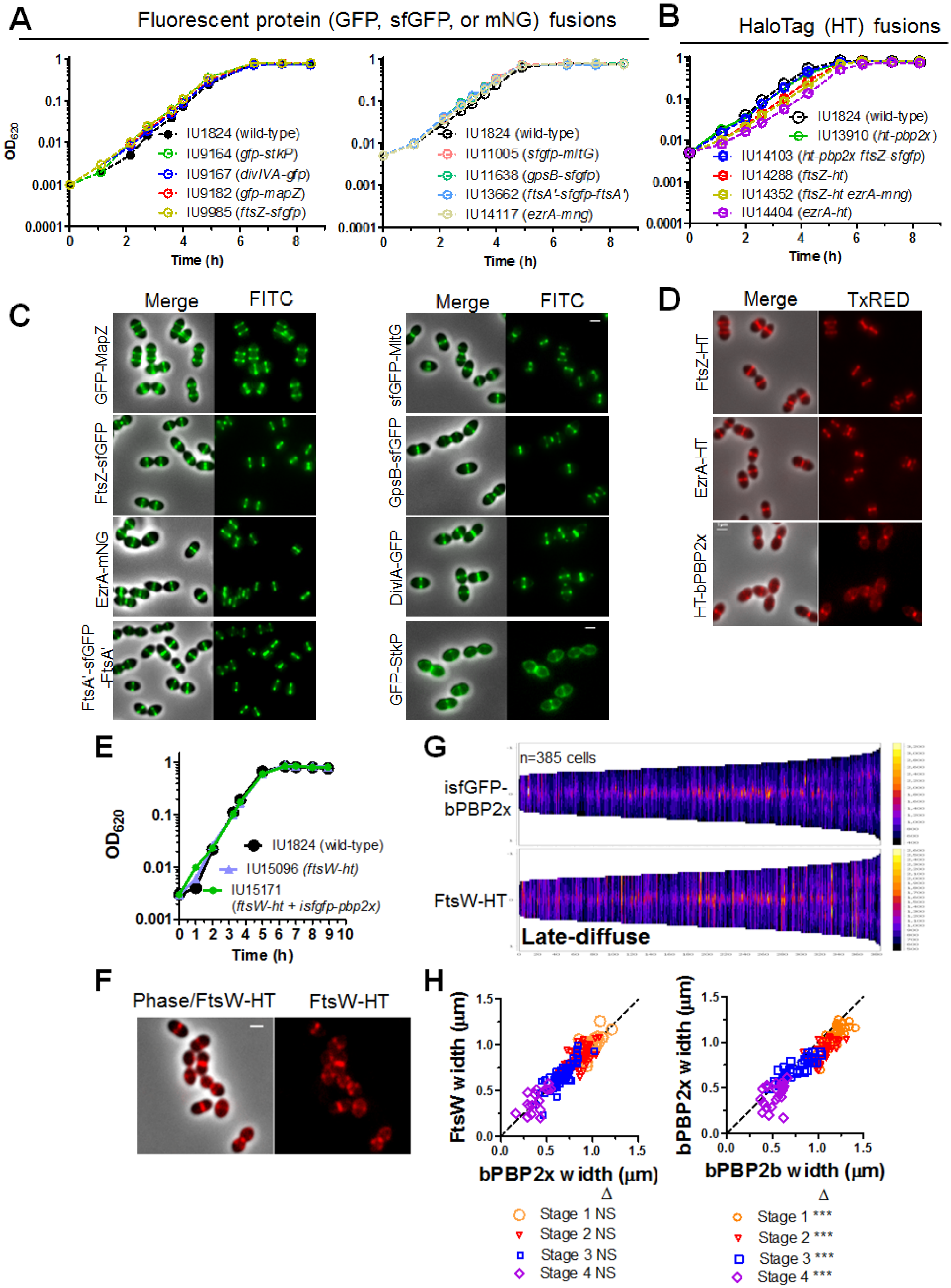
A
Fluorescent protein (GFP, sfGFP, or mNG) fusions
B
HaloTag (HT) fusions
OD620
Time (h)
IU1824 (wild-type)
IU9164 (gfp-stkP)
IU9167 (divIVA-gfp)
IU9182 (gfp-mapZ)
IU9985 (ftsZ-sfgfp)
IU11005 (sfgfp-mltG)
IU11638 (gpsB-sfgfp)
IU13662 (ftsA'-sfgfp-ftsA')
IU14117 (ezrA-mng)
IU13910 (ht-pbp2x)
IU14103 (ht-pbp2x ftsZ-sfgfp)
IU14288 (ftsZ-ht)
IU14352 (ftsZ-ht ezrA-mng)
IU14404 (ezrA-ht)
C
Merge
FITC
GFP-MapZ
FtsZ-sfGFP
EzrA-mNG
FtsA'-sfGFP -FtsA'
sfGFP-MltG
GpsB-sfGFP
DivIVA-GFP
GFP-StkP
D
TxRED
FtsZ-HT
EzrA-HT
HT-bPBP2x
E
IU15096 (ftsW-ht)
IU15171 (ftsW-ht + isfgfp-pbp2x)
G
n=385 cells
isfGFP-bPBP2x
FtsW-HT
Late-diffuse
F
Phase/FtsW-HT
FtsW-HT
H
FtsW width (μm)
bPBP2x width (μm)
bPBP2b width (μm)
Δ
Stage 1 NS
Stage 2 NS
Stage 3 NS
Stage 4 NS
Stage 1 ***
Stage 2 ***
Stage 3 ***
Stage 4 ***

538

Fig. S2. Fusion proteins are functional in strains grown in C+Y pH 6.9 at 37° C in 5% CO_2. Strains expressing GFP, super-folder GFP (sfGFP), mNeonGreen, or HaloTag (HT) fusions, and the untagged parent were grown in BHI broth to OD_{620} = 0.1-0.4, washed once in C+Y pH 7.1, and diluted into C+Y, pH 7.1 to OD_{620} = 0.001-0.003 to start a new culture that was grown at 37° C (in CO_2). Growth was monitored by OD_{620} at the indicated times. Experiments were performed 2-4 times independently, and representative data are shown. (A), (B), and (E), growth curves. The average doubling time of the wild-type parent (IU1824) is 32.8 ± 2.0 min (SD) from four independent biological replicates. (C) Phase-contrast and epifluorescence (FITC filter) microscopy was performed at OD_{620} = 0.08-0.18 on cells that were concentrated 200X by centrifugation in C+Y, pH 6.9 immediately before viewing. For (D), (F), (G), and (H), 500 μL of culture at OD_{620} = 0.08-0.18 were incubated with HT-TMR ligand (5 μM final concentration) for 15 min at 37° C. Cells were washed twice in C+Y, pH 7.1, concentrated by centrifugation, and imaged by phase-contrast and epifluorescence microscopy (TX-Red-filter). Scale bar = 1 μm. (G) Demograph of strain IU15171 expressing FtsW-HT (labeled with HT-TMR ligand) and isfGFP-bPBP2x from two independent biological replicates. See SI Appendix, Experimental Procedures for additional details. (H) Paired-width analysis of septal rings of FtsW and bPBP2x (left graph; IU15171) or bPBP2x and bPBP2b (right graph; IU15068) from 2D-epifluorescence micrographs. Width measurements and plotting were done using the MATLAB IMA-GUI program (see SI Appendix, Experimental Procedures and (8)). Statistical analysis was performed as described previously (8). NS, not significant; *** P<0.001.

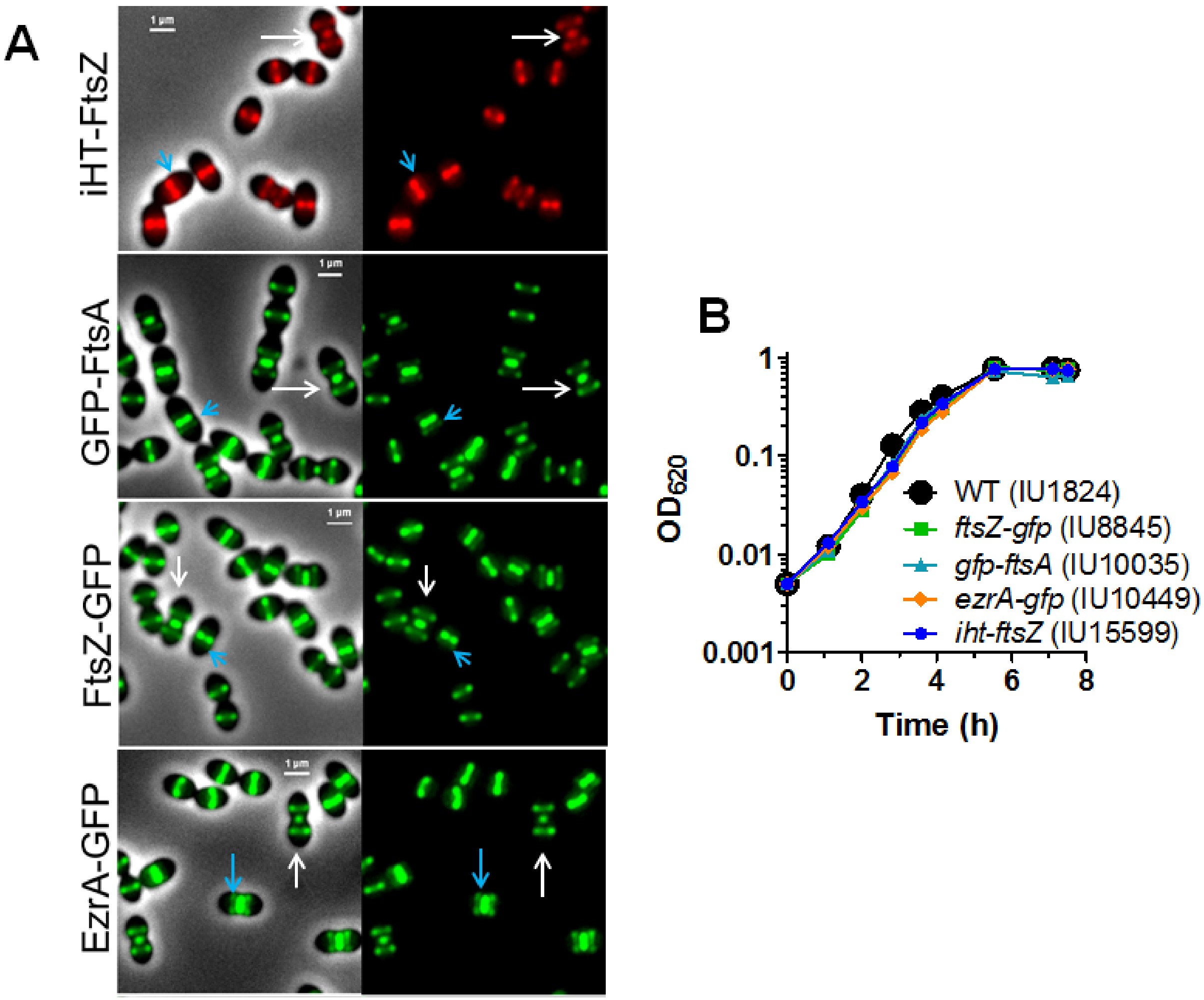

Fig. S3. Fusion proteins are functional in strains grown in C+Y pH 6.9 at 37° C in 5% CO_2. Strains expressing GFP or HaloTag (HT) fusions (*ftsZ-gfp, gfp-ftsA, ezrA-gfp, iht-ftsZ*), and the untagged parent were grown in BHI broth to OD_{620} = 0.1-0.4, washed once in C+Y, pH 7.1, and diluted into C+Y, pH 7.1 to OD_{620} = 0.001-0.003 to start a new culture that was grown at 37° C (in CO_2). Experiments were performed twice independently, and representative data are shown. (A) Cells were obtained for microscopy at OD_{620} ≈ 0.1-0.2, concentrated by centrifugation, and imaged by phase-contrast and epifluorescence microscopy (FITC filter). Phase-contrast images merged with epifluorescence images (left column) or epifluorescence images alone (right

column) of the indicated fusion construct. Blue arrows point to cells with nascent rings, while white arrows point to cells with a three-ring pattern. To visualize iHT-FtsZ, IU15599 was labeled with 500 nm HT-JF549 ligand, washed twice in C+Y, pH 7.1, concentrated by centrifugation, and imaged by phase-contrast and epifluorescence microscopy (TX-Red-filter). (B) Growth was monitored by OD_{620} at the indicated times. Genotypes with corresponding strain numbers are listed.

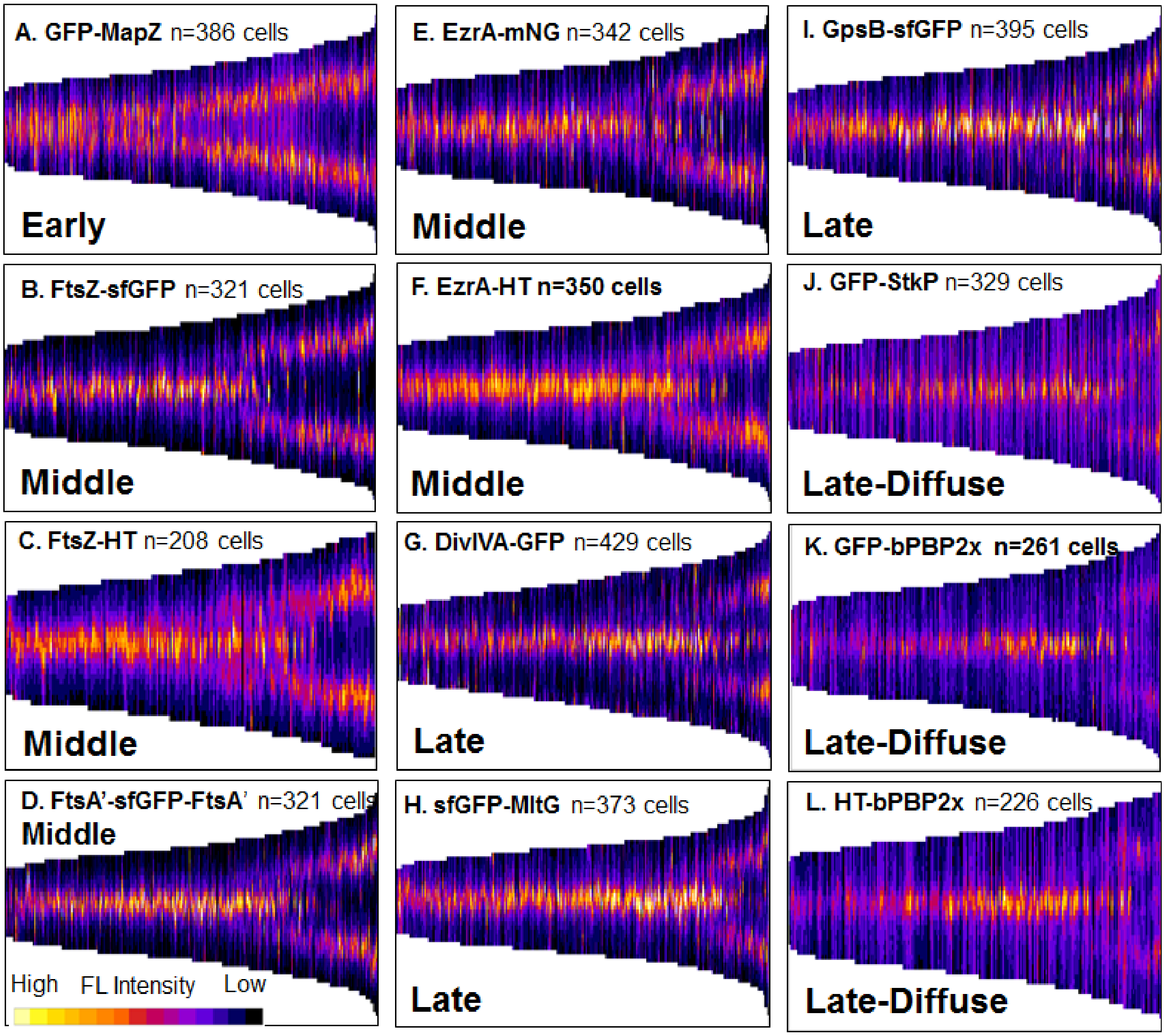

Fig. S4. Relocation of cell division and PG synthesis proteins occurs in three stages in *Spn*. Strains expressing functional fluorescent- or HT-protein fusions expressed from native loci (see SI Appendix, Experimental Procedures and Table S1; note that linkers have been omitted here to simplify nomenclature) were grown in C+Y, pH 6.9 at 37° C in 5% CO_2 to OD_{620} = 0.1-0.2, imaged, and processed by MicrobeJ to generate demographs as described in SI Appendix, Experimental Procedures. Cells are sorted by length from shorter (left) to longer (right), corresponding to pre-divisional

single cells to late-divisional daughter cells about to separate, respectively. Data are from two independent biological replicates, where the total number of cells (n) is indicated. Early arriving protein: (A) MapZ (IU9182; GFP-MapZ). Middle arriving proteins: (B) FtsZ (IU9985; FtsZ-sfGFP); (C) FtsZ (IU14288; FtsZ-HT); (D) FtsA (IU13662; FtsA'-sfGFP-FtsA'); (E) EzrA (IU14117; EzrA-mNG); and (F) EzrA (IU14404; EzrA-HT). Late arriving proteins: (G) DivIVA (IU9167; DivIVA-GFP), (H) MltG (IU11005; sfGFP-MltG), (I) GpsB (IU11638; GpsB-sfGFP), (J) StkP (IU9164; GFP-StkP), (K) bPBP2x (IU9020; GFP-bPBP2x), and (L) bPBP2x (IU13910; HT-bPBP2x). All HT strains were labeled with HT-TMR ligand (see SI Appendix, Experimental Procedures).

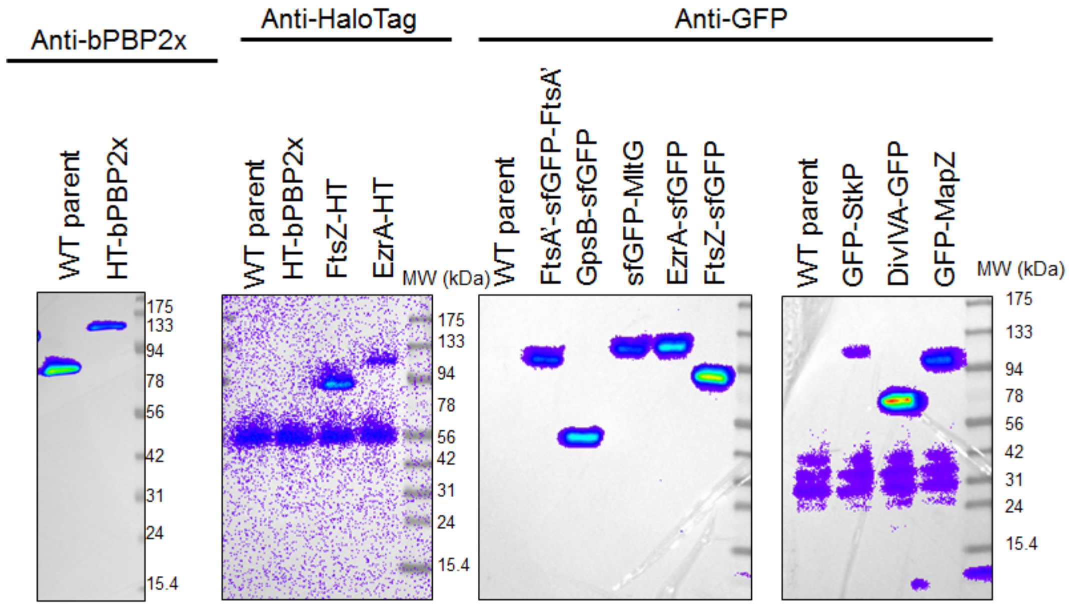

Protein	MW (kDa)
EzrA	67
FtsA	49.5
FtsZ	44.4
DivIVA	20.6
MapZ	51.5
GpsB	12.6
StkP	72.4
bPBP2x	82.6
MltG	60.8
**GFP**	27.1
**sfGFP**	26.7
**Halotag**	33.4

Fig. S5. Western blots of lysates of cells expressing protein fusions. Culture conditions were the same as in Fig. S2. Strains IU1824, IU13910, IU14288, IU14404, IU13662, IU11638, IU11005, IU10254, IU9985, IU9164, IU9167, IU9182 were harvested and lysed with SEDS (SDS, EDTA, Deoxycholate, Salt) buffer as described in SI Appendix, Experimental Procedures, and 3.0 µg of total protein of cell lysate (for WT parent and HT-bPBP2x, left membrane) and 1.0 µg of total protein of cell lysate (WT parent and all other strains, right three membranes) were Western blotted. Predicted protein molecular masses are listed. Experiments were performed twice independently with similar results.

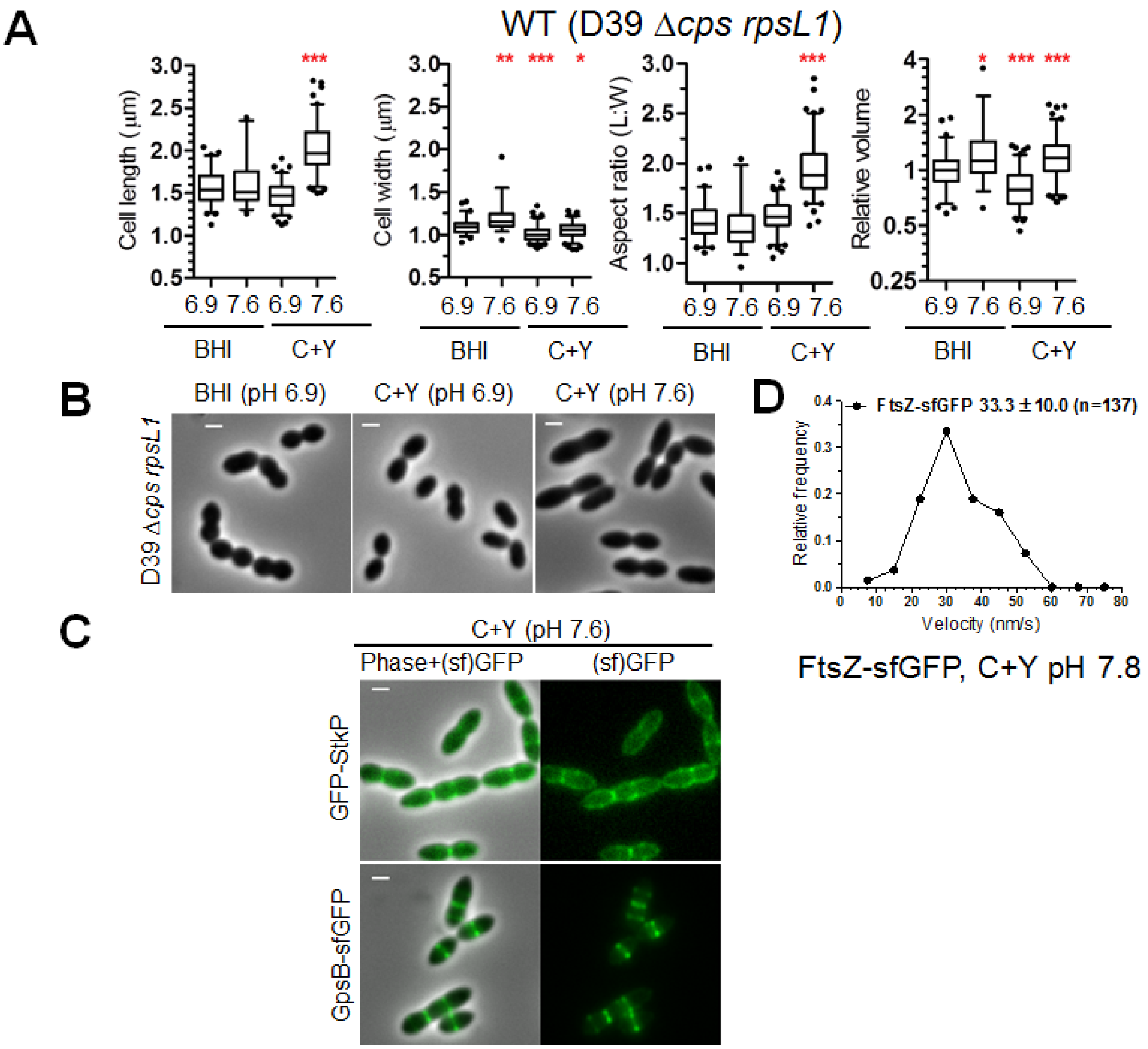

Fig. S6. Effects of pH on *Spn* cell physiology. Wild-type (D39 Δ*cps rpsL1*; IU1824) or strains expressing *gfp-stkP* (IU9164), *gpsB-sfgfp* (IU11638), or *ftsZ-sfgfp* (IU9985) fusions were grown in BHI broth or C+Y at the pH's indicated. Starter cultures were grown in normal BHI broth (pH 6.9 in 5% CO_2) to OD_{620} = 0.1-0.4. For BHI broth cultures, cells were diluted to a starting OD_{620} ≈0.005 in normal BHI broth or BHI adjusted with NaOH to pH 7.6 (in 5% CO_2). For C+Y cultures, BHI-grown starter cells were washed once in C+Y, pH 6.9 and resuspended to a starting OD_{620} ≈0.005 in C+Y, pH 6.9 or C+Y, pH 7.6 in 5% CO_2. Microscopy was performed at OD_{620} = 0.1-0.18, by

concentrating cells 200X by centrifugation in the same media. (A) Box-and-whisker plots (whiskers, 5 and 95 percentile) of cell lengths, widths, aspect ratios, and relative volumes of WT cells (D39 Δ*cps rpsL1*) grown in BHI broth or C+Y at the pH's indicated. P values were obtained by one-way ANOVA analysis (GraphPad Prism, nonparametric Kruskal-Wallis test) compared to cells grown in normal BHI broth, pH 6.9 (in 5% CO_2). P<0.01,**; P<0.001,***. (B) Representative phase-contrast images of WT (D39 Δ*cps*) cells whose measurements are graphed in A. (C) Representative phase-contrast and FITC-filter images of cells expressing *gfp-stkP* or *gpsB-sfgfp* in C+Y pH 7.6 (in 5% CO_2). Data are from 2-3 independent biological replicates. (D) Histogram displaying FtsZ-sfGFP (IU9985) velocities in cells at higher pH (7.8) (see Fig. 1 and SI Appendix, Experimental Procedures) Values are binned in intervals of 7.5 nm/s. Average velocity ± SD are shown from three independent biological replicates.

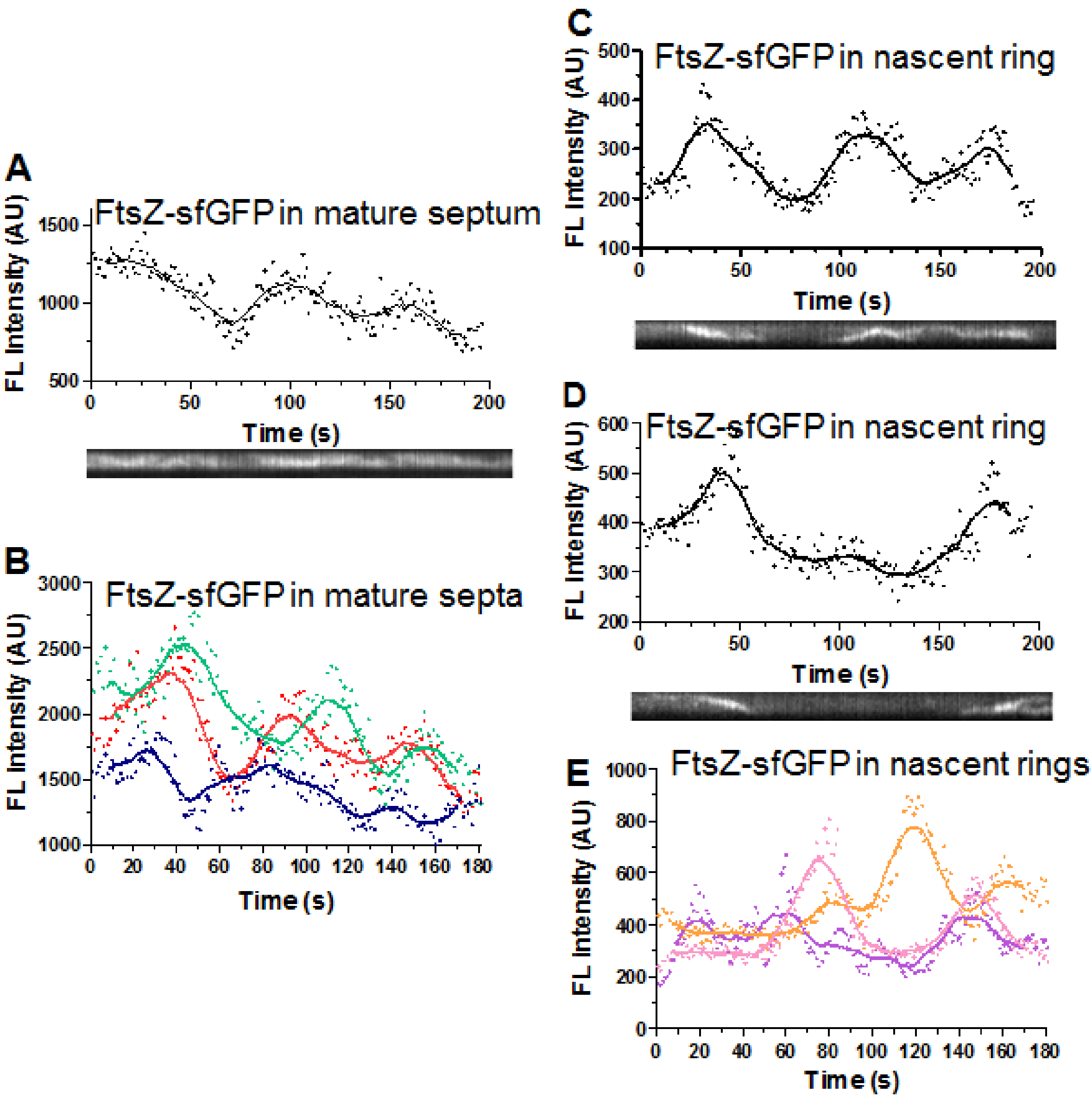

Fig. S7. Large fluctuations of FtsZ-sfGFP intensity in FtsZ rings determined by TIRFm. FtsZ-sfGFP (IU9985) fluorescence was imaged by TIRFm as described in SI Appendix, Experimental Procedures*.* Mean fluorescence intensities (dots) and corresponding moving averages (every 20 points; solid lines) of ring planes are shown as a function of time in 1 s intervals. (A) and (B), fluctuations of intensity of FtsZ-sfGFP in the mature septal rings of strain IU9985. (C-E), fluctuations of intensity of FtsZ-sfGFP

in nascent rings of strain IU9985. (A), (C), and (D) show the corresponding kymographs below each graph. (B) and (E) combine fluctuation traces from several mature-septal and nascent rings, respectively, that are color coded.

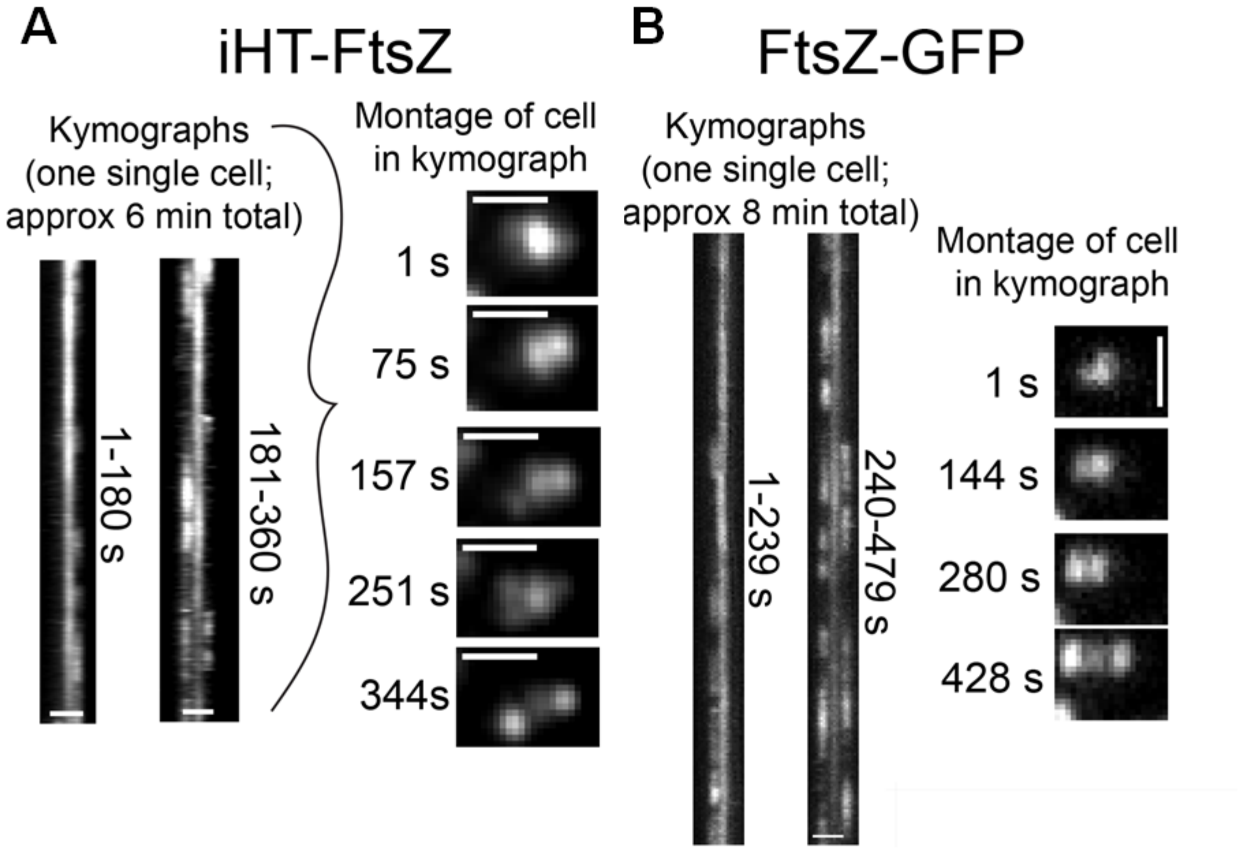

Fig. S8. Additional tags on FtsZ analyzed by TIRFm reveal outward movement of the nascent ring plane containing FtsZ filaments. TIRFm was performed on strains expressing iHT-FtsZ (IU15599, labeled with 500 nm HT-JF549 ligand) or FtsZ-GFP (IU8845) as described in SI Appendix, Experimental Procedures. Cells were imaged for the indicated times (between 6-8 min). Kymographs were drawn across the long axis of cells (line width = 4). Data are from two independent biological replicates. Scale bars = 1.0 µm. Representative analyses are shown of the more than 10 cells analysed per strain.

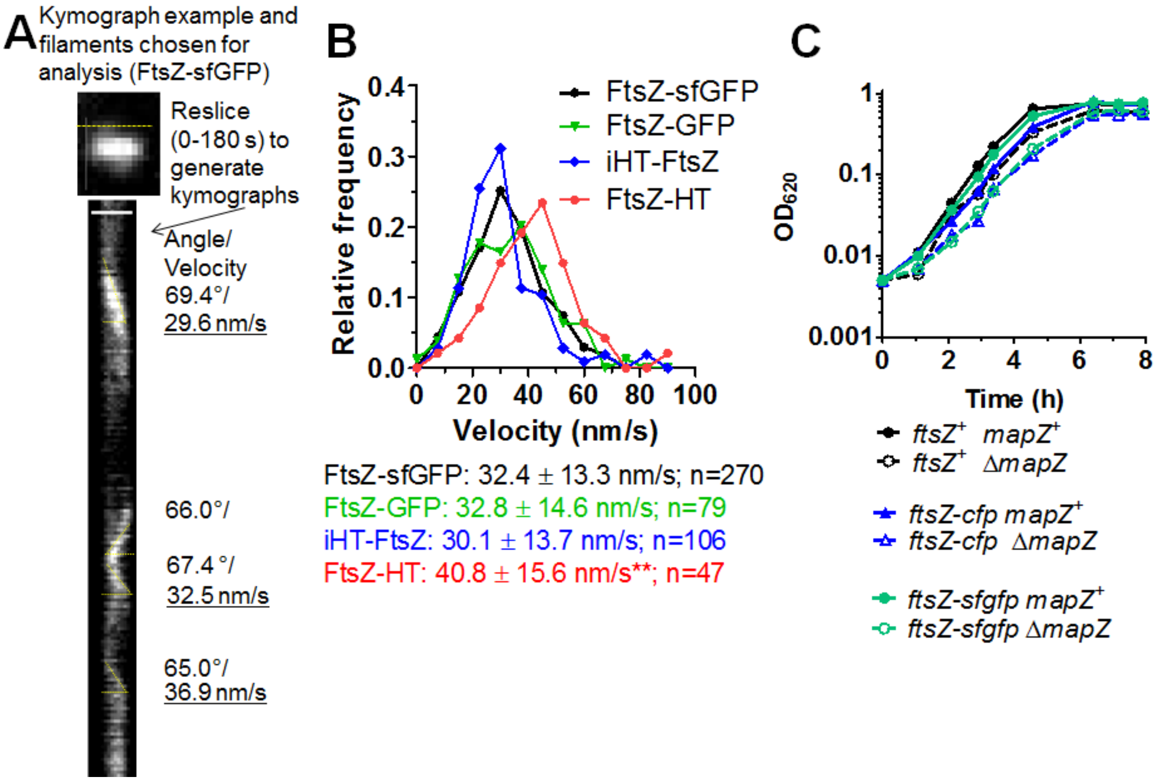

Fig. S9. Velocity determination and functionality of different FtsZ-tagged constructs. Strains were cultured in BHI broth to OD_{620} = 0.1-0.4, washed once in C+Y, pH 7.1, and diluted into C+Y, pH 7.1 to OD_{620} = 0.003-0.005 to start a new culture that was grown at 37° C (in CO_2, final pH = 6.9). (A) Example of the kymograph analysis of movement of FtsZ-sfGFP (IU9985) by TIRFm showing filaments/bundles in a nascent rings (dotted line, top) adjacent and parallel to a mature septal FtsZ ring in an early divisional cell. Kymograph of a 180 s movie (1 frame/s) is shown. Angles were determined and converted to velocity by the equation: velocity = tanΘ/0.079, where Θ is angle in radians. (B) Histogram displaying the velocity distribution of four FtsZ-tagged variants in *Spn* cells determined by TIRFm (FtsZ-sfGFP; IU9985, FtsZ-GFP; IU8845, iHT-FtsZ; IU15599, FtsZ-HT; IU14288). Strains expressing iHT-FtsZ and FtsZ-HT were labeled with 500 nM HT-JF549 ligand. P values relative to FtsZ-sfGFP were obtained by one-way ANOVA analysis (GraphPad Prism, nonparametric Kruskal-Wallis test).

P<0.01, **. Data are from two or more independent biological replicates. (C) Growth curves of strains expressing *ftsZ*$^+$, *ftsZ-cfp*, or *ftsZ-sfgfp* in *mapZ*$^+$ (IU1824, IU13406, IU9985, respectively) or Δ*mapZ* backgrounds (IU9175, IU13408, IU15674, respectively), demonstrate *ftsZ-sfgfp* is functional, resulting in growth similar to previously published functional *ftsZ-cfp* in *mapZ*$^+$ and Δ*mapZ* backgrounds (7). Attempts to introduce Δ*mapZ* to strains expressing iHT-FtsZ and FtsZ-HT were not successful, while a strain expressing FtsZ-GFP in a Δ*mapZ* background (IU9881; *ftsZ-gfp* Δ*mapZ*) yielded small colonies on TSA-II blood agar plates compared to the normal size colonies formed by the strains characterized in Fig. S9C.

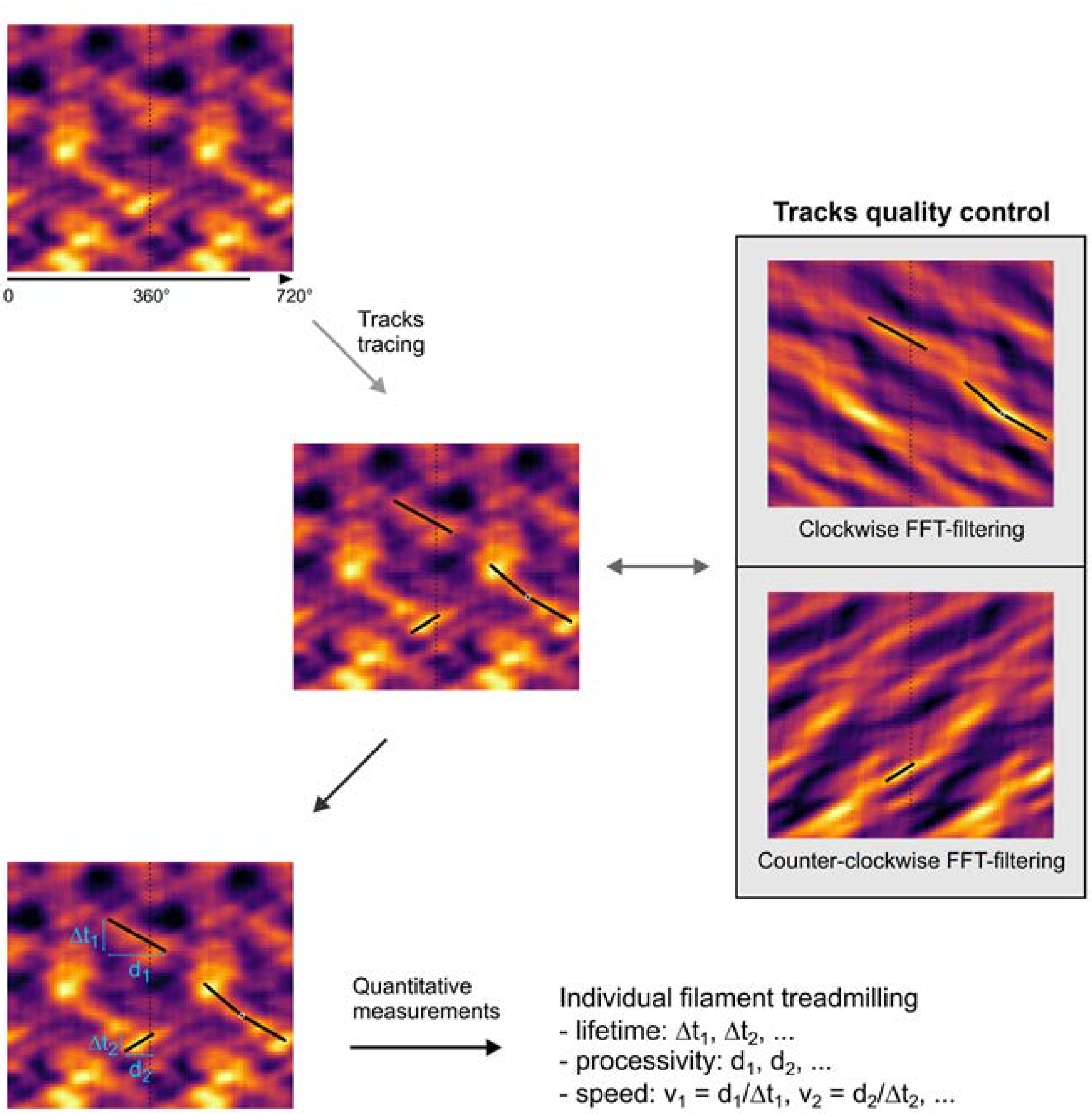

Fig. S10. Method used for the quantitative analysis of individual treadmilling FtsZ-sfGFP filaments in vertically immobilized cells. The dynamics of treadmilling FtsZ-sfGFP filaments (speed, processivity and lifetime) was characterised from kymographs for strain IU9985 (see Movie S3). Tracks were traced on the kymographs using straight or segmented lines in FIJI. For clarity, here only a subset of the tracks traced for each kymograph is displayed. Tracks showing angular (speed) change were traced using segmented lines. Small white circle indicate examples where treadmilling

speed changes on tracks. Kymographs were filtered for clockwise and counter-clockwise motion using FFT filtering to assess the traced tracks quality/accuracy (see SI Appendix, Experimental Procedures). After quality control, quantitative measurements of the traced tracks yielded the value of the lifetime, processivity, and speed for each individual filament.

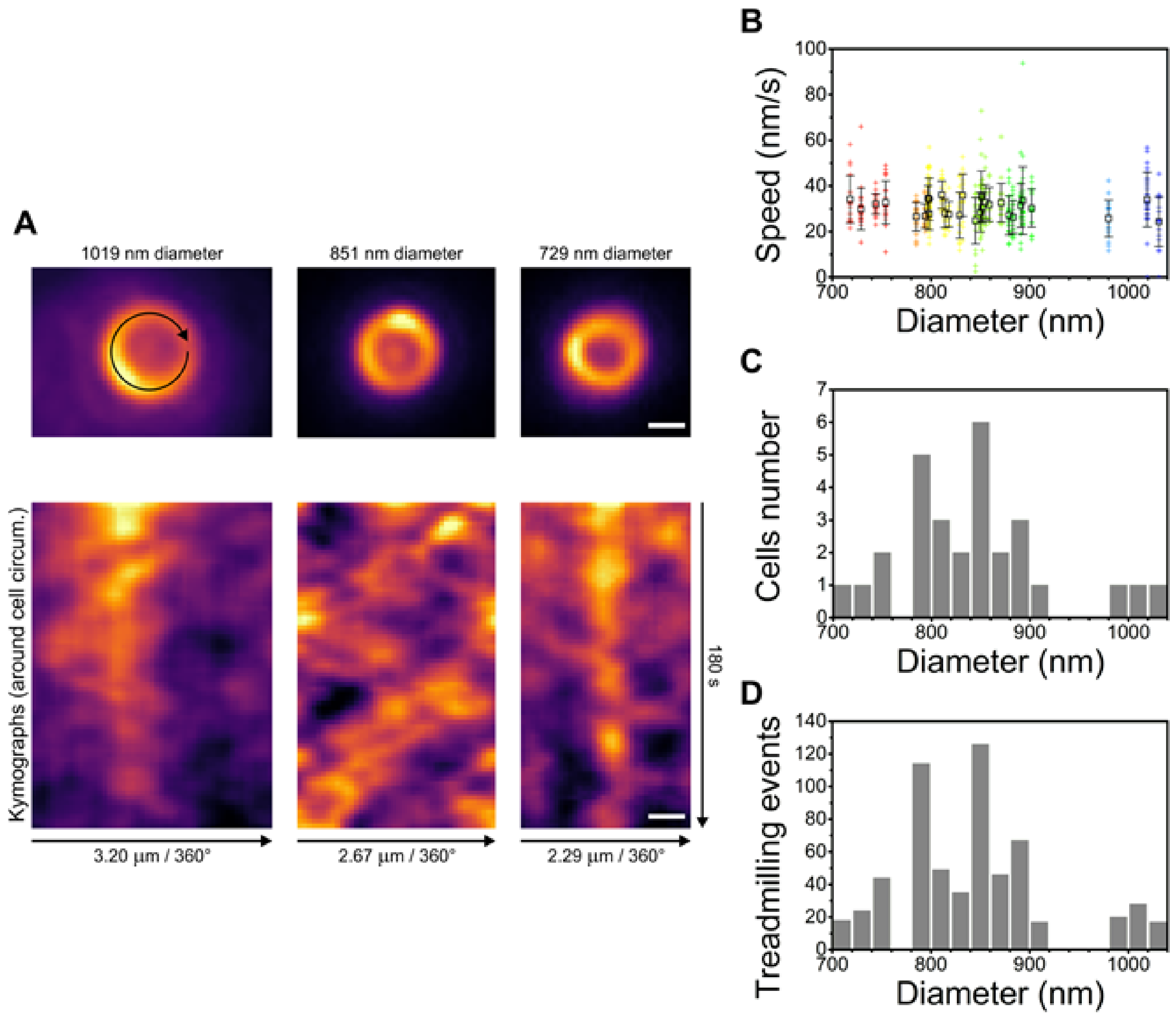

Fig. S11. Treadmilling FtsZ-sfGFP filaments tracks and cell diameters in vertically immobilized cells. (A) Figure shows rings (and kymographs) of cells with diameter spanning the whole range of diameters observed for strain IU9985. (B) FtsZ-sfGFP filament speed shown as a function of cell diameter. Black squares show the mean filament speed in individual cells, where error bars are SDs. Crosses show the individual filament speeds measured for each cell. (C) Number of cells analysed as a function of the cell diameter (n = 29 cells). (D) Number of treadmilling events (filament tracks) analysed for the range of diameters.

A

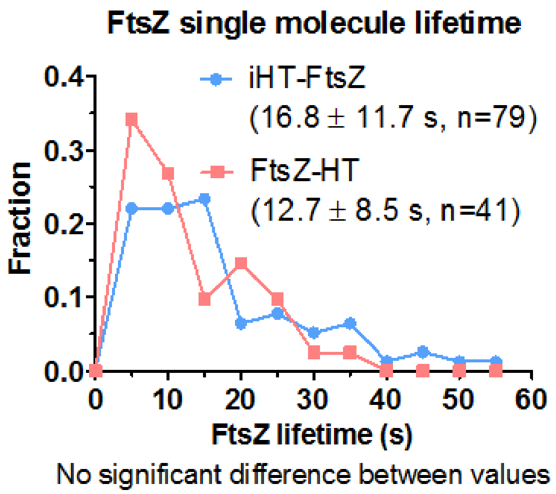

B

Construct	Average Velocity (nm/s)	Average FtsZ Lifetime (s)	Calculated Average FtsZ filament length (nm)
iHT-FtsZ	30.1	16.8	506 ± 420
FtsZ-HT	40.8	12.7	519 ± 399

C

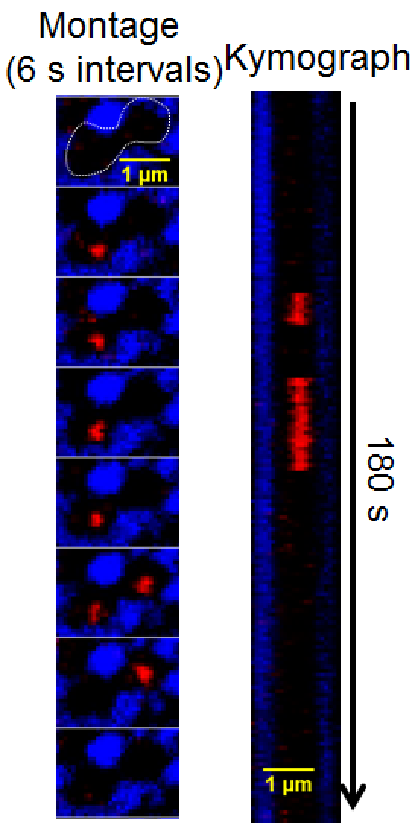

Fig. S12. Lifetimes of static FtsZ single molecules and calculated lengths of FtsZ-filaments. SM-TIRFm was performed on strains expressing iHT-FtsZ (IU15599) or FtsZ-HT (IU14288) labeled with 40 pM of HT-JF549 ligand as described in SI Appendix, Experimental Procedures. Images were obtained at 1 frame/s. Data are from at least two independent biological replicates. (A) Histogram displaying the lifetimes of static FtsZ single molecules in filaments of two different FtsZ-HaloTag constructs. No significant difference in the lifetime of static iHT-FtsZ or FtsZ-HT was indicated by an unpaired two-tailed t-test (GraphPad Prism). (B) Table displaying the average filament velocity of FtsZ HT constructs from SI Appendix, Fig. S9B, the lifetimes of static molecules in filaments from SI Appendix, Fig. S12A, and the FtsZ filament length, calculated by multiplying the average velocity by the average lifetime of the respective FtsZ-HaloTag construct. (C) Representative montage of cells (shown at 6 s intervals)

and kymograph along the equatorial ring plane, where labeled single molecules of iHT-FtsZ are detected. Approximate cell outlines were determined by brightfield microscopy. A cell outline is indicated by a sketched line in the top panel, where cell bodies colored black in the montage. Single molecules of iHT-FtsZ labeled with HT-JF549 ligand appear as red dots. Scale = 1 µm.

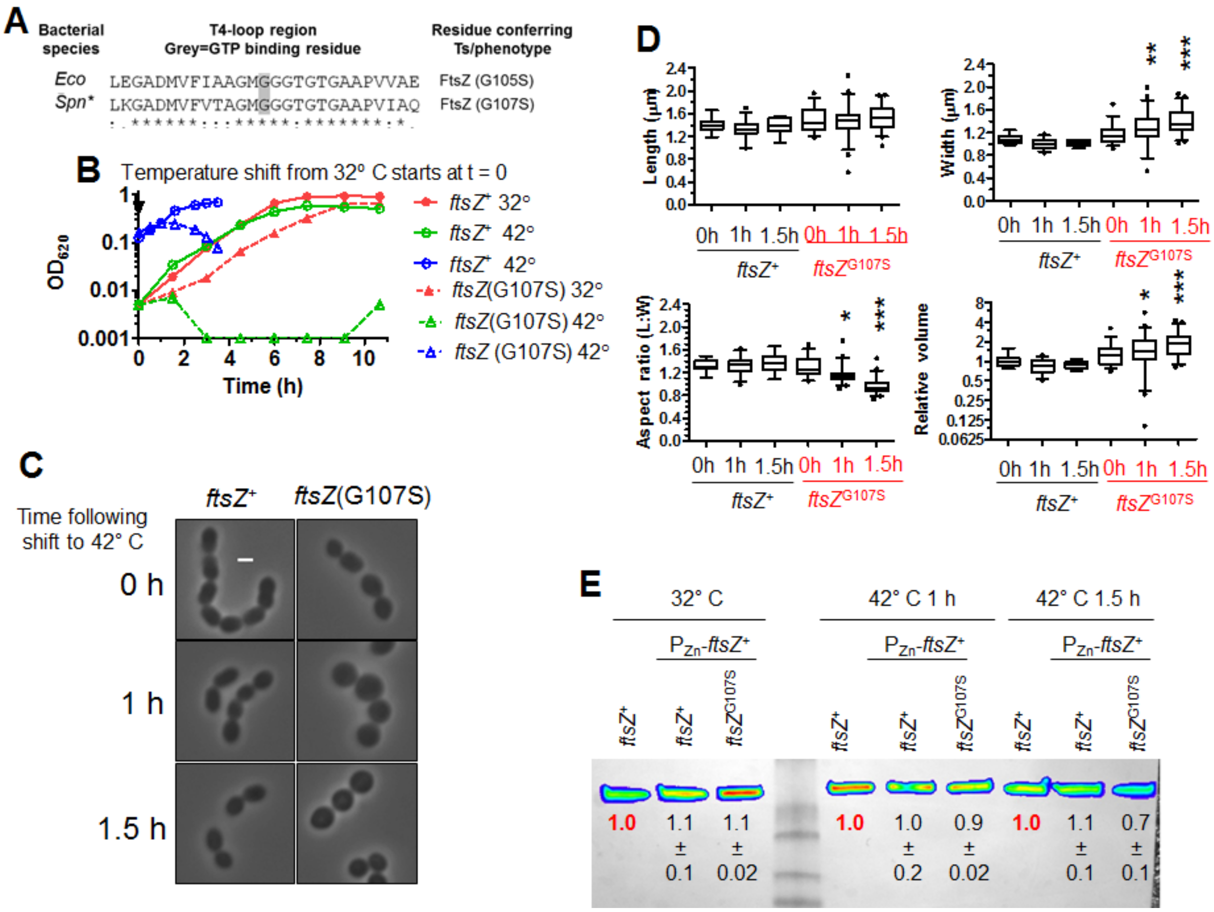

Fig. S13. Temperature sensitive phenotype of *Spn* mutant carrying *ftsZ*(G107S). Wild-type strain IU1945 (*ftsZ*$^+$), merodiploid strain IU8122 (*ftsZ*$^+$//P$_{Zn}$-*ftsZ*$^+$), and merodiploid strain IU10612 (*ftsZ*(G107S)//P$_{Zn}$-*ftsZ*$^+$) were grown in BHI broth at 32° C from frozen glycerol stocks until early exponential phase (OD_{620} = 0.1-0.4). Cultures were diluted into fresh BHI broth (no $ZnCl_2$/$MnSO_4$ added) at a starting OD_{620} ≈ 0.005 at 32° C or 42° C (OD_{620} ≈0.1 for microscopic visualization). Cultures were monitored and phase-contrast microscopy was performed as described in SI Appendix, Experimental Procedures. Data are from 2-3 independent biological replicates. Note that all experiments in this figure were performed in the absence of added $ZnCl_2$/$MnSO_4$. (A) Amino acid alignment of FtsZ from three different bacterial species indicates that *Spn* FtsZ(G107) is the conserved residue corresponding to the GTP

binding site established for *E. coli* K-12 FtsZ (P0A9A6) (1, 2). FtsZ(G105S) is within the T4 loop region originally identified in *Methanococcus jannaschii* FtsZ (3, 4). (B) Growth curve of IU1945 (*ftsZ*$^+$) and IU10612 (*ftsZ*(G107S)//P$_{Zn}$-*ftsZ*$^+$) in the absence of added $ZnCl_2$/$MnSO_4$ at the temperatures indicated. (C) Phase-contrast images of IU1945 and IU10612 showing representative fields of cells; scale bars = 1 µm. (D) Box-and-whisker plots (whiskers, 5 and 95 percentile) of lengths, widths, aspect ratio, and relative volumes of cells of WT strain IU1945 (*ftsZ*$^+$) compared to that of temperature sensitive strain IU10612 (*ftsZ*(G107S)//P$_{Zn}$-*ftsZ*$^+$) following a shift to 42° C. P values relative to WT parent strain IU1945 (*ftsZ*$^+$ at t = 0) were obtained by one-way ANOVA analysis (GraphPad Prism, nonparametric Kruskal-Wallis test). $P<0.01$, **; $P<0.001$, ***. (E) Western blot of 8.4 µg of lysates of strains IU1945 (*ftsZ*$^+$) (left), IU8122 (*ftsZ*$^+$//P$_{Zn}$-*ftsZ*$^+$) (middle), and IU10612 (*ftsZ*(G107S)//P$_{Zn}$-*ftsZ*$^+$) (right) grown at the temperatures indicated. Western blotting was performed as described in SI Appendix, Experimental Procedures using anti-FtsZ antibody. Relative average amounts of FtsZ protein (± SD) are indicated from two independent biological replicates.

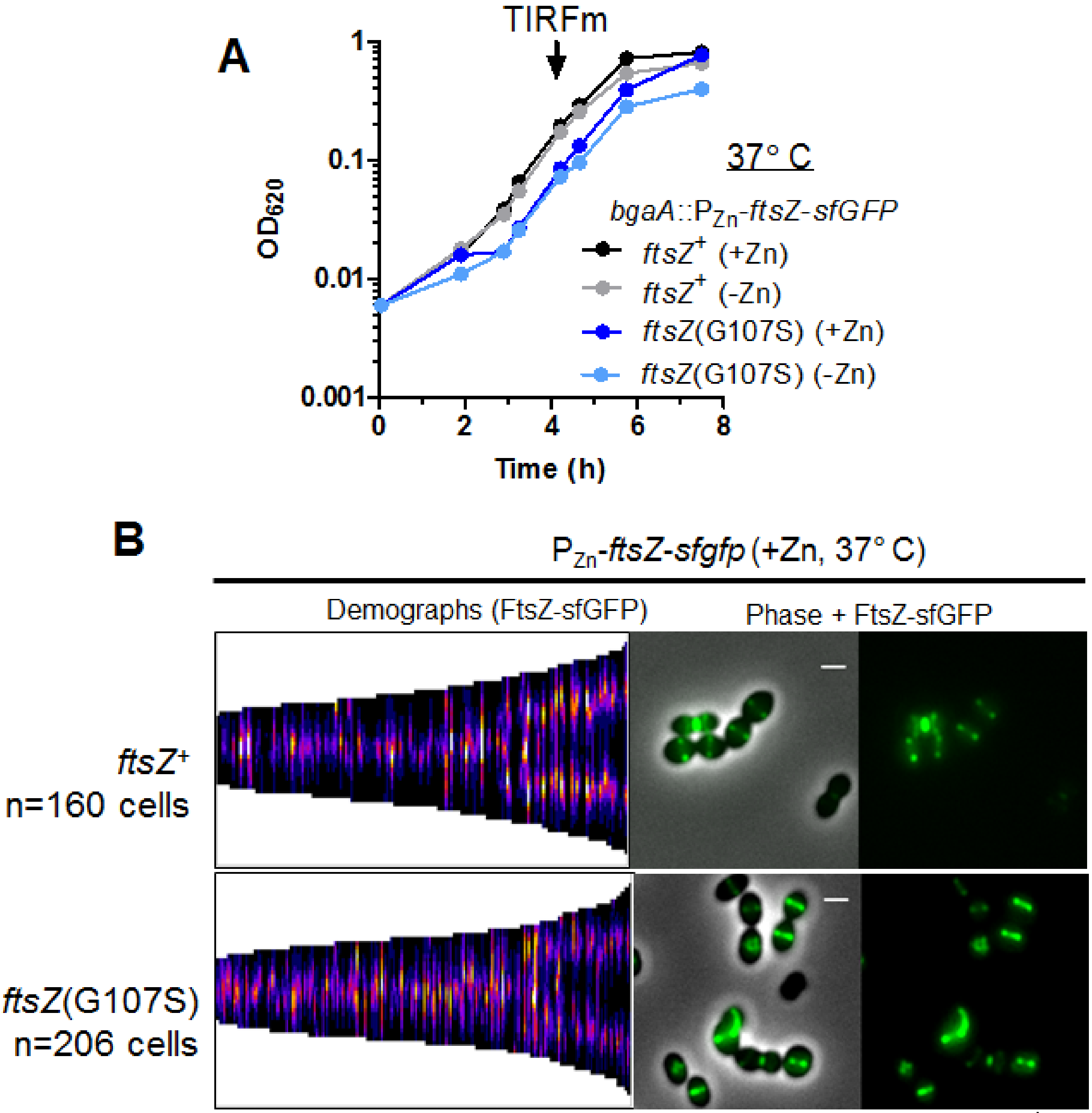

Fig. S14. Localization of FtsZ-sfGFP in strains expressing native *ftsZ*$^+$ or *ftsZ*(G107S). (A) Growth curves of merodiploid strains IU13315 (*ftsZ*$^+$//P_{Zn}-*ftsZ-sfgfp*) and IU14375 (*ftsZ*(G107S)//P_{Zn}-*ftsZ-sfgfp*) in C+Y, pH 6.9 with added 0.1 mM $ZnCl_2$ and 0.01 mM $MnSO_4$ (+Zn) or lacking Zn/Mn (-Zn) at 37° C in 5% CO_2. At this $ZnCl_2$/$MnSO_4$ concentration, FtsZ-sfGFP is expressed from an ectopic site in a relatively low amount that does not cause growth or cell morphology defects in strain IU13315 (see (B)). The

arrow indicates when samples were taken for TIRFm (see SI Appendix, Fig. S15). (B) Demographs generated using MicrobeJ (see SI Appendix, Experimental Procedures and (5)) and representative images of IU13315 (*ftsZ*$^{+}$//P_{Zn}-*ftsZ-sfgfp*) and IU14375 (*ftsZ*(G107S)//P_{Zn}-*ftsZ-sfgfp*) growing exponentially in C+Y, pH 6.9 with added Zn/Mn (+Zn). Data are from two independent biological replicates.

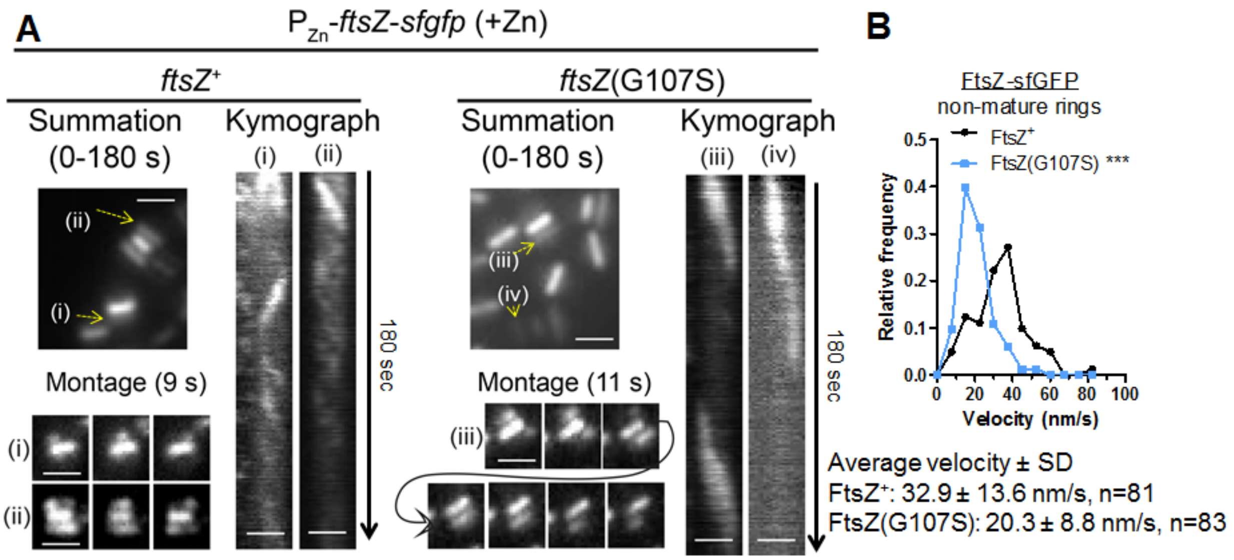

Fig. S15. Velocity of FtsZ filaments/bundles is decreased in a FtsZ(G107S) GTPase mutant. Cells of merodiploid strains expressing FtsZ$^+$ (IU13315) or FtsZ(G107S) (IU14375) from the native chromosomal locus and a limited amount of FtsZ-sfGFP from the ectopic *bgaA* site were grown and observed by TIRFm (see SI Appendix, Experimental Procedures and Movie S5). Cells were imaged on agarose pads containing C+Y, pH 7.1 at 37° C (normal atmosphere) with added 0.1 mM $ZnCl_2$ and 0.01 mM $MnSO_4$ (+Zn). (A) Summations of frames of 180 s movies; representative montages of images of cells at 9 s or 11 s intervals; and kymographs (180 s) of the FtsZ-sfGFP rings marked (i-iv) in the summations. (B) Distribution of velocities of FtsZ-sfGFP in filaments/bundles also containing FtsZ$^+$ or FtsZ(G107S) in nascent rings and early equatorial rings. Velocity values are from two independent biological replicate experiments, binned in increments of 7.5 s. P values were obtained by one-way unpaired, two-tailed t-tests (GraphPad Prism), where P<0.001, ***.

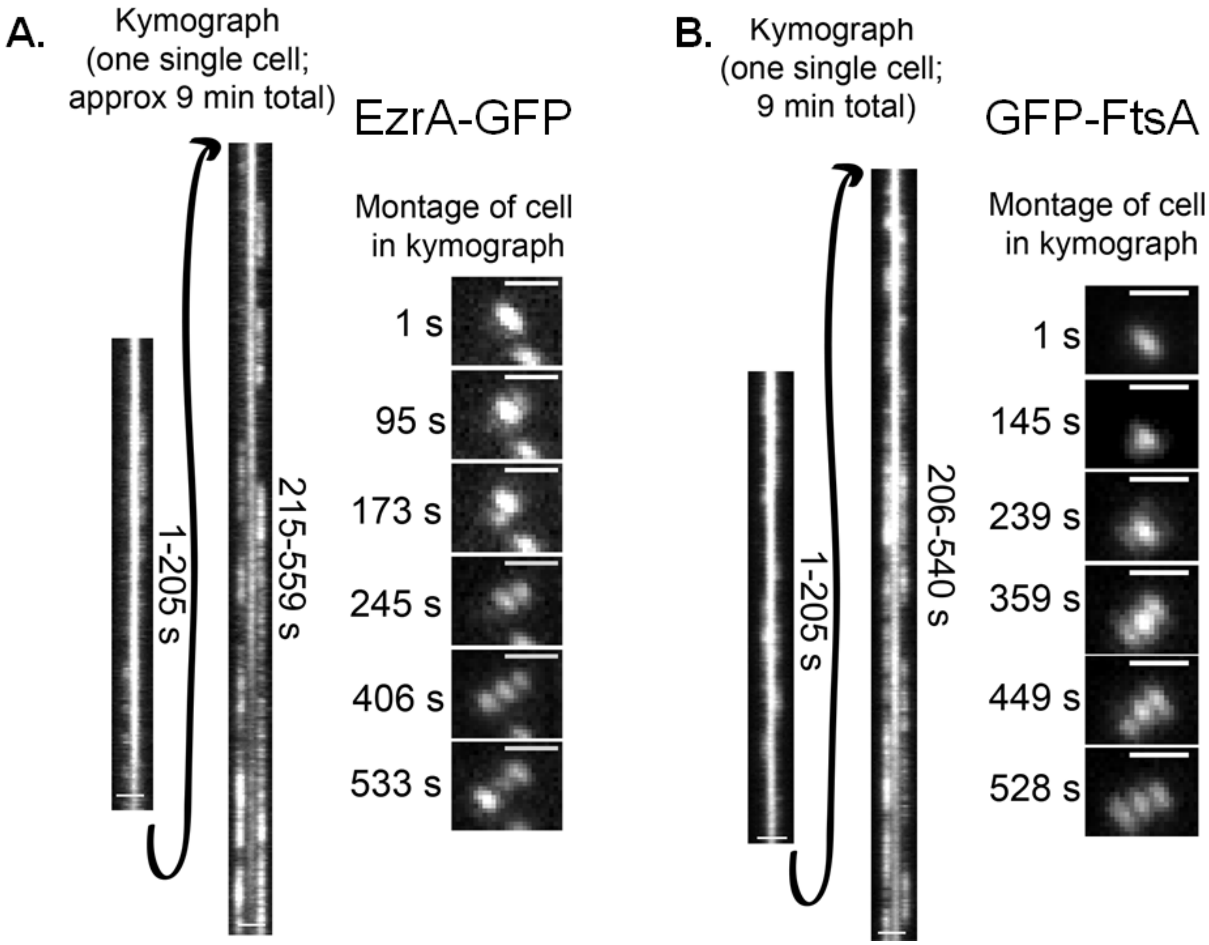

Fig. S16. Nascent ring planes containing EzrA and FtsA move outward from septa. TIRFm was performed on strains expressing EzrA-GFP (IU10449) or GFP-FtsA (IU10035) as described in SI Appendix, Experimental Procedures. Cells were imaged for the indicated times (approximately 9 min total at 1 frame/s). Kymographs were drawn across the long axis of cells (line width = 4). Data are representative from two independent biological replicates. Representative analyses are shown of the more than 10 cells analysed per strain. Scale bars = 1.0 µm.

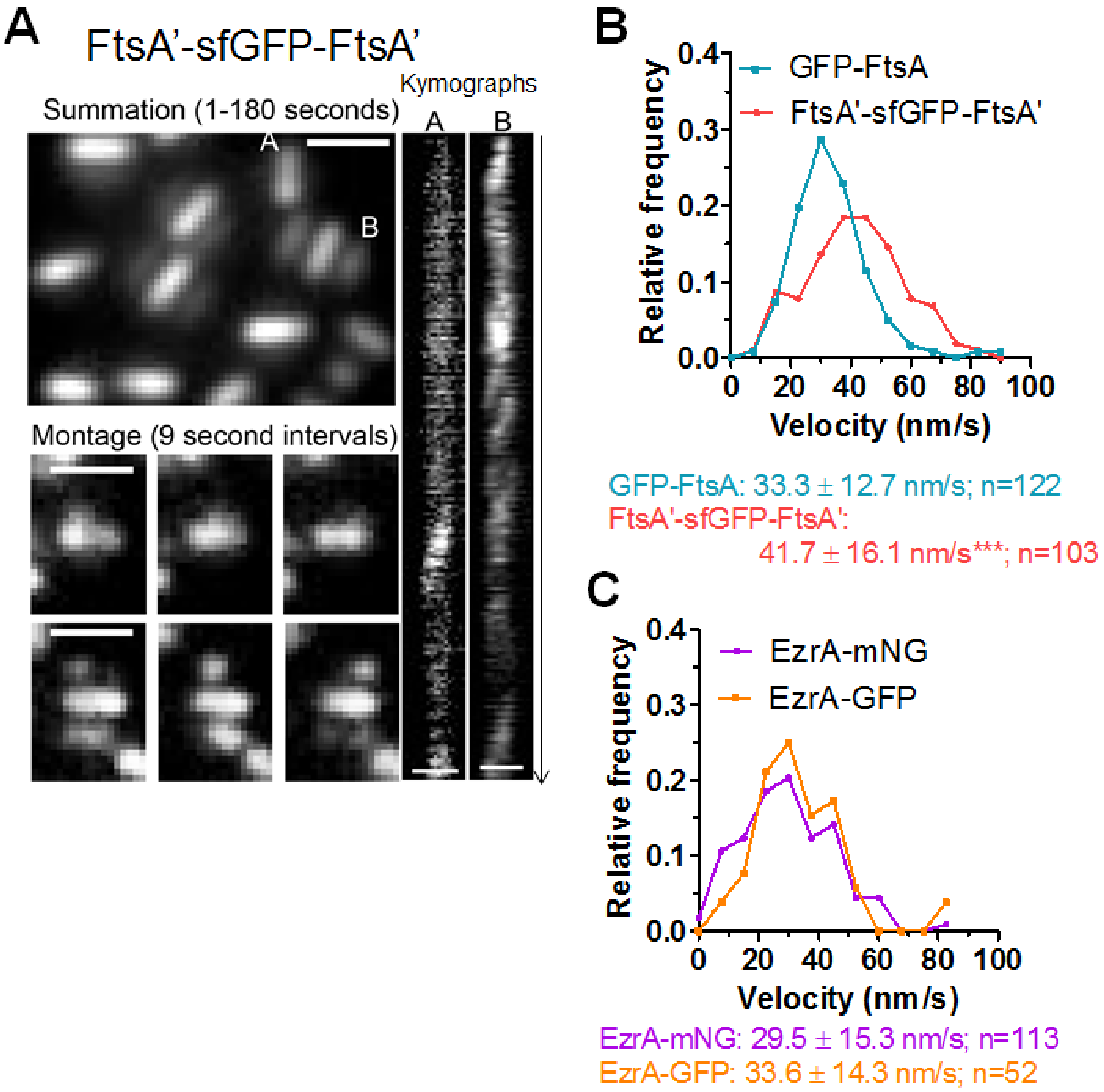

Fig. S17. Dynamics of different FtsA- and EzrA-tagged constructs. Conventional TIRFm was performed on cells containing fluorescent protein fusions expressed from native chromosomal loci as the sole source of EzrA or FtsA as described in SI Appendix, Experimental Procedures. Images were acquired at 1 frame/s. (A) Summation of a field of cells, montages of individual cells, and respective kymographs along the nascent ring plane (top montage; A) or equatorial ring plane (bottom montage;

B) of cells expressing FtsA'-sfGFP-FtsA' (IU13662). Scale bars = 1 µm. Data are representative of three independent biological replicates. (B) Histogram displaying the velocity distribution of GFP-FtsA (IU10035) versus FtsA'-sfGFP-FtsA' (IU13662). P values were obtained by one-way unpaired, two-tailed t-tests (GraphPad Prism), where P<0.001, ***. Values are from 2 or 3 independent biological replicates. (C) Histogram displaying the velocity distribution of EzrA-mNG (IU14117) versus EzrA-GFP (IU10449). No significant difference was indicated by one-way unpaired, two-tailed t-tests (GraphPad Prism). Values are from two or more independent biological replicates.

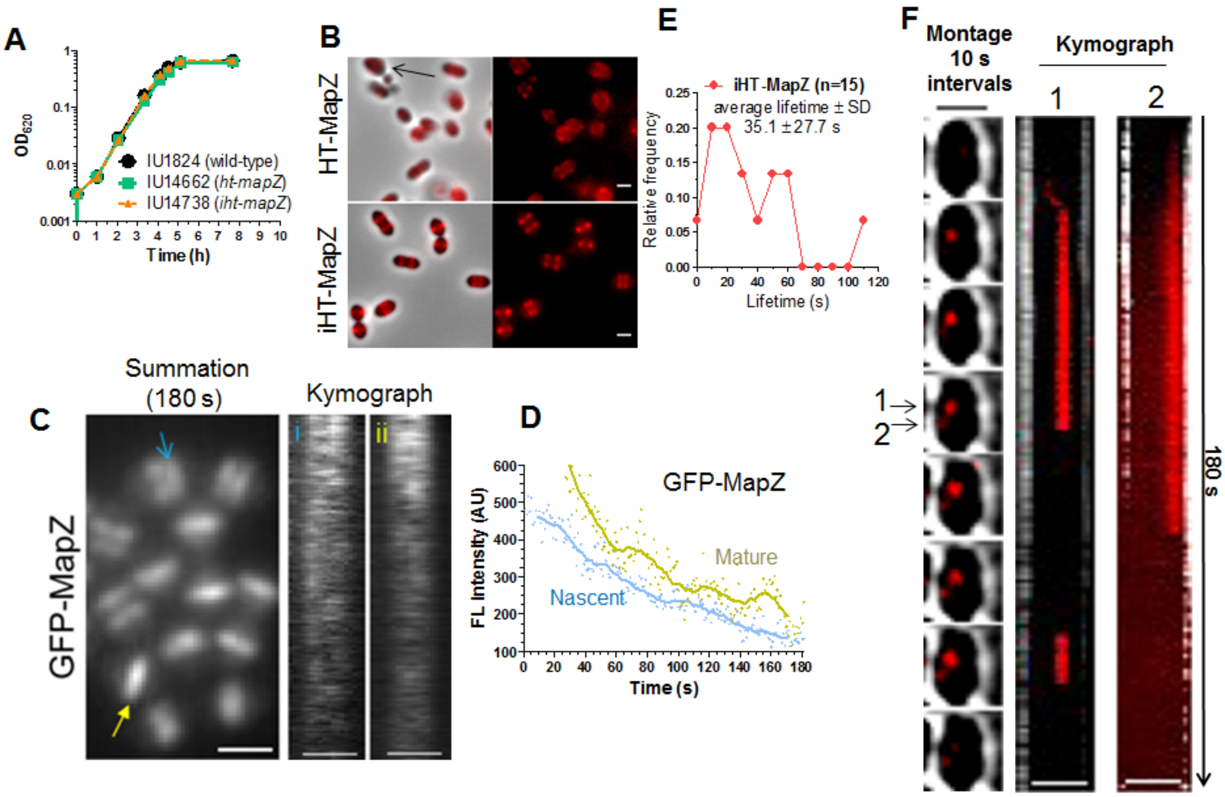

Fig S18. MapZ rings are stable in *Spn*. Strains expressing HT-MapZ (IU14662), iHT-MapZ (IU14738), or GFP-MapZ (IU9182) were cultured in C+Y pH 6.9 (5% CO_2) (A and B), and TIRFm was performed in C+Y, pH 7.1 (no CO_2) at 37° C (C-F) as described in SI Appendix, Experimental Procedures. Data are from two independent biological replicates. Scale bars = 1.0 µm. (A) Growth curves of strains relative to the wild-type parent. (B) Representative epifluorescence microscopy images (red) overlaid with phase-contrast images showing the localization of HT-MapZ or iHT-MapZ labeled with HT-TMR ligand to saturation as described in SI Appendix, Experimental Procedures. Cells expressing HT-MapZ (black arrow) show defects in cell shape and aberrant diffuse localization that were not observed for cells expressing iHT-MapZ, which were used in all subsequent experiments. (C) GFP-MapZ dynamics was determined by TIRFm (see Movie S8). Summation of frames from a 180 s movie of cells expressing

GFP-MapZ, accompanied by kymographs (180 s) corresponding to rings marked by colored arrows in the summation (i, nascent ring, blue; ii; mature ring, yellow). (D) Mean fluorescence intensity (dots) and corresponding moving average (every 20 points; solid lines) of GFP-MapZ in the ring planes from (C) as a function of time in 1 s intervals. (E) SM-TIRFm demonstrating lack of motion of iHT-MapZ molecules labeled with a limiting amount of HT-JF549 ligand (see SI Appendix, Experimental Procedures and Movie S9). The histogram shows the lifetimes of static iHT-MapZ signals in 13 cells, including those in (F), which shows a representative montage in 10 s intervals and kymographs of the nascent ring planes marked 1 or 2 by arrows in the montage.

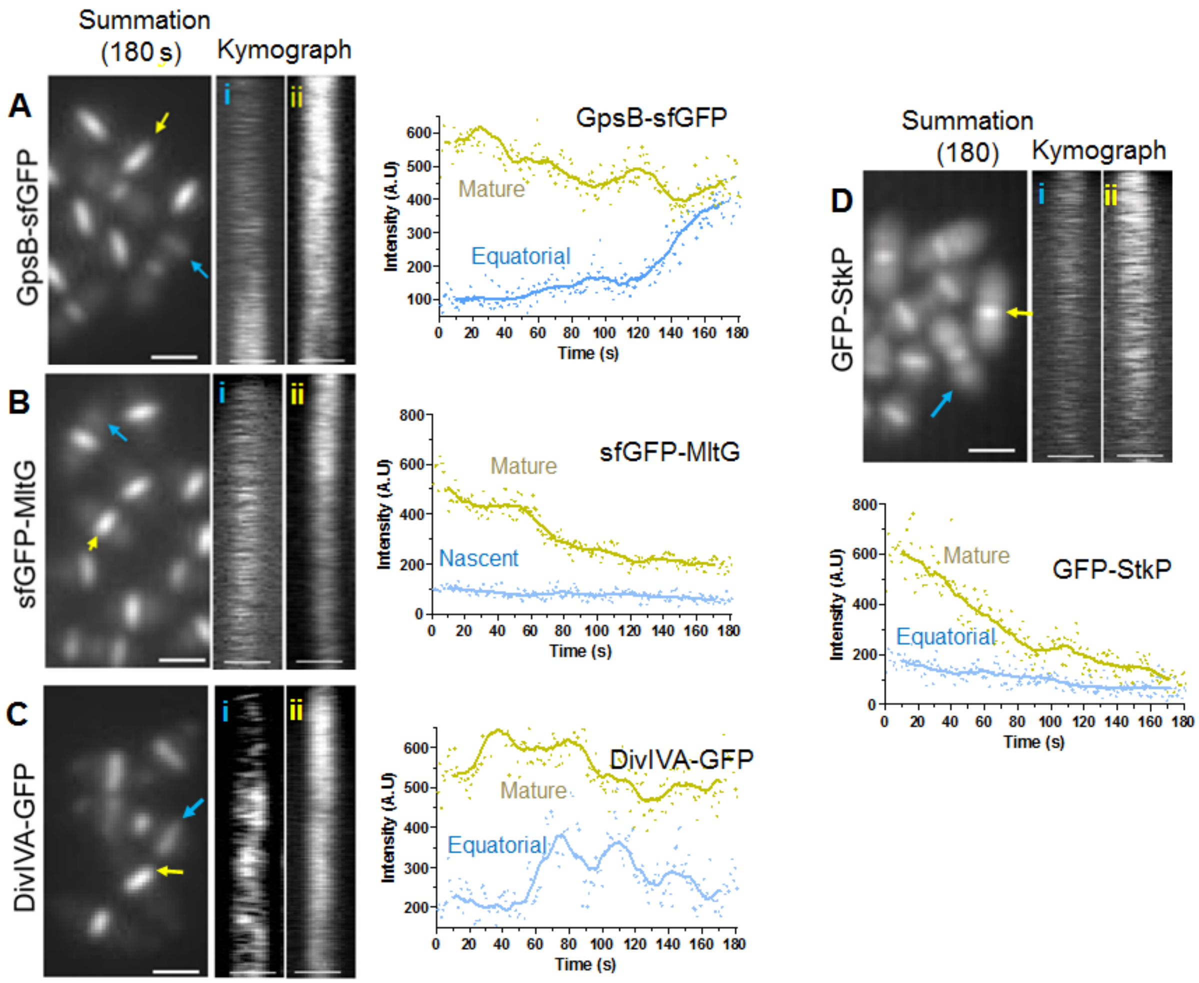

Fig S19. Different dynamic fluctuation patterns of *Spn* PG synthesis and cell division proteins observed by TIRFm. Motion of proteins that leave mature septa late in cell division (Fig. 1), including GpsB (strain IU11638; Movie S11), MltG (strain IU11005; Movie S12), DivIVA (strain IU9167; Movie S13), and StkP (strain IU9164; Movie S14), were examined by TIRFm as described in SI Appendix, Experimental Procedures. Summations of 180 s movies with accompanying kymographs and traces of mean fluorescence intensity (dots) and moving averages (every 20 points; solid lines) are shown for the indicated ring planes (i, blue, equatorial/nascent; ii; yellow, mature septal).

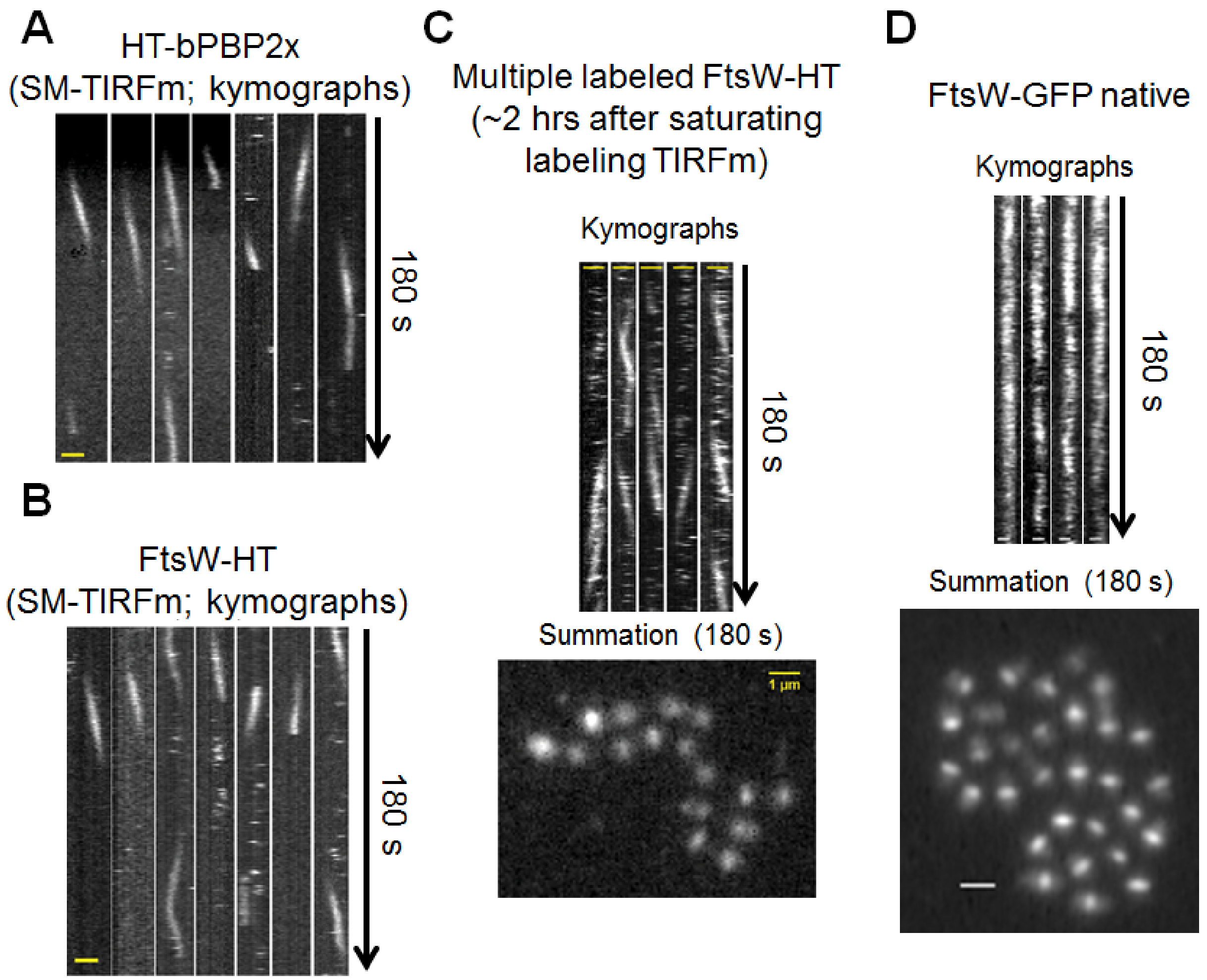

Fig S20. Dynamics of bPBP2x and FtsW movement determined by SM-TIRFm and TIRFm. SM-TIRFm and TIRFm were performed on strains expressing HT-bPBP2x (IU13910), FtsW-HT (IU15096), and FtsW-GFP (IU8918). Images were acquired at 1 frame/s. Lines were drawn along the septa or equatorial ring planes of cells to generate kymographs (180 s). Summations of fields of cells are shown, where indicated. Single-molecule or saturation labeling TIRFm was performed with HT-JF549 ligand as described in SI Appendix, Experimental Procedures*.* Scale bars = 1.0 µm, except where indicated below in the kymographs in (D). (A) Kymographs of labeled molecule paths from SM-TIRFm experiments performed on IU13910 (HT-bPBP2x). (B) Kymographs of labeled molecule paths from SM-TIRFm experiments performed on IU15096 (FtsW-HT).

(C) Kymographs of labeled molecule paths from saturation-labeling. TIRFm experiments performed on IU15096 (FtsW-HT). TIRFm was performed approximately 2 h after washing the unbound ligand away. (D) Kymographs of ring planes containing FtsW-GFP from TIRFm experiments performed on IU8918. Scale bars shown at the bottom of the kymographs = 0.5 µm. Movie S17 shows SM-TIRFm of cells expressing FtsW-HT.

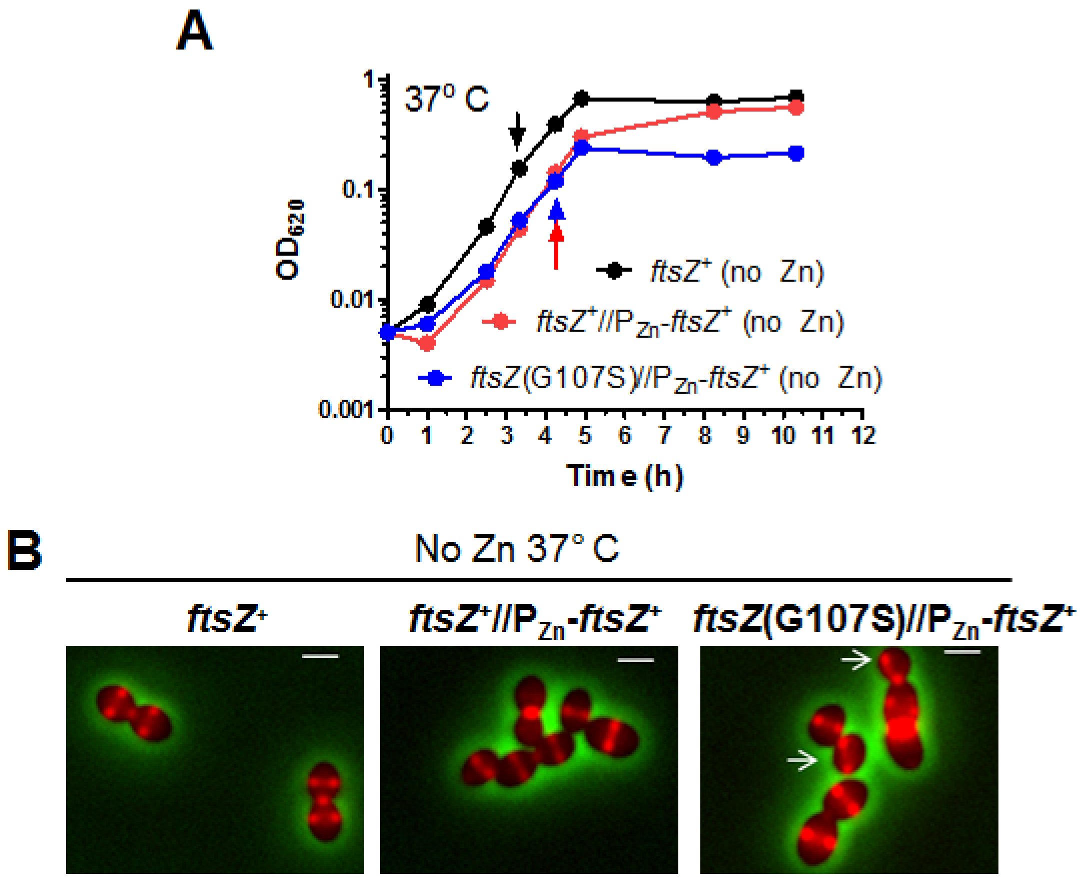

Fig. S21. FtsZ(G107S) causes aberrant placement of some planes of PG synthesis. Wild-type strain *ftsZ*$^+$ (IU1824) or merodiploid strains *ftsZ*$^+$//P_{Zn}-*ftsZ*$^+$ (IU12286), or *ftsZ*(G107S)//P_{Zn}-*ftsZ*$^+$ (IU14377) were grown in C+Y, pH 6.9 at 37° C (5% CO_2) and labeled with the FDAA, TADA, for 2.5 min, washed, and fixed for microscopy as described in SI Appendix, Experimental Procedures. Zn/Mn was not added (no Zn). (A) Growth monitored by OD_{620}. Color-coded arrows indicate the times at which corresponding cultures were sampled and labeled with the FDAA. (B) Representative overlaid phase-contrast and epifluorescence images showing FDAA labeling (red). White arrows point out aberrant planes of PG synthesis in the strain expressing FtsZ(G107S). Scale bar = 1µM. The experiment was performed twice with similar results.

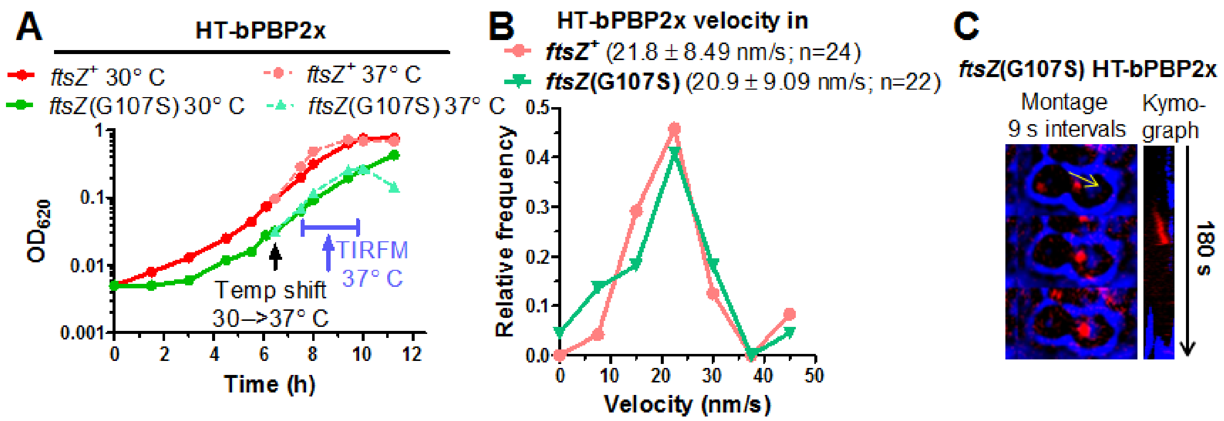

Fig. S22. FtsZ(G107S) does not affect the velocity of bPBP2x, but can affect the plane of bPBP2x movement. Directionally moving HT-bPBP2x single molecules were tracked by SM-TIRFm as described in SI Appendix, Experimental Procedures. Growth and velocities were determined in strains expressing *ftsZ*+ (IU13910) or *ftsZ*(G107S) (IU14508) from the native *ftsZ* locus. Strains were labeled for SM-TIRF with 120 pM HT-JF549 ligand. Data are from two independent biological replicates. (A) Growth was monitored by OD_{620} for strains growing in C+Y, pH 6.9 at 30° C (5% CO_2). The black arrow indicates when cultures were switched to 37° C, and the purple arrow and bracket indicate when cultures were sampled for SM-TIRFm. (B) Histogram of HT-bPBP2x velocities. Values are binned in 7.5 nm/s and average velocity ± SD is shown. No significant difference in HT-bPBP2x velocity was detected in the *ftsZ*+ and *ftsZ*(G107S) strains, as determined by one-way unpaired, two-tailed t-tests (GraphPad Prism). (C) Montage of movement of a HT-bPBP2x molecule in FtsZ(G107S) strain (IU14508) (see Movie S18). The yellow arrow indicates the path of movement along the long-axis of the cell. Kymograph analysis demonstrates the directional movement of HT-bPBP2x even on the long axis of the cell. The histogram of velocities in (B) contains HT-bPBP2x molecules moving on any plane in FtsZ(G107S) mutant cells.

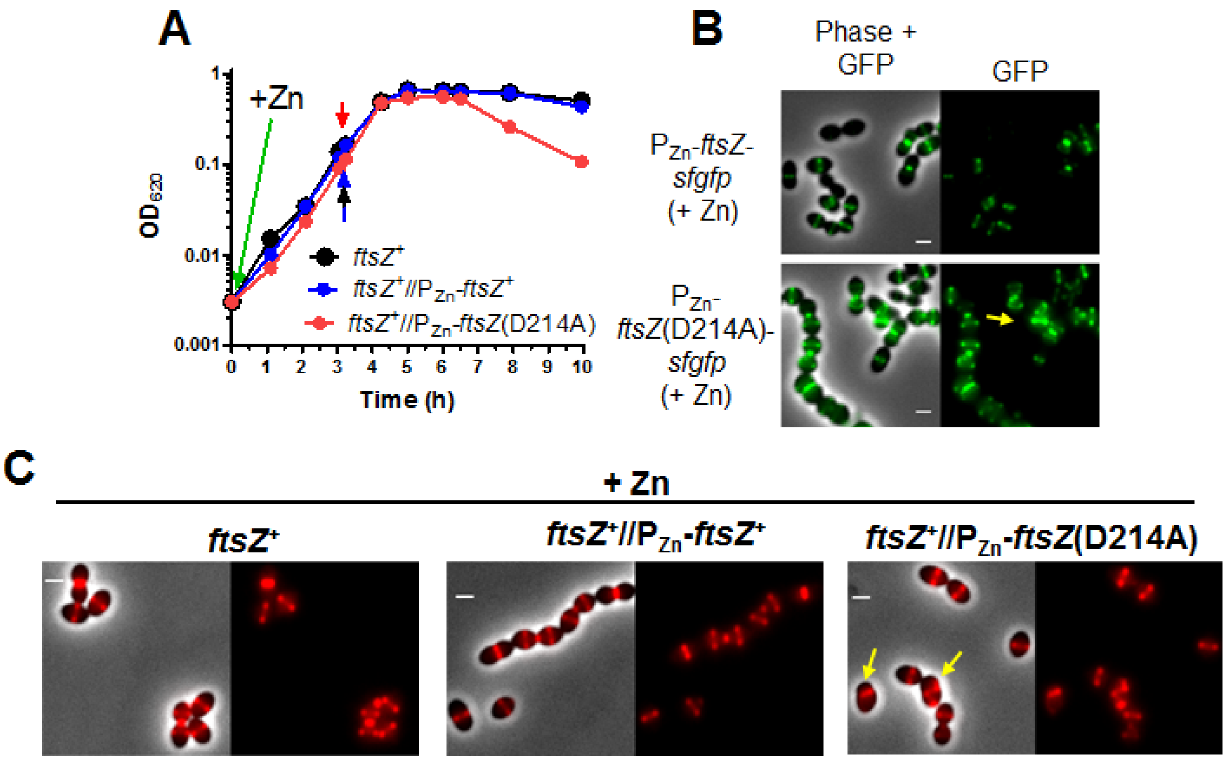

Fig. S23. Overexpression of FtsZ(D214A) causes lower growth yield, earlier autolysis, and aberrant planes of PG synthesis. (A) Wild-type strain *ftsZ*$^+$ (IU1824), or merodiploid strains *ftsZ*$^+$//P_{Zn}-*ftsZ*$^+$ (IU12286) and *ftsZ*$^+$//P_{Zn}-*ftsZ-sfgfp* (IU13315), or *ftsZ*$^+$//P_{Zn}-*ftsZ*(D214A) (IU14850) and *ftsZ*$^+$//P_{Zn}-*ftsZ*(D214A)-*sfgfp* (IU14852) were grown in C+Y pH 6.9 at 37° C (5% CO_2). Following dilution to start a new culture (green arrow; t = 0), 0.25 mM $ZnCl_2$ and 0.025 mM $MnCl_2$ (+Zn) was added to induce FtsZ(D214A) expression and growth was monitored at OD_{620}. At $OD_{620} \approx 0.1$, cells were sampled (colored arrows), concentrated by brief centrifugation, and observed by epifluorescence microscopy (B) or labeled with TADA for 2.5 min, washed, and fixed (C), as described in SI Appendix, Experimental Procedures. Representative phase-contrast images are overlaid with fluorescence images in (B) (sfGFP; green) and (C) (FDAA labeling, red). Yellow arrows in (C) point to aberrantly placed sites of PG synthesis in the strain overexpressing FtsZ(D214A). Scale bar = 1µm.

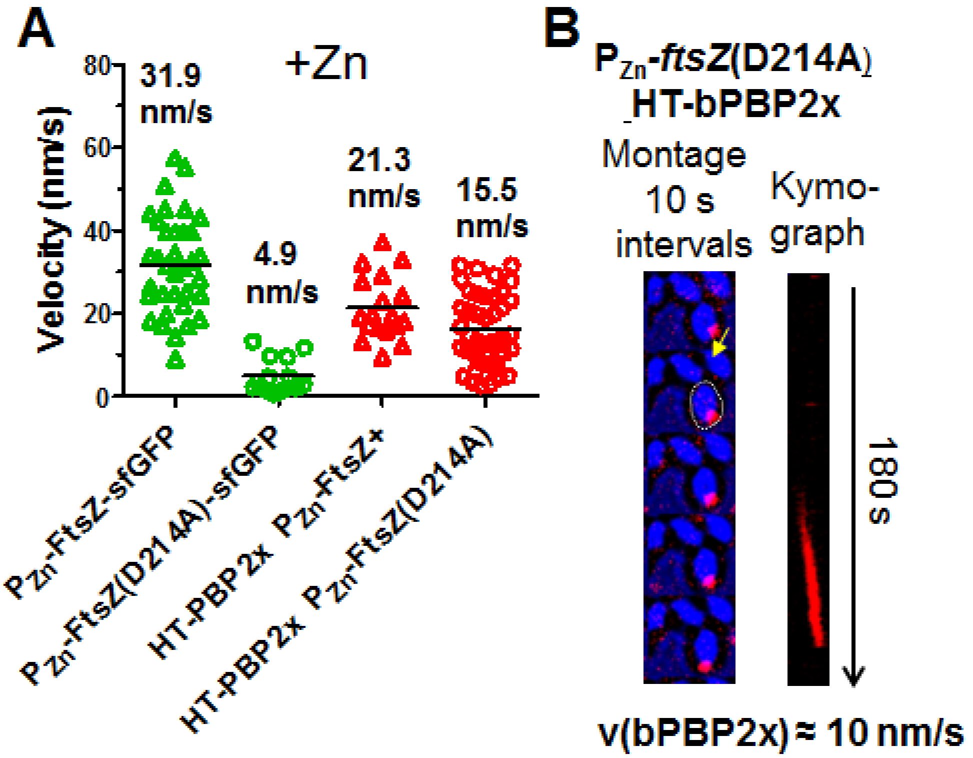

Fig. S24. Ectopic induction of FtsZ(D214A) greatly (≈7X) reduces the velocity of FtsZ filament treadmilling, while only slightly (≈1.3X) decreasing the velocity of bPBP2x single molecules. (A) Velocities of FtsZ variants were determined by TIRFm in strains overexpressing *ftsZ-sfgfp* (IU13315; n = 35) or *ftsZ*(D214A)-*sfgfp* (IU14852 n = 14) in C+Y, pH 7.1 as described in SI Appendix, Experimental Procedures. FtsZ-sfGFP or FtsZ(D214A)-sfGFP was induced for 3-4 h by addition of 0.25 mM $ZnCl_2$ and 0.025 mM $MnSO_4$ (+Zn) prior to placing cells on agarose pads that also contained +Zn. Directionally moving single molecules of HT-bPBP2x were tracked by SM-TIRFm (see Movie S20), and velocities were determined in strains overexpressing *ftsZ*$^+$ (IU15038; n = 18) or *ftsZ*(D214A) (IU15041; n = 43). Strains were labeled with 120 pM HT-JF549 ligand to visualize single molecules of bPBP2x as described in SI Appendix, Experimental Procedures. Data are from two independent biological replicates. (A) Scatter plot where each dot represents a separate velocity

measurement. Average velocities are shown for each strain and median velocities are marked as black bars. (B) Example montage from movie of a HT-bPBP2x molecule in cells induced for FtsZ(D214A) expression. Approximate outline of cells (sketched lines) were determined by brightfield microscopy, where cell bodies are colored blue, and HT-bPBP2x appears as a red dot. The yellow arrow indicates an aberrant plane of HT-bPBP2x movement in a cell. The kymograph of directional movement of a single bPBP2x molecule is from a 180 s movie, giving a velocity ≈10 nm/s for this example. Note that FtsZ(D214A)-sfGFP was overexpressed in these experiments, whereas FtsZ(D214A) was overexpressed in strains expressing FtsZ-sfGFP in related experiments shown in Figure 8A, which gave similar results to those above.

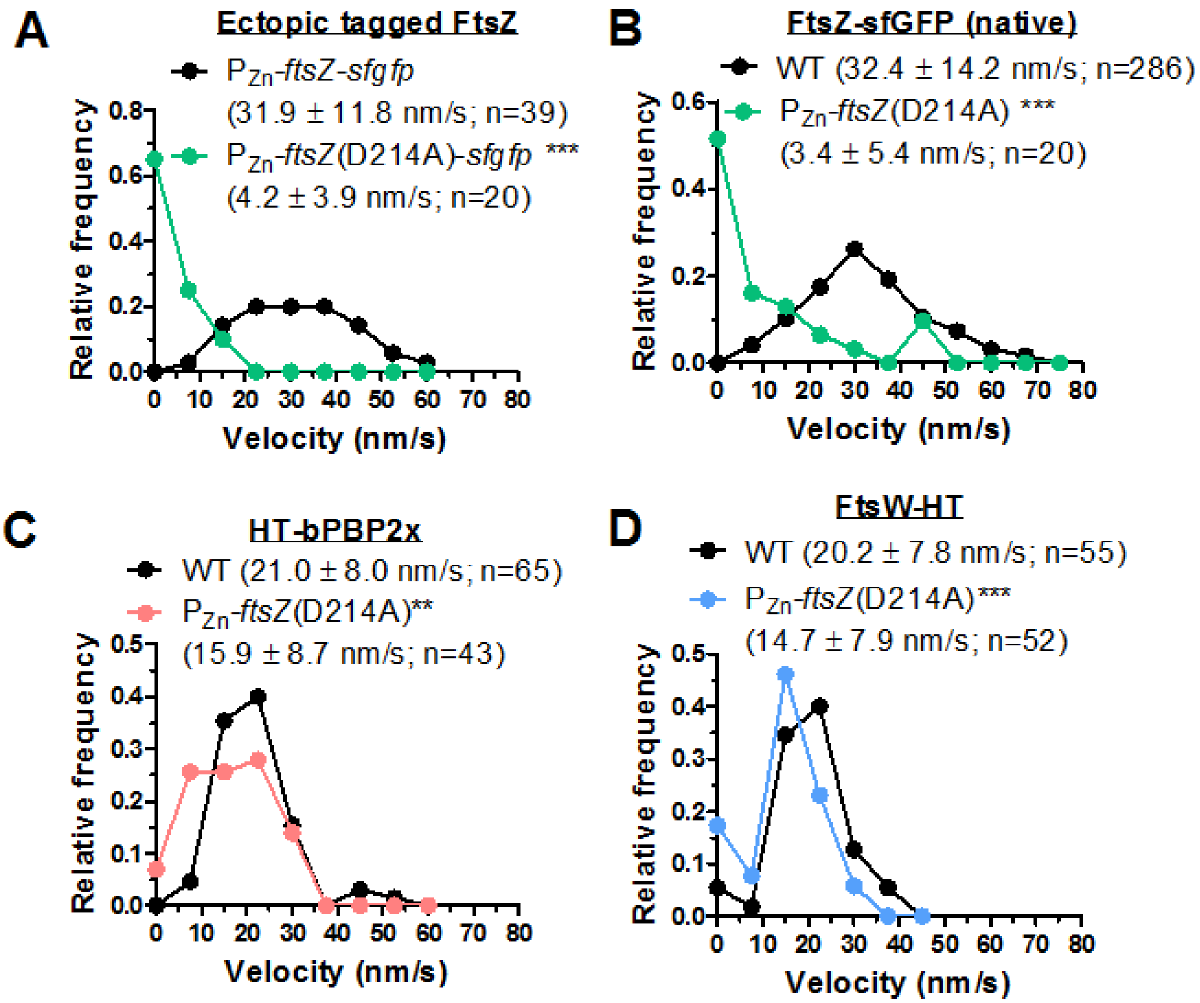

Fig. S25. Velocity of FtsZ-sfGFP treadmilling, but not HT-bPBP2x or FtsW-HT, is greatly decreased by overproduction of GTPase mutant FtsZ(D214A). TIRFm and SM-TIRFm were performed on bacteria on agarose pads containing C+Y, pH 7.1 (see SI Appendix, Experimental Procedures). 0.25 mM $ZnCl_2$ and 0.025 mM $MnSO_4$ (+Zn) was added to strains to expressing P_{Zn}-*ftsZ-sfgfp*, P_{Zn}-*ftsZ(D214A)-sfgfp*, or P_{Zn}-*ftsZ*(D214A). Control experiments showed that +Zn did not affect velocities of FtsZ-sfGFP or HT-bPBP2x in the WT strain. Histogram are shown of velocities of proteins (boldface, underlined) in the WT background (black curves) or when FtsZ(D214A) was overproduced (colored curves), binned in 7.5 nm/s intervals. The strains used are: (A)

IU13315 (black); IU14852 (green); (B) IU9985 (black); IU15181 (green); (C) IU13910 (black); IU15041 (pink); and (D) IU15096 (black); IU15175 (blue). Average velocities ± SD's are listed, where P values were obtained by one-way unpaired, two-tailed t-tests (GraphPad Prism; $P<0.001$; ***, $P<0.01$; **). Data are from 3 independent biological replicates.

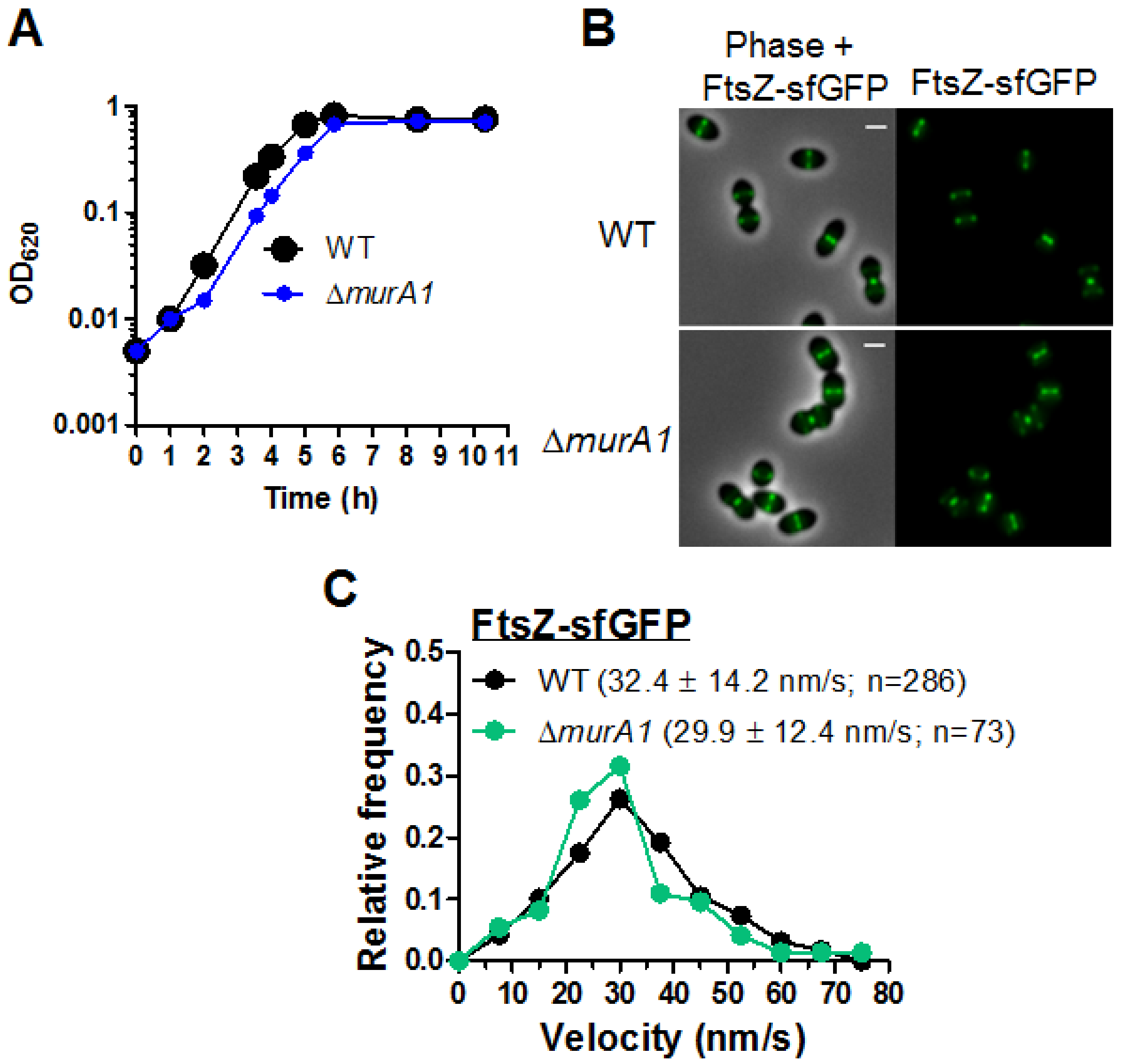

Fig. S26. Δ*murA1* has slight effects on pneumococcal growth and morphology but does not affect FtsZ localization or treadmilling velocity. (A) Growth curves of wild-type (IU1824) or Δ*murA1* mutant (IU13536) strains in C+Y, pH 6.9 (5% CO_2) cultures. Quantitation of cell lengths and widths by phase-contrast microscopy show that Δ*murA1* cells are slightly larger and rounder than WT cells, characteristic of a change in PG synthesis. (B) Overlaid phase-contrast and epifluorescence images of the WT strain (IU9985) and Δ*murA1* mutant (IU15141) expressing FtsZ-sfGFP from its

chromosomal locus shows normal FtsZ placement in the *murA1* mutant. Scale bar = 1 µm. (C) Histogram displaying the velocity distribution of FtsZ-sfGFP filaments/bundles in WT (IU9985) or Δ*murA1* mutant (IU15141) cells was obtained by TIRFm (see SI Appendix, Experimental Procedures). Average velocities ± SD's are listed. No significant difference in FtsZ-sfGFP velocities was detected between the WT and Δ*murA1* strains by one-way unpaired, two-tailed t-tests (GraphPad Prism). Data are from two independent biological replicates.

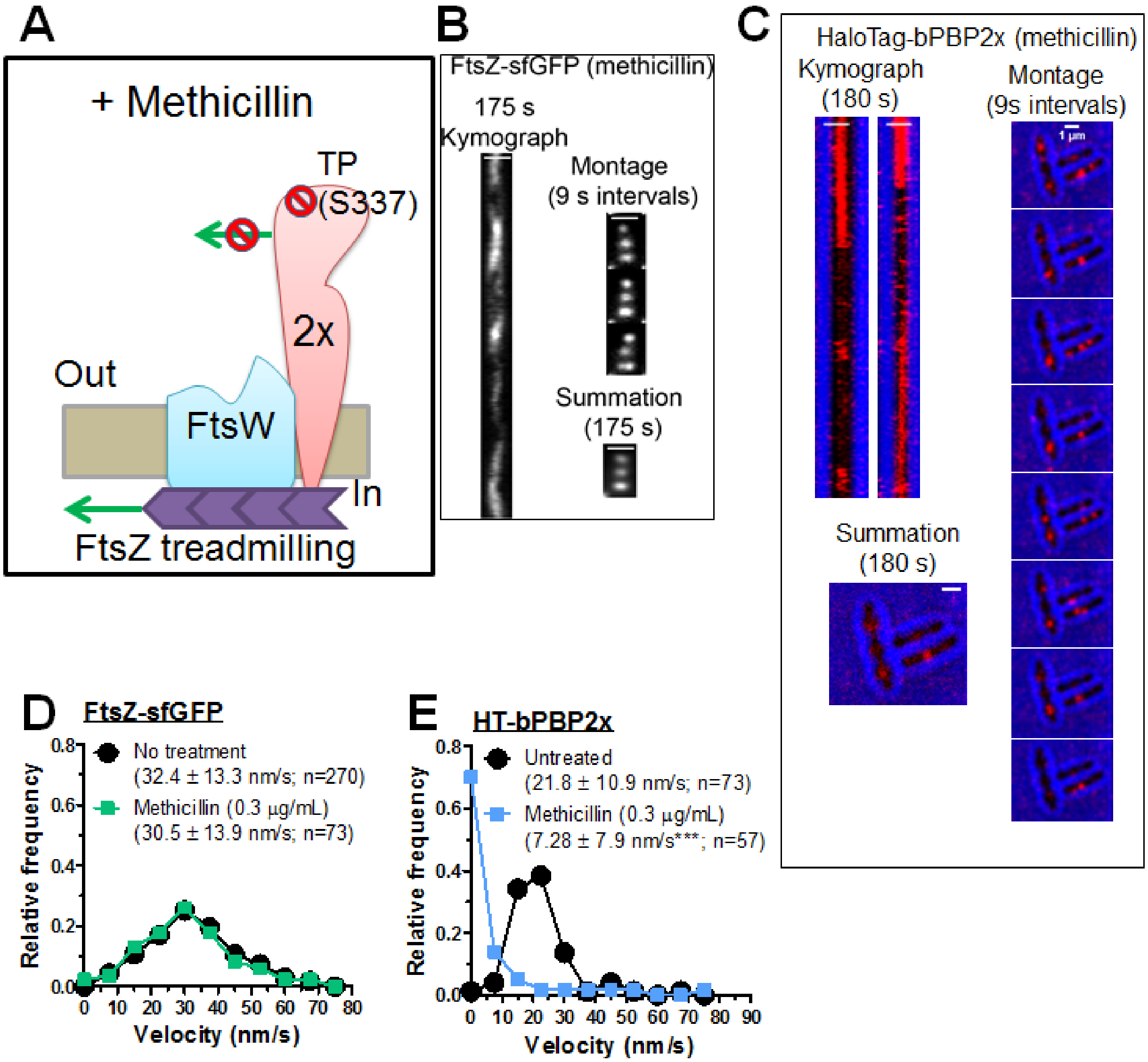

Fig. S27. Methicillin inhibition of bPBP2x TP activity stops bPBP2x circumferential movement, but not FtsZ treadmilling. Strains expressing FtsZ-sfGFP (IU9985) or HT-bPBP2x (IU13910) were exposed to 0.3 µg/mL methicillin on C+Y, pH 7.1 agarose pads for 45-75 min at 37° C, during which TIRFm (FtsZ-sfGFP) or SM-TIRFm (HT-bPBP2x) was performed as described in SI Appendix, Experimental Procedures. Prior to treatment with methicillin, strain IU13910 (HT-bPBP2x) was labeled with HT-JF549 ligand. (A) Cartoon summarizing the inhibitory effects of methicillin on bPBP2x circumferential movement, but not FtsZ treadmilling. (B) FtsZ-sfGFP

treadmilling still occurs when cells are treated with methicillin as shown by kymograph, montage, and summation (see Movie S21). (C) HT-bPBP2x does not move circumferentially as shown by kymograph, montage, and summation (see Movie S22). (D) and (E) Histograms displaying velocities of proteins (boldface, underlined) in the untreated (black curve) or methicillin-treated (colored curves), binned in 7.5 nm/s intervals. Average velocities ± SD are listed, where P values were obtained by one-way unpaired, two-tailed t-tests (GraphPad Prism), where $P<0.001$; ***. No significant difference was detected for FtsZ-sfGFP velocity in untreated and methicillin-treated cells. Data are from two independent biological replicates. Scale bars = 1.0 µm.

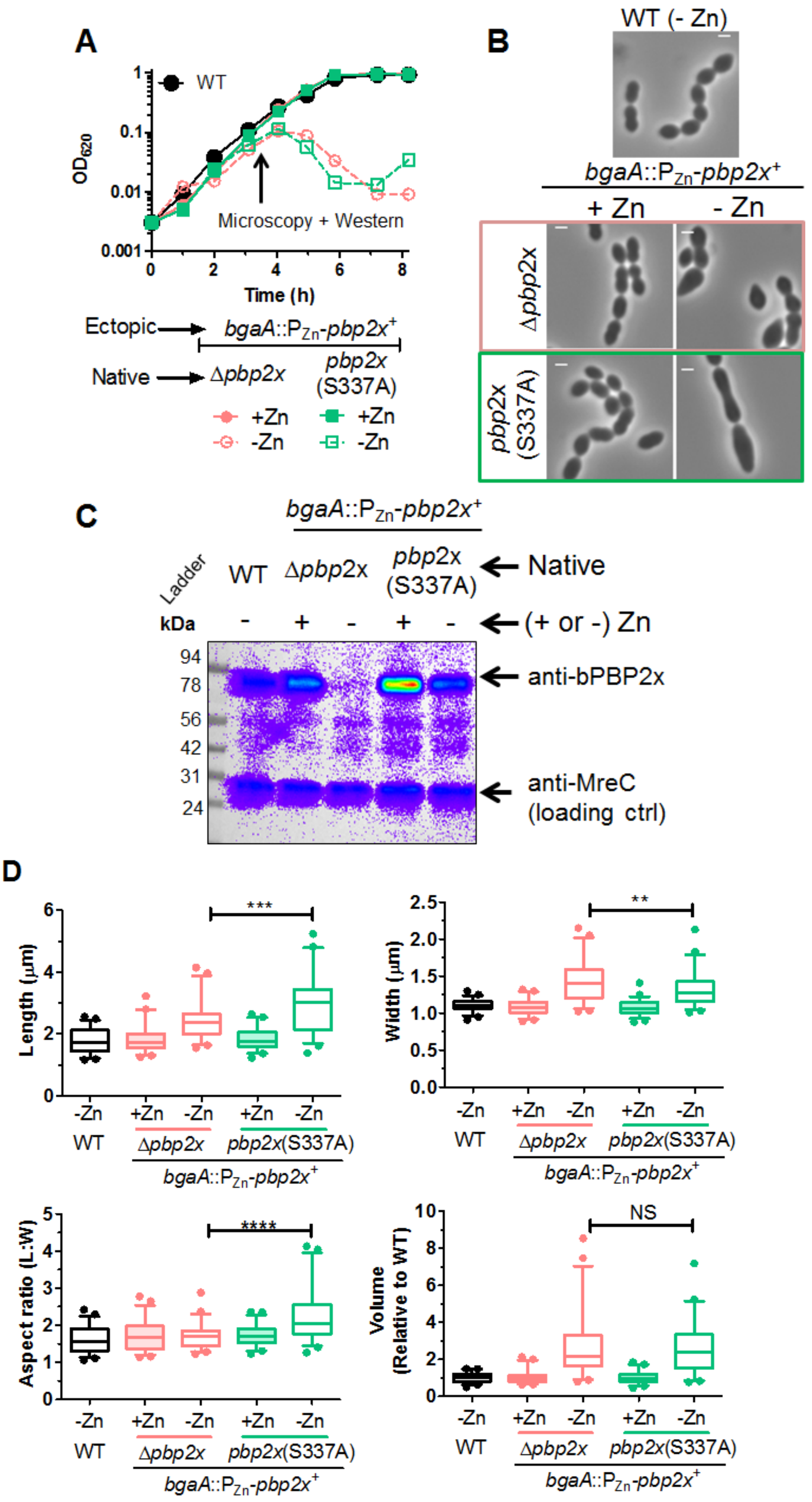

Fig. S28. Mutation change of an amino acid in the active site of bPBP2x-

transpeptidase is lethal in *Spn* cells, resulting in elongated cells that are morphologically different from bPBP2x depletion. Wild-type strain IU1824 (WT), bPBP2x depletion strain IU14059 (Δ*pbp2x*//P_{Zn}-*pbp2x*$^{+}$), and a merodiploid strain containing a mutation in the active site of bPBP2x expressed from the native locus, IU15246 (*pbp2x*(S337A)//P_{Zn}-*pbp2x*$^{+}$), were grown in BHI broth at 37° C from frozen glycerol stocks until early exponential phase (OD_{620} = 0.1-0.4) for less than 12 h. IU14059 and IU15246 were grown with added 0.4 mM $ZnCl_2$ and 0.04 mM $MnSO_4$ (+Zn) to BHI broth to allow expression of bPBP2x^{+}, since the Δ*pbp2x* and *pbp2x*(S337A) mutations are lethal. 2 mL cultures were centrifuged (16,500 × *g* for 5 min at 24° C), supernatant removed, and resuspended to OD_{620} ≈ 0.003 in 5 mL BHI with added 0.4 mM $ZnCl_2$ and 0.04 mM $MnCl_2$ (+Zn) or no added $ZnCl_2$/$MnSO_4$ (-Zn), and growth was monitored by OD_{620} (A). Samples were obtained for phase contrast microscopy (B and D) or western blot (C) at indicated time point in (A). The experiment was performed twice with similar results. (B) Representative images of phase contrast microscopy. Scale bars = 1 µm. (C) Western blot showing expression of mutant bPBP2x(S337A) protein. Western blotting was performed as described in SI Appendix, Experimental Procedures. Loading of lysates for western blotting was normalized to protein amount (4 µg of total protein of cell lysate per lane). The membrane was probed with anti-bPBP2x and anti-MreC as a loading control (6). (D) Quantification of cell shapes (length, width, aspect ratio) and sizes (volume, relative to WT) of indicated strains and conditions. Cells from pre-divisional (stage 1), early-divisional (stage 2), and mid-to-late divisional (stage 3) were included in this analysis. Length was the long cell axis, and the greatest width perpendicular to the long axis was measured at the equators of early-divisional

daughter cells and mid-to-late divisional cells, but not at constricting septa. A total of 50 cells was analyzed; 25 cells from each of two independent biological replicates. P values were obtained by one-way ANOVA analysis (with a Bonferroni post test; GraphPad Prism). Consistent with the micrographs in (B), the shape parameters of the WT (-Zn) and the complemented Δ*pbp2x* and *pbp2x*(S337A) mutants (+Zn) were statistically indistinguishable. In contrast, *pbp2x*(S337A) mutant cells depleted for bPBP2x^{+} were longer and slightly thinner than Δ*pbp2x* mutants depleted of bPBP2x^{+} ($P<0.01$).

References for SI Appendix, Figures and Legends

1. Bi E & Lutkenhaus J (1990) Analysis of *ftsZ* mutations that confer resistance to the cell division inhibitor SulA (SfiA). *J Bacteriol* 172(10):5602-5609.
2. Redick SD, Stricker J, Briscoe G, & Erickson HP (2005) Mutants of FtsZ targeting the protofilament interface: effects on cell division and GTPase activity. *J Bacteriol* 187(8):2727-2736.
3. Lowe J & Amos LA (1998) Crystal structure of the bacterial cell-division protein FtsZ. *Nature* 391(6663):203-206.
4. Nogales E, Downing KH, Amos LA, & Lowe J (1998) Tubulin and FtsZ form a distinct family of GTPases. *Nat Struct Biol* 5(6):451-458.
5. Ducret A, Quardokus EM, & Brun YV (2016) MicrobeJ, a tool for high throughput bacterial cell detection and quantitative analysis. *Nat Microbiol* 1(7):16077.
6. Zheng, J., Perez, A. J., Tsui, H. C. T., Massidda, O., & Winkler, M. E. (2017). Absence of the KhpA and KhpB (JAG/EloR) RNA-binding proteins suppresses the requirement for PBP2b by overproduction of FtsA in *Streptococcus pneumoniae* D39. *Mol Microbiol 106*(5): 793-814.
7. van Raaphorst R, Kjos M, & Veening JW (2017) Chromosome segregation drives division site selection in *Streptococcus pneumoniae*. *Proc Natl Acad Sci USA* 114(29):E5959-E5968.
8. Tsui HC, *et al* (2014). Pbp2x localizes separately from Pbp2b and other peptidoglycan synthesis proteins during later stages of cell division of *Streptococcus pneumoniae* D39. *Mol Microbiol* 94(1), 21-40.

SI APPENDIX, MOVIE LEGENDS

Movie S1. FtsZ-sfGFP dynamics in mature septal, nascent, and equatorial rings of dividing *Spn* cells. TIRFm was performed on strain IU9985 (*ftsZ-sfgfp*) as described in SI Appendix, Experimental Procedures. Images were taken at 1 s intervals over the course of 539 s (≈9 min). Movie is shown at 15 frames per s. See analysis in Fig. 1D.

Movie S2. Circumferential movement FtsZ-sfGFP (green) filaments/bundles in nascent rings on either side of mature septal FtsZ-rings in early divisional *Spn* cells (bright-field outline; blue border). TIRFm was performed on IU9985 (*ftsZ-sfgfp*) as described in SI Appendix, Experimental Procedures). Images were taken at 1 s intervals over the course of 380 s. Movie is shown at 15 frames per s. See analysis in Fig. 1B and 1C.

Movie S3. FtsZ-sfGFP dynamics in vertically immobilized *Spn* cells. Wide-field microscopy was performed on vertically immobilized cells of strain IU9985 (*ftsZ-sfgfp*) in microhole patterned agarose pads as described in SI Appendix, Experimental Procedures. Images were taken at 2.59 s intervals over the course of 3 min. Movie is shown at 3.86 frame per s (10x real speed). Scale bars = 500 nm. See analysis in Fig. 3.

Movie S4. Single molecules of FtsZ-HT (red) do not move in *Spn* cells (bright-field outlines; blue border), expressing EzrA-mNeonGreen (green) as a fiducial marker. SM-TIRFm was performed on strain IU14352 (*ftsZ-ht ezrA-mng*) pre-labeled with a limiting amount of HT-JF549 ligand as described in SI Appendix, Experimental Procedures. Images were taken at 1 s intervals over the course of 180 s. Movie is shown at 15 frames per s. See analysis in Fig. 4.

Movie S5. Directional movement of FtsZ-sfGFP is slowed by expression of FtsZ(G107S) expressed at the normal *ftsZ* chromosomal locus. TIRFm was performed on strain IU14375 (*ftsZ*(G107S)//P_{Zn}-*ftsZ-sfgfp*) induced with 0.1 mM $ZnCl_2$ and 0.01 mM $MnSO_4$ (+Zn) to induce a low level of FtsZ-sfGFP as described in SI Appendix, Experimental Procedures. Images were taken at 1 s intervals over the course of 180 s. Movie is shown at 15 frames per s. See analysis in SI Appendix, Fig. S15.

Movie S6. EzrA-mNeonGreen dynamics in mature septal, nascent, and equatorial rings of dividing *Spn* cells. TIRFm was performed on strain IU14117 (*ezrA-mng*) as described in SI Appendix, Experimental Procedures. Scale bar = 1 µm. Images were taken at 1 s intervals over the course of 180 s. Movie is shown at 15 frames per s. See analysis in Fig. 5.

Movie S7. GFP-FtsA dynamics in mature septal, nascent, and equatorial rings of dividing *Spn* cells. TIRFm was performed on strain IU10035 (*gfp-ftsA*) as described in SI Appendix, Experimental Procedures. Scale bar = 1 µm. Images were taken at 1 s intervals over the course of 180 s. Movie is shown at 15 frames per s. See analysis in Fig. 5B and 5C.

Movie S8. Minimal GFP-MapZ movement in mature septal and MapZ rings of *Spn.* TIRFm was performed on strain IU9182 (*gfp-mapZ*) as described in SI Appendix, Experimental Procedures. Scale bar = 1 µm. Images were taken at 1 s intervals over the course of 180 s. Movie is shown at 15 frames per s. See analysis in SI Appendix, Fig. S18C and S18D.

Movie S9. Single molecules of iHT-MapZ (red) lack mobility in septal and MapZ rings of *Spn* cells (bright-field outlines; blue border). SM-TIRFm was performed on

strain IU14738 pre-labeled with a limiting amount of HT-JF549 ligand as described in SI Appendix, Experimental Procedures. Images were taken at 1 s intervals over the course of 180 s. Movie is shown at 15 frames per s. See analysis in SI Appendix, Fig. S18E and S18F.

Movie S10. Aberrant streaming of EzrA-GFP in the absence of MapZ. TIRFm was performed on strain IU10540 (*ezrA-gfp* Δ*mapZ*) as described in SI Appendix, Experimental Procedures. Scale bar = 1 µm. Images were taken at 1 s intervals over the course of 180 s. Movie is shown at 15 frames per s. See analysis in Fig. 6B and 6C.

Movie S11. Minimal dynamics of GpsB-sfGFP in mature septal and equatorial rings of *Spn* cells. TIRFm was performed on strain IU11638 (*gpsB-sfgfp*) as described in SI Appendix, Experimental Procedures. Scale bar = 1 µm. Images were taken at 1 s intervals over the course of 180 s. Movie is shown at 15 frames per s. See analysis in SI Appendix, Fig. S19A.

Movie S12. Minimal dynamics of sfGFP-MltG in mature septal and equatorial rings of *Spn* cells. TIRFm was performed on strain IU11005 (*sfgfp-mltG*) as described in SI Appendix, Experimental Procedures. Scale bar = 1 µm. Images were taken at 1 s intervals over the course of 180 s. Movie is shown at 15 frames per s. See analysis in SI Appendix, Fig. S19B.

Movie S13. Active dynamics of DivIVA-GFP in mature septal and equatorial rings of *Spn* cells. TIRFm was performed on strain IU9167 (*divIVA-gfp*) as described in SI Appendix, Experimental Procedures. Scale bar = 1 µm. Images were taken at 1 s intervals over the course of 180 s. Movie is shown at 15 frames per s. See analysis in SI Appendix, Fig. S19C.

Movie S14. Septal-ring and diffusive dynamics of GFP-StkP around *Spn* cells. TIRFm was performed on strain IU9164 (*gfp-stkP*) as described in SI Appendix, Experimental Procedures. Scale bar = 1 µm. Images were taken at 1 s intervals over the course of 180 s. Movie is shown at 15 frames per s. See analysis in SI Appendix, Fig. S19D.

Movie S15. Active dynamics of HT-bPBP2x in mature septal and equatorial rings and diffusive movement in *Spn* cells. TIRFm was performed on strain IU13910 (*ht-pbp2x*) pre-labeled with a saturating amount of HT-JF549 ligand as described in SI Appendix, Experimental Procedures. Scale bar = 1 µm. Images were taken at 1 s intervals over the course of 180 s. Movie is shown at 15 frames per s. See analysis in Fig. 7A.

Movie S16. Circumferential movement of single molecules of HT-bPBP2x (red) on septa of *Spn* cells (bright-field outlines; blue border). SM-TIRFm was performed on strain IU13910 (*ht-pbp2x)* pre-labeled with a limiting amount of HT-JF549 ligand as described in *SI Experimental Procedures*. Scale bar = 1 µm. Images were taken at 1 s intervals over the course of 180 s. Movie is shown at 15 frames per s. See analysis in Fig. 7F.

Movie S17. Circumferential movement of single molecules of FtsW-HT (red) on septa of *Spn* cells (bright-field outlines; blue border). SM-TIRFm was performed on strain IU15096 (*ftsW-ht*) pre-labeled with a limiting amount of HT-JF549 ligand as described in SI Appendix, Experimental Procedures. Images were taken at 1 s intervals over the course of 180 s. Movie is shown at 15 frames per s. See analysis in Fig. 7F.

Movie S18. Single molecules of HT-bPBP2x (red) move in aberrant planes in GTPase-deficient FtsZ(G107S) mutant cells (bright-field outlines; blue border). SM-TIRFm was performed on strain IU14508 (*ht-pbp2x ftsZ*(G107S)) pre-labeled with a limiting amount of HT-JF549 ligand as described in SI Appendix, Experimental Procedures. Scale bar = 1 µm. Images were taken at 1 s intervals over the course of 180 s. Movie is shown at 15 frames per s. See analysis in SI Appendix, Fig. S22B and S22C.

Movie S19. Overproduction of FtsZ(D214A) strongly inhibits FtsZ-sfGFP treadmilling in *Spn* cells. TIRFm was performed on strain IU15181 (*ftsZ-sfgfp//*P_{Zn}-*ftsZ*(D214A)) with added 0.25 mM $ZnCl_2$ and 0.025 mM $MnSO_4$ (+Zn) to overproduce FtsZ(D214A) as described in SI Appendix, Experimental Procedures. Scale bar = 1 µm. Images were taken at 1 s intervals over the course of 120 s. Movie is shown at 15 frames per s. See analysis in SI Appendix, Fig. S25B.

Movie S20. Directional movement of single molecules of HT-bPBP2x (red) continues when FtsZ(D214A) is overproduced in *Spn* cells (bright-field outlines; blue border). SM-TIRFm was performed on strain IU15041 (*ht-pbp2x* P_{Zn}-*ftsZ*(D214A)) that was pre-labeled with a limiting amount of HT-JF549 ligand to which 0.25 mM $ZnCl_2$ and 0.025 mM $MnSO_4$ (+Zn) was added to overproduce FtsZ(D214A) as described in SI Appendix, Experimental Procedures. Images were taken at 1 s intervals over the course of 180 s. Movie is shown at 15 frames per s. See analysis in SI Appendix, Fig. S24A and S25B.

Movie S21. FtsZ-sfGFP treadmilling continues in *Spn* cells treated with 0.3 µg/mL methicillin. TIRFm was performed on strain IU9985 (*ftsZ-sfgfp*) in the presence of

methicillin at 37° C as described in SI Appendix, Experimental Procedures. Scale bar = 1 µm. Images were taken at 1 s intervals over the course of 180 s. Movie is shown at 15 frames per s. See analysis in SI Appendix, Fig. S27B and S27D.

Movie S22. Addition of 0.3 µg/mL methicillin halts circumferential movement of single molecules of HT-bPBP2x (red) in *Spn* cells (bright-field outlines; blue border). SM-TIRFm was performed on strain IU13910 (*ht-pbp2x*) that was pre-labeled with a limiting amount of HT-JF549 ligand prior to exposure to methicillin as described in SI Appendix, Experimental Procedures. Scale bar = 1 µm. Images were taken at 1 s intervals over the course of 180 s. Movie is shown at 15 frames per s. See analysis in SI Appendix, Fig. S27C and S27E.
